# Telomere Position Effect‐Over Long Distances Acts as a Genome‐Wide Epigenetic Regulator Through a Common *Alu* Element

**DOI:** 10.1111/acel.70027

**Published:** 2025-03-10

**Authors:** Raphaël Chevalier, Victor Murcia Pienkowski, Nicolas Jullien, Leslie Caron, Pascal Verdier Pinard, Frédérique Magdinier, Jérôme D. Robin

**Affiliations:** ^1^ Aix Marseille Université, INSERM, MMG, Marseille Medical Genetics U1251 Marseille France; ^2^ Aix Marseille Université, CNRS, INP, Institut de Neurophysiopathologie UMR7051 Marseille France

**Keywords:** aging, chromatin, SINEs, telomere, TPE‐OLD

## Abstract

Among epigenetic modifiers, telomeres represent attractive modulators of the genome in part through position effects. Telomere Position Effect‐Over Long Distances (TPE‐OLD) modulates gene expression by changes in telomere‐dependent long‐distance loops. To gain insights into the molecular mechanisms of TPE‐OLD, we performed a genome‐wide transcriptome and methylome analysis in proliferative fibroblasts and myoblasts or differentiated myotubes with controlled telomere lengths. By integrating omics data, we identified a common TPE‐OLD dependent *cis*‐acting motif that behaves as an insulator or enhancer. Next, we uncovered *trans* partners that regulate these activities and observed the consistent depletion of one candidate factor, RBPJ, at TPE‐OLD associated loci upon telomere shortening. Importantly, we confirmed our findings by unbiased comparisons to recent Human transcriptomic studies, including those from the Genotype‐Tissue Expression (GTEx) project. We concluded that TPE‐OLD acts at the genome‐wide level and can be relayed by RBPJ bridging Alu‐like elements to telomeres. In response to physiological (i.e., aging) or pathological cues, TPE‐OLD might coordinate the genome‐wide impact of telomeres through recently evolved Alu elements acting as enhancers in association with RBPJ.

## Graphical abstract

Proposed TPE‐OLD model. TPE‐OLD genes are defined by a progressive modulation of gene expression following telomere attrition. In our study, focusing on the molecular mechanism of TPE‐OLD, we found that the TPE‐OLD loop requires a *cis*‐signature corresponding to a newly evolved *Alu* repeats (*AluY*). The *Alu* repeat can be either in an intergenic region, as the sequence holds the same properties as an enhancer/insulator, or positioned within the gene, thus potentially affecting splicing. The DNA‐loop is maintained by *trans* partners, including SMCHD1, TZAP, TRF2, and more specifically RBPJ. As telomere shortens and DNA methylation associated with the *Alu* element decreases, the distance between the TPE‐OLD gene and telomeres increases and induces a modulation of transcription. We conclude that TPE‐OLD is regulated at three levels: (i) at the telomere through its associated length, (ii) at the *Alu* element through its associated methylation, and (iii) at the telomere‐associated proteins levels and their localization (SMCHD1, TZAP, TRF2, RBPJ). According to our work, RBPJ (directly or indirectly) along with TRF2 (directly) are the most important proteins for the maintenance of the TPE‐OLD loop.

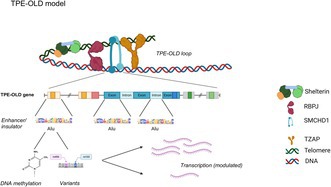

## 1 Introduction

Telomeres are unique nucleoprotein complexes composed of a 5′‐(T_2_AG_3_)_n_‐3′ motif associated with a protein complex named Shelterin (de Lange 2005). In vertebrates, a total of six proteins (e.g., TRF1, TRF2, POT1, RAP1, TPP1, and TIN2) characterize this structure located at the extremity of all chromosomes. Telomeres are also associated with other components: a long non‐coding RNA known as TERRA (TElomeric Repeat‐containing RNA) (Azzalin et al. 2007; Feretzaki et al. 2019) and a trimeric nucleoprotein complex (CST, composed of CTC1, STN1, and TEN1) binding to single‐stranded telomeric DNA (telomeric overhang) where it is involved in chromosome end capping and telomere length regulation (Lim et al. 2020). The end‐assembled telomeric structure ensures genomic stability and prevents unwanted activation of the DNA damage response (DDR) signaling and repair of DNA ends (Denchi and de Lange 2007; Galati et al. 2015; Takai et al. 2003). At each round of replication, the DNA component of telomeres shortens in all somatic cells to a threshold (i.e., short telomere length) preventing the protection mediated by shelterin units. Without this safeguard protective mechanism, cells enter replicative arrest, crisis, or apoptosis (Bendix et al. 2010; Zou et al. 2004). Interestingly, the observed association between telomeric shortening rate and proliferative rate of tissues is almost abolished during adulthood (Demanelis et al. 2020; Daniali et al. 2013). Noteworthy, telomeric proteins are not solely restricted to the aforementioned elements. Among non‐canonical functions through extratelomeric roles (Denham 2023), TRF2 and TERT have been implicated in angiogenesis (Maï El et al. 2014; Liu et al. 2016), metabolism and mitochondrial functions (Robin et al. 2020; Denham 2023) whereas RAP1 acts to protect against obesity (Martínez et al. 2013). Full identification of these molecular processes and the part taken by the telomeric components (i.e., sequences and/or proteins) remains to be explored.

Telomere dysregulation is one of the main hallmarks of aging (López‐Otín et al. 2013). Due to its implication in cancer (reactivation of telomerase, alternative lengthening) (Shay and Wright 2011; Counter et al. 1992), recent works attempted to reveal the complete identity of telomere‐associated proteins (Grolimund et al. 2013; Kan et al. 2017; Déjardin and Kingston 2009). To date, the telomere proteome identifies more than 200 proteins (Grolimund et al. 2013; Déjardin and Kingston 2009) involved in telomere functions, including novel telomeric double strand DNA (dsDNA) binding proteins such as RBPJ (Bottoni et al. 2019), HOT1 (Kappei et al. 2013; Gauchier et al. 2020) and more recently, TZAP (Jahn et al. 2017; Li et al. 2017). Recombination Signal Binding Protein For Immunoglobulin Kappa J Region, known as RBPJ or CSL, plays a central role in Notch signaling (e.g., pathways of cell fate‐decision) (Johnson and Macdonald 2011; Maicas et al. 2021) and is involved in telomere homeostasis by directly binding to the telomeric dsDNA while recruiting factors preventing chromosome end fusion (Bottoni et al. 2019). The homeobox telomere‐binding protein 1, HOT1, was identified in a proteomic high‐throughput screen targeting telomerase recruiters (Kappei et al. 2013). HOT1 acts as a bridge between telomere and active telomerase, tethering the telomere‐elongating enzyme to its target and allowing its processing. Lastly, TZAP, for telomeric zinc finger‐associated protein, has been recently described as a telomere trimming protein associated with long telomeres (with low density of shelterin units), thus setting the upper limit of telomere length (Jahn et al. 2017; Li et al. 2017). Altogether, current studies revealed that the regulation of telomere homeostasis is dynamic and complex. However, telomere shortening is mostly explored as an end point (e.g., senescence, cell crisis), when telomere reach an average critical length, rather than a continuum of dynamic lengths (Laberthonnière et al. 2019). Biological consequences throughout this dynamic process remain to be fully characterized.

Among telomere‐dependent processes, Telomere Position Effect (TPE), first described in *Saccharomyces cerevisiae* , is defined as the spreading of epigenetic marks spanning from the telomeres to adjacent genes leading to their silencing (de Bruin et al. 2000). TPE triggers a proportional continuous silencing of genes depending on the distance from telomeres and telomere length (Baur et al. 2001). Since then, this position effect has been described in human with only one gene identified so far in vivo, *DUX4*, using myoblasts from patients and their non‐affected siblings (Stadler et al. 2013). Reminiscent of observations made in yeast, we reported the existence of another type of position effect dubbed TPE‐Over Long Distances (TPE‐OLD) (Robin et al. 2014). TPE‐OLD involves the formation of telomere length‐dependent chromatin loops encompassing telomeres and subtelomeric genes. If TPE is restricted to a distance of a few kilobases (kb) from telomeres, the princeps description of TPE‐OLD identified genes within 10 Megabases (Mb) from telomeres whose expression is directly impacted by telomere length, without any restrictions towards modulated expression (either up or down) by opposition to the repressive mechanism of TPE. Despite its identification and recent studies suggesting the genome‐wide influence of telomere (Robin et al. 2014; Robin and Magdinier 2016; Mukherjee et al. 2018; Dong et al. 2021), TPE‐OLD features remain only partly characterized.

Here we sought to investigate the molecular basis of TPE‐OLD via high‐throughput transcriptomic and epigenetics analyses using the same model that led to the discovery of TPE‐OLD (Robin et al. 2014). We provide a more complete understanding of the genome‐wide impact of TPE‐OLD by identifying a common DNA motif shared by genes and DNA methylated regions modulated by telomere length across cell types. This *cis* element acts as an insulator/enhancer and shares association with telomere‐associated proteins. Our results broaden our view of TPE‐OLD at the genomic scale, opening the path to unthought‐of implications for telomeres in aging and pathologies.

## 2 Materials and Methods

### 2.1 Cell Culture

Myoblasts and fibroblasts were immortalized with a floxable hTERT cassette as described previously (Stadler et al. 2013; Robin et al. 2014). The human embryonic kidney 293 (HEK293) cell line was obtained from ATCC (CRL‐1573). For day‐to‐day maintenance, human myoblasts were seeded in dishes coated with 0.1% pigskin gelatin in 4:1 Dulbecco modified Eagle medium/Medium 199 supplemented with 15% FBS, 0.02 M HEPES, 1.4 mg/L vitamin B12, 0.03 mg/L ZnSO4, 0.055 mg/L dexamethasone, 2.5 μg/L hepatocyte growth factor, and 10 μg/L basic fibroblast growth factor. Myogenicity was verified by myotubes formation following a change to differentiation medium (2% horse serum in 4:1 Dulbecco modified Eagle medium: Medium 199) when 70%–90% confluent. Fibroblasts and HEK293 cells were grown in DMEM with L‐alanyl‐L‐glutamine (GlutaMAX TM I), D‐glucose, and sodium pyruvate (Life technologies). Media were supplemented with 10% fetal bovine serum (FBS, Gibco). All cultures were maintained in a 5% oxygen environment and passaged at ~60% confluency. Population doublings (PDs) were calculated as

$$\mathrm{PD}=\ln\left[\left(\text{final number of cells}\right)/\left(\text{initial number of cells}\right)\right]/\text{In}\left(2\right).$$

#### 2.1.1 Generation of Isogenic Clone

Cells were conditionally immortalized with CDK4—neomycin (myoblasts) and Lox‐hTERT‐TK—hygromycin (myoblasts, fibroblasts). To generate subclones with different telomere lengths, the Lox‐hTERT was excised by Cre recombinase. Absence of the hTERT construct was confirmed by testing selected clones for hygromycin sensitivity and ganciclovir resistance. Lack of telomerase activity was further confirmed by ddTRAP. To ensure the same time in culture for the generation of cell line series with various telomere lengths (4, 6, 8, 10 and 12 kb; myoblasts), hTERT removal was induced sequentially five times in the parental isogenic clone (telomerase positive). Hence, the first hTERT removal is performed to obtain the shortest telomere length (4 kb), the latest for the longest (12 kb). Importantly, all clones are kept in culture throughout the generation of the telomere set to reach the expected telomere length by cell division and telomere shortening. For myoblasts, the generation of a full set of conditions (e.g., five different mean telomere lengths) takes 90 days on average from the first hTERT removal to the 5th excision. Due to the intricate planification and caution required for generating clones, a complete set of cells is frozen after each hTERT removal. Once the last excision is performed and absence of telomerase activity confirmed, the full set is expended for respective assays and frozen for future experiments. Thaw/freeze cycles of vials were limited to one and time of assays to 20 additional days in cell culture (post selection and amplification, accounting for an additional 12 + 10 days). Last, a retrospective validation of hTERT removal is performed thanks to telomere shortening (TRF) and each cell line reaching senescence (up to 380 days in culture).

### 2.2 Telomere Restriction Fragment Analysis (TRF)

Terminal restriction fragment assay was done as previously described with a slight modification from Robin et al. (2014). Briefly, genomic DNA was digested overnight using a cocktail of 6 enzymes (*Rsa*I, *Hinf*I, *Msp*I, *Hha*I, *Hae*III, and *Alu*I), subsequently purified and ethanol‐precipitated. Next, 2 μg of digested DNA were run by electrophoresis in 0.8% agarose gel (1× TAE) for 17 h at 18 V. Gel was treated with denaturation and neutralization buffer before transfer to a positive charged Nylon membrane. After an overnight incubation with a DIG‐Telo probe (5‐10 ng/mL) resuspended in DIG Easy Hyb Buffer (Roche) at 42°C, the membrane was washed twice with washing buffer (0.5x SSC; 0.1% SDS) at 68°C before following the procedure provided by the DIG revelation Kit, using anti‐DIG alkaline phosphatase and CDP substrate (Roche). Images were obtained with Syngene CG2 bioimager.

#### 2.2.1 Telomeric DIG Probe

Probe was generated by PCR with a mix composed of dNTPs complemented by alkali labile DIG‐dUTP and genomic DNA (1 ng) with primers used for telomeric qPCR analysis (i.e., Tel1b: 5′ CGG TTT GTT TGG GTT TGG GTT TGG GTT TGG GTT TGG GTT 3′ and Tel2b: 5′ GGC TTG CCT TAC CCT TAC CCT TAC CCT TAC CCT TAC CCT 3′).

### 2.3 ddTRAP

Telomerase activity was detected using the published ddTRAP protocol (Ludlow et al. 2014). Briefly, harvested cells were lysed in lysis buffer containing 10% glycerol, 1% NP‐40, 5 mM 2‐ME, 1 mM Tris–HCl (pH 8.0), 1 mM MgCl_2_, 1 mM EDTA, 0.25 mM Sodium Deoxycholate, 0.15 mM NaCl, and 0.1 mM AEBSF. Lysate was then used for TS extension, using 5000 cells equivalent and a 45 min incubation at 25°C in TRAP buffer containing 200 mM Tris–HCl (pH 8.3), 15 mM MgCl_2_, 630 mM KCl, 0,5% Tween20, and 10 mM EGTA. A heat‐kill step at 95°C for 5 min was added to denature remaining active telomerase. Extended products were then processed using the ddTRAP amplification reaction (EvaGreen) in a QX 200 ddPCR apparatus (Bio‐Rad, Hercules, CA, USA). Droplets were generated and processed following the PCR profile: 95°C—5 min; 39 cycles of 95°C—30s, 54°C—30s, 72°C—30s; and 12°C hold. Next, the amplified droplets were read and analyzed using the ddPCR reader and QX Manager software. Primer sequences used for the extension and detection are as follows: TS primer: 5′ AAT CCG TCG AGC AGA GTT 3′ and ACX primer: 5′ GCG CGG CTT ACC CTT ACC CTT ACC CTA ACC 3′. For each assay, U87, a glioblastoma cell line, was used as a positive telomerase control; no extension and no template conditions were used as negative controls.

### 2.4 DNA Extraction and Sodium Bisulfite Sequencing

DNA was extracted using the NucleoSpin Tissue (Macherey‐Nagel) according to the manufacturer's instructions. For sodium bisulfite sequencing, 1 μg of genomic DNA was denatured for 30 min at 37°C in NaOH 0.4 N and incubated overnight in a solution of 3 M Sodium bisulfite pH 5 and 10 mM Hydroquinone. Converted DNA was purified using the Wizard DNA CleanUp kit (Promega) following manufacturer's recommendation and precipitated by ethanol precipitation for 5 h at −20°C. Primers were designed in order to amplify methylated and unmethylated DNA with the same efficiency using the MethPrimer software, avoiding the presence of CpGs in the primer sequence. After deep sequencing (> 50 K sequences retrieved), sense and antisense sequences were assembled in a single sequence and the bam file was converted to a fastq file. After trimming, data were aligned using the BiQ Analyzer HiMod software (http://biq‐analyzer.bioinf.mpi‐inf.mpg.de) and processed in R (version 3.4.2). BiQ Analyzer HiMod converts sequencing data by using “1” for a methylated CG, “0” for unmethylated, and “X” in case of misalignment.

#### 2.4.1 Infinium MethylationEPIC Array

Genome‐wide DNA methylation analysis was performed by using the V1 Infinium MethylationEPIC Array through Diagenode services. Genomic DNA was extracted using the NucleoSpin Tissue kit (Macherey‐Nagel) from two different cell pellets for each sample. The analysis was mainly carried out using the ChAMP R package and treated as described previously (Laberthonnière et al. 2023). The default cut off *p*‐value for DMRs to be selected is 0.05, and they must contain at least seven probes and exceed 50 bp in width. The Combat method was used to correct for batch effects. Heatmaps were realized using the pheatmap (v1.0.12) R package using the Beta value matrix for methylation. Genomic localizations of DMRs were represented as Circos using Circulize v0.4.15. NCBI. Gene Expression Omnibus (https://www.ncbi.nlm.nih.gov/gds) DNA methylation data accession number: GSE213427.

### 2.5 RNA Sequencing

Total RNA was extracted using the RNAeasy kit (Qiagen) following the manufacturer's instructions. Only samples with a RIN > 9 were kept for further use. Libraries were constructed using 2 μg of total RNA. The TruSeq Stranded mRNA Library Preparation Kit High Throughput (Illumina; #RS‐122‐2103) was used according to the manufacturer's guidelines. Libraries were quantified by qPCR using the KAPA Library Quantification Kit for Illumina Libraries (Roche; #7960140001) and profiles were assessed using the DNA High Sensitivity LabChip Kit (Agilent Technologies; #5067‐4626) on an Agilent Bioanalyzer 2100. Libraries were sequenced on an Illumina Next‐seq 500 2×75bp at the GBiM genomic core facilities (https://www.marseille‐medical‐genetics.org/fr/genomics‐bioinformatics‐platform/).

#### 2.5.1 RNA‐Seq Data Processing and Differential Expression Analysis

Trimmed single‐end reads were aligned using STAR v2.5.3a to the GRCh38 human genome release. Obtained BAM files were indexed using Sambamba (v0.6.6). Gene Expression Omnibus (https://www.ncbi.nlm.nih.gov/gds) RNA Sequencing data accession number: GSE213281. Aligned reads were counted with StringTie v1.3.1c using GENCODE annotation. DEGs of different conditions were identified using R package DESeq2 (v1.18.1) with the following thresholds FDR < 0,05, abs (LogFC) > 2.

#### 2.5.2 DEGs Analysis

DEGs were represented as Circos using Circulize v0.4.15 for visualization. Overrepresentation test analyses were performed using enrichGO from the R package clusterProfiler (v3.10.15). With DEGs as input, we identified biological processes (BP) with an FDR < 0.05 using a custom gene universe based on genes with a row mean for counts > 1. Results are presented as barplot with on the bottom *x*‐axis, the log_10_(FDR), corresponding GO terms and on the top *x*‐axis, the percentage of DEGs associated with a GO‐term.

#### 2.5.3 RT‐qPCR

RNA extraction was realized using Qiagen's RNeasy mini kit. Briefly, reverse transcription of 1 μg of total RNA was performed using the Superscript IV kit and oligo dT following manufacturer's instructions (Life Technologies). PCR amplification was performed on a StepOnePlus Real‐Time PCR system (Life Technologies) using the SYBR green master mix with the following program: Pre‐incubation at 95°C for 10 min, then 40 cycles of amplification, each corresponding to 15 s at 95°C followed by 1 min at 60°C. The program ends with a melting step including a step of 1 s at 98°C, 30 s at 70°C, and finally 10 s at 98°C. Crossing‐threshold (*C*_t_) values were normalized by subtracting the geometric mean of three housekeeping genes (*GAPDH*, *PPIA* and *HPRT*). All *C*_t_ values were corrected by their PCR efficiency, determined by 1:2 or 1:4 cDNA dilution series. All analyses were carried out in biological duplicates and technical duplicates unless specified otherwise. Primer sequences are provided in the Supporting Information.

### 2.6 De Novo Motif Analysis

Motif analysis was performed using MEME (v5.1.1) on identified DMRs, DEGs coordinates using a fasta file or CHIP‐seq peaks from available datasets (TRF1 and TRF2, GSE26005; TZAP, GSE96778; HOT1, GSE46237; RBPJ, GSE29498). Motifs reported were selected for an *E*‐value < 0.05 using markov order 1 (for bias in CG) parameters for DMRs.

*meme file*.*fa ‐o output ‐dna ‐mod anr ‐nmotifs 10 ‐evt 0*.*05 ‐minw 6 ‐maxw 35 ‐markov_order 0/1 ‐V ‐maxsize 20000000 ‐p 10*.

### 2.7 Nuclear Protein Extraction

Nuclear protein extraction was performed using NE‐PER Nuclear and cytoplasmic extraction (Thermo Fisher Scientific; #78835).

### 2.8 Electrophoretic Mobility Shift Assay

EMSA was performed using either 2% agarose or 10% polyacrylamide gels. Revelation for EMSA in polyacrylamide gel with biotin‐incorporated dsDNA fragment (WE) was performed using the LightShift Chemiluminescent EMSA Kit (Thermo Fisher Scientific; #20148).

### 2.9 Transfection and Flow Cytometry

#### 2.9.1 Plasmid Construction

pCMV derived plasmids (i.e., pCMV and pCMV‐Telo) are described in (Ottaviani et al. 2009). Briefly, DNA fragments were cloned downstream of the eGFP reporter gene in pCMV vectors after *Asc*I linearization (NEB). Sanger sequencing was performed for all selected clones containing the insert in a 5′ to 3′ orientation (exception of the EW sequence). Validated plasmids with targeted sequences were then grown for amplification, and DNA was purified using the NucleoBond Xtra Midi kit for transfection‐grade plasmid DNA (Machery‐Nagel).

#### 2.9.2 Plasmid Transfection

Transfection of the linearized vectors was performed using a modified calcium phosphate method optimized in order to obtain a single integration per cell (Koering et al. 2002). Three days post‐transfection, Hygromycin B antibiotics was added to the culture medium (Life technologies) for selection at a final concentration of 400 μg/mL. Cells were kept under permanent selection for several passages (3 weeks on average) to ensure complete selection and stable eGFP expression.

#### 2.9.3 Flow Cytometry

eGFP expression was analyzed using an Accuri flow cytometer and processed using the FlowJo software (Becton‐Dickinson). For each construct and condition tested, eGFP expression was recorded for 3 consecutive weeks in biological quadruplicates (i.e., four independent transfections) for a minimum of 12 measures per condition. The percentage of eGFP‐positive cells was determined using the corresponding non‐transfected cells as the baseline for autofluorescence. Mean values (M1) were used to compare fluorescence in the different samples.

### 2.10 3D DNA‐FISH

Three‐dimensional DNA Fluorescent in situ Hybridization was performed as previously described (Robin et al. 2014). For HEK 293, we used a probe generated by Nick translation (Abbott Molecular) using the pCMV or pCMV‐Telo plasmid as a template; for myoblasts, we used commercial probes for telomeres (FITC‐labeled C‐Rich telomere probes, Eurogentec) and *GLIS2* (RP11‐295D4, EmpireGenomics). Images were acquired using a confocal system (LSM800, Zeiss). A 63x Plan‐Apochromat oil immersion objective was used to record optical sections at intervals of 0.24 μm. The pinhole was set to 1 Airy with optical slices in all wavelengths with identical thickness (0.4 μm). Generated.lsm files with a voxel size of 0.1 μm × 0.1 μm × 0.24 μm were processed using the IMARIS software (Bitplane, AG). After 3D reconstruction, we report the distance between the gravity center of the probe signals for a minimum of 30 nuclei per samples (i.e., 60 alleles).

### 2.11 siRNA Transfections

Transfections were performed using DharmaFECT1 Transfection reagents and siRNAs (SMARTpool ON‐TARGETplus Human, 5 nmol; Horizon discovery) targeting *TERF2* (ID: 7014); *RBPJ* (ID: 3516); *TZAP* (ID: 3104); *CTCF* (ID: 10664); *SMCHD1* (ID: 23347) along with a Non‐Targeting Control, NT (D‐001810‐0X). Transfections were carried on following the protocol and conditions provided for HEK293 or myoblasts by the manufacturer (Horizon discovery). HEK cells were collected 24, 48, and 72 h post‐transfection, and 72 h post‐transfection for myoblasts prior to RNA and DNA extraction. For the 3D DNA‐FISH assay, myoblasts were fixed and processed 72 h post‐transfection.

### 2.12 Chromatin Immunoprecipitation Quantified by ddPCR (ChIP‐ddPCR)

Cells were crosslinked for 10 min at room temperature and 20 min at 4°C with 0.8% formaldehyde (methanol free, ultrapure EM grade, Polysciences Inc.; Warrington PA). Reaction was stopped for 10 min at room temperature with the addition of Glycine to a final concentration of 0.125 M. Cells were then rinsed twice with ice‐cold 1X PBS, scraped from the dish, and pelleted by centrifugation (800 g, 5 min at 4°C). Next, cells were treated according to the manufacturer's instructions (Pierce Classic Protein G IP Kit, Thermo Fisher Scientific). For sonication, we used a total processing time of 15 min per sample in a Bioruptor (Diagenode) using the following settings: 15 cycles; 30 s ON/30 s OFF on High power. Sonicated DNA was controlled on a 2% agarose gel; adequate sonication is achieved when a smear ranging from 200 to 700 bp is obtained. For immunoprecipitation (IP), prepared chromatin was complemented either with H3 (Abcam ab1791); TZAP (GeneTex GTX 118671); RBPJ (Invitrogen 720219); SMCHD1 (equal mix Abcam ab31865 and Sigma HPA03944); TRF2 antibody (Imgenex124A) or IgG (Milipore PP64B) at recommended concentration (between 1.5 and 5 μg) according to manufacturer's instruction. IPs were incubated overnight at 4°C with rotation. For each reaction, 1 μL (IP, IgG, 1% input) were used as controls for ddPCR analysis. Primers were designed for the WE‐associated region of each gene; results are adjusted to inputs and further normalized to H3 IP. Each PCR primer pairs were tested on genomic DNA to verify specificity and efficiency.

### 2.13 HiC Data‐Mining

We retrieved all available HiC data from the HFFc6 cell line (immortalized fibroblasts) published in a recent work (i.e., *n* = 16 HiC runs; GSE163666) (Akgol Oksuz et al. 2021) and applied a custom‐made pipeline (provided in the supplemental) aimed to only retrieve the interactions involving Telomeres. Internal telomeric sequences (e.g., less than 7 TTAGGG repeats) were discarded, and the window frame was increased to 100pb upstream and downstream reads to search for motif.

### 2.14 Telomeric Induced Foci (TIFs)

Cells were grown on coverslides and fixed for 10 min on ice with 3.7% formaldehyde. Cells were then washed in PBS (3min × 5min) and permeabilized with 0.5% Triton X‐100 for 1 h at room temperature. Next, cells were washed twice with SSC 2× for 5 min at room temperature and subsequently treated with RNase A for 45 min at 37°C. After an additional SSC 2× wash (5 min). Hybridization of the PNA probe was performed for at least 2 h at 37°C after denaturation (5 min, 85°C) in 70% formamide, 10 mM Tris pH 7.2, 1 μL of FITC‐labeled C‐Rich telomere probes (50 nM, Eurogentec) and 1% blocking solution (Roche). Cells were serially washed, blocked for 1 h, and immuno‐stained overnight at 4°C in the blocking solution containing the primary rabbit polyclonal anti‐53BP1 antibody (1:500; Novus Biologicals). After three washes with PBS/0.1% Triton X‐100, slides were incubated for 1h30min with Alexa 555 Donkey anti‐rabbit secondary antibody in PBS containing 0.5% Triton X‐100, 1% BSA, and 2.5% donkey serum. Slides were mounted in Vectashield with DAPI (Vector Laboratories, Burlingame, USA). Images were taken using a Confocal system (LSM800, Zeiss). Co‐localization events, representing telomeric DNA damages (TIFs), were counted in at least 30 nuclei per condition from three independent experiments using the IMARIS software.

### 2.15 Western Blotting

After two PBS washes, confluent cells were scrapped in PBS, pelleted, and stored until extraction at −80°C. Total protein extraction from cell pellets was achieved with NP40 lysis buffer or SDS lysis buffer (required for RBPJ), followed by brief sonication. Separation of proteins by SDS‐PAGE using NuPAGE 4%–12% gradient Bis‐Tris gels (Invitrogen, NP0322BOX) and MOPS SDS running buffer (Invitrogen, NP0001‐02), and western blotting of separated proteins on nitrocellulose were performed as described previously (Verdier‐Pinard et al. 2017). Antibodies used are detailed in the ChIP‐ddPCR section with the addition of TRF1 (Abcam, ab10579), CTCF (Abcam, ab128873) and Tubulin (Abcam, ab7291).

### 2.16 Statistical Analysis

All experiments were repeated at least three times, with three at least biological replicates, unless specified otherwise. Quantitative data are displayed as means ± standard error of the mean or ± standard deviation when notified. Sample sizes as well as the statistical test used for each experiment are described in each corresponding figure legend or method. Results from each group were treated with the GraphPad prism software for all statistical tests. All tests were two‐sided and alpha was set at 0.05. Only *p*‐values less than 0.05 were considered statistically significant.

## 3 Results

### 3.1 Changes in DNA Methylation but not Transcription Occur at Subtelomeres Upon Telomere Shortening

**FIGURE 1:**
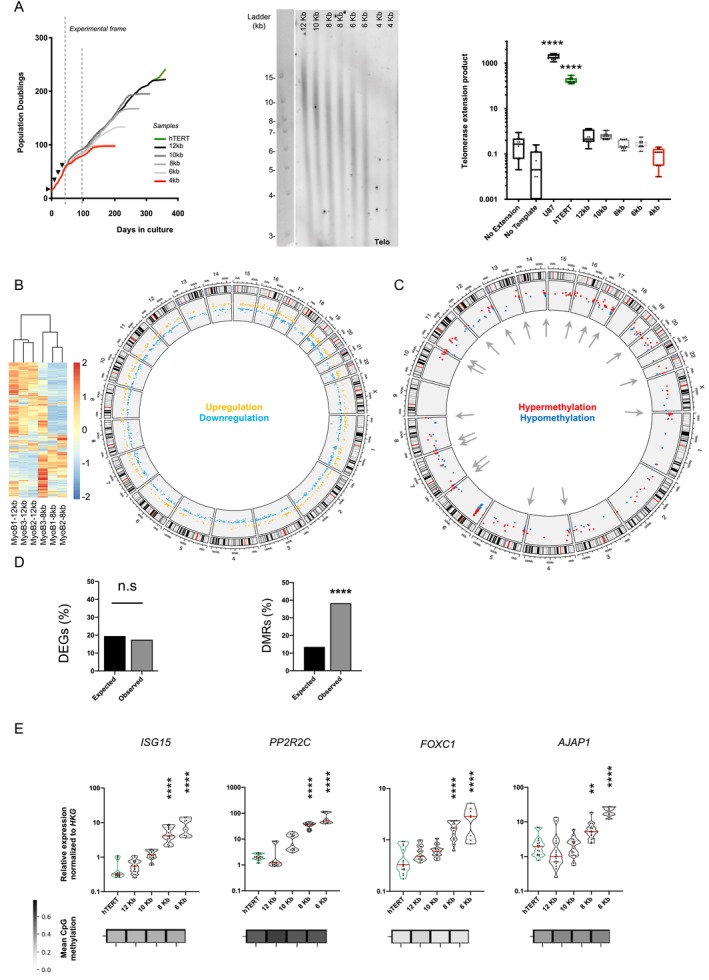
Marked DNA methylation changes at subtelomeres upon telomere shortening. (A) Growth curve‐associated telomere restriction fragment analysis (TRF) and droplet digital telomere repeat amplification protocol (ddTRAP) of the cellular model used for the study (myoblasts). Time points of hTERT excision are symbolized by black triangles along with the frame used for experiments by dashed lines. Isogenic clones, reported with their estimated telomere lengths and telomerase activity, were explored before any sign of DNA damage (4Kb, represented in red; Figure S1). For ddTRAP, we report negative (no extension, no template) and positive controls (U87 cell line) and representative results of biological triplicates in technical quadruplicates (*n* = 12). Medians and quartiles are shown (red and dashed lines, respectively). Holm‐Sidak's multiple comparison test; *α* = 0.05. p**** < 0.0001. (B) Heatmap of unsupervised clustering of differentially expressed genes (DEG) in biological triplicates of isogenic myoblasts clones with long (12 kb) or shorter telomere (8 kb) along with a Circos representation of DEGs distribution across the genome. Significantly upregulated genes are reported in yellow while downregulated genes are in blue. (C) Repartition of differentially methylated regions (DMRs) across the genome in isogenic myoblasts clones with long (12 kb) or shorter telomere (8 kb). Significantly hypermethylated regions are reported in red while hypomethylated regions are in blue. Arrows (gray) point to DMRs located at subtelomeres. (D) Proportion of expected DEGs in subtelomeric regions compared to observed DEGs (left panel) along with the proportion of expected DMRs in subtelomeric region compared to observed DMRs (right panel) in our dataset. Only subtelomeric DMRs are statistically enriched. Geometric test (DEGs), *χ* ^2^ test (DMRs); *α* = 0.05. p**** < 0.0001. (E) Associated expression of selected genes (RT‐qPCR) and their mean associated CpG methylation in myoblasts. For gene expression, results are normalized to three housekeeping genes (*HKG*: *HPRT*, *PPIA* and *GAPDH*) and respective expression in isogenic clones with long telomeres (12 kb). Reported methylation represents the average associated CpGs values of gene (full sequence). For each condition, we report the average of three independent isogenic clones with technical duplicates. Medians, quartiles, and all data points are shown in violin plots. Holm‐Sidak's multiple comparison test; *α* = 0.05. p** < 0.005; p**** < 0.0001.

To uncover the molecular basis of TPE‐OLD, we exploited the strategy applied in earlier studies (Stadler et al. 2013; Robin et al. 2014, 2015). Briefly, cells are immortalized using a floxable *hTERT* cassette and an additional CDK4 construct when required (i.e., myoblasts). Immortalized cells are then grown to homogenize telomere lengths across chromosome ends (92 ends) and *hTERT* is sequentially removed from the parental cell line to generate isogenic clones (indicated by black triangles, Figure 1A). To confirm the telomere length status and absence of residual elongation, cells are monitored for telomerase activity and telomere lengths by ddTRAP and TRF (Figures 1A and S1a). Importantly, all cells roughly spend the same time in culture, thus minimizing effects imputable to cell culture conditions (Bork et al. 2010; Franzen et al. 2021).

For myoblasts and myotubes, we used isogenic clones with an average telomere length of 12, 10, 8, 6 or 4 kb and 14 or 8 kb for fibroblasts (Figures 1A and S1a). Of note, DNA damage signals detected by γH2AX staining were significant only in myoblasts with the shortest average telomere length (4 kb, Figure S1b,c). Then, we performed a transcriptomic analysis in myoblasts with long (12 kb) and shorter telomeres (8 kb) along with a methylome analysis using an EpicArray approach in myoblasts with long and shorter telomeres (12, 10, 8, 6, 4 kb; respectively), as DNA methylation is acknowledged as a stable epigenetic mark. Cells with an average telomere length of 4 kb were included as a cell “crisis” time point and were removed from later observations, unless explicitly mentioned.

From the transcriptomic analysis, only two “early stages” (12‐8 kb) of telomere shortening kinetics were analyzed in order to capture effects mostly generated by epigenetic changes associated with telomere shortening rather than changes linked to senescence. Differentially expressed genes (DEGs) were found across all chromosomes and allowed the clustering of myoblasts relative to their average telomere length (Figure 1B). Regarding biological pathways (BPs) enriched in myoblasts with shorter telomeres, we note that BPs associated with upregulated genes are related to muscle fusion, function, and contraction, whereas BPs associated with downregulated genes are involved in cell migration (Figure S2). A principal component analysis (PCA) of isogenic clones of myoblasts, along with their myotubes counterparts, showed that myoblasts with short telomeres are closer to myotubes than their isogenic clones with long telomere (i.e., myoblasts, Figure S2d). These results suggest that shorter telomere lengths might trigger the premature expression of marker of post‐miotic differentiated myotubes. Future studies are required to confirm this trend.

Methylation analysis of 850 K CpGs across the genome revealed an overall trend towards hypermethylation in cells with shorter telomeres (Figure S3a), without overrepresentation of specific regions (e.g., CpG island) nor functional elements (e.g., 3′UTRs, TSS; Figure S3b,c). This trend is consistent with previous reports (Bigot et al. 2015). Similar to transcription results, we report several Differentially Methylated Regions (DMRs) across the genome (Figure 1C, Table S1). Further, we inquired within our data for DMRs directionally and steadily modulated with telomere attrition. Only DMRs with constant hyper‐or hypomethylation across conditions (12 vs. 10; 10 vs. 8 and 8 vs. 6) were kept. We observed that 38.3% of DMRs are located in subtelomeric regions (gray arrows, Figure 1C).

Next, to evaluate if subtelomeric regions were enriched in either transcriptomic or methylation changes, we determined the expected percentage of DEGs (corresponding to the proportion of genes located in subtelomeres; i.e., 20%) and DMRs compared to the results obtained from our transcriptomic and methylome analyses (Figure 1D, geometric test, *p* = 0.99; *χ* ^2^ test, *p* < 0.0001; respectively). Further, using additional series of isogenic clones with long and short telomeres (Figures S1 and S4), we confirmed the gradual upregulation of known TPE‐OLD genes by RT‐qPCR (e.g., *ISG15*, *PPP2R2C*) (Jäger et al. 2022; Robin et al. 2014) (Figure 1E). We obtained the same DNA methylation profile in two additional cell types (i.e., myotubes, fibroblasts; Figure S5, Table S1). DMRs associated with variable telomere lengths are enriched in subtelomeres. This suggests that telomere attrition first impacts DNA methylation. Howbeit, future investigations are needed to precisely determine the influence of telomere erosion on DNA methylation and reciprocally.

### 3.2 TPE‐OLD Acts as a Genome‐Wide Epigenetic Regulator Through a Unique DNA Motif

**FIGURE 2:**
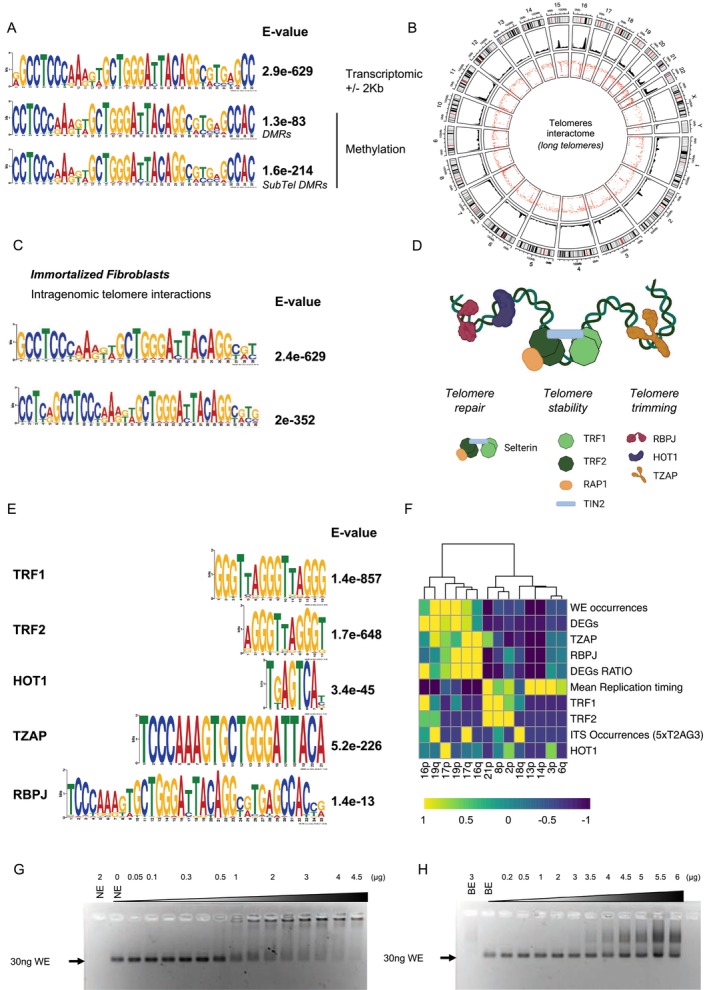
Transcriptomic and DNA methylation changes upon telomere shortening are associated with a unique DNA motif. (A) Motif logos of the top sequences present in direct proximity to either DEGs (± 2 kb), DMRs, or DMRs located in subtelomeres identified from data collected in isogenic myoblasts with long (12 kb) and shorter (8 kb) telomeres. For each motif, we report the associated E‐value. (B) Circos and rain drop representation of telomeric‐associated interaction found in multiple HiC runs from HFFc6 cells (long telomere cells; *n* = 16) reported in Akgol Oksuz et al. (2021). (C) Motif logos of the top sequences present in the telomere‐genome interactions illustrated in (B) and their associated *E*‐values. (D) Graphical representation of the main telomere‐associated proteins binding to dsDNA and their main involvement in telomere homeostasis. (E) Motif logos and associated *E*‐values of the top sequences present in previously published ChIP‐Seq experiments (Hammal et al. 2022). We report the most abundant motifs associated with each dsDNA binding telomeric‐associated protein. (F) Unsupervised hierarchical clustering (WardD2; Manhattan distance) of specific subtelomeres (10 Mb) with motif occurrences (WE), DEGs, and telomere‐associated factors (replication timing, proteins, Internal Telomeric Sequences ITS). Parameters were extracted from available datasets and normalized to a mean of 1. For telomere replication timing, the lowest value (i.e., −1) corresponds to early replication; the highest (1) to a late replication. We report DEGs as total DEGs localized at each subtelomere and DEGs ratio when corrected for gene density (the complete set is reported in Figure S6). (G, H). Electromobility Shift Assay performed in a 2% agarose gel. For each assay, 30 ng of the double‐stranded DNA corresponding to the 35 bp motif was loaded and run with increasing amounts of either nuclear (G) or bacterial (H) extract. A shift happens if dsDNA binds to the extract content and is only visible in the experiment using nuclear extracts.

Next, we searched for a common signature by crossing the transcriptome and methylome data. We compared DEGs and DMRs. We identified a 35 bp DNA sequence (5′‐CCTCCCAAAGTGCTGGGATTACAGGCGTGAGCCAC‐3′) shared by genes or DMRs enriched in both datasets (Figure 2A). We dubbed this motif “WE” for Wide Effect motif. To confirm our newly discovered motif, we took advantage of recently generated HiC data using the HFFc6 cell line (*n* = 16; immortalized fibroblasts with long telomeres) (Akgol Oksuz et al. 2021). The HiC assay generates a genome‐wide 3D chromatin interaction map. In order to highlight recurrent telomeric interactions, we applied a built‐in pipeline recovering only telomeric‐associated interactions that are found across datasets (Figure 2B). Strikingly, this novel bioinformatic approach revealed interactions involving known TPE‐OLD genes such as *ISG15*, *SORBS2*, *C1S*, or *PPP2R2C* (Jäger et al. 2022; Robin et al. 2014, 2015). To further challenge our findings (i.e., *cis* motif), we then considered enriched sequences involved in these telomeric‐associated interactions (Figure 2C). As a reinforcement of our previous observations, we found our newly described signature motif (WE; Figure S6a).

Stemming from previous work describing the influence of telomeres over the genome (Mukherjee et al. 2018; Kim et al. 2016; Robin et al. 2014, 2020) and taking advantage of additional available datasets (ChIPSeq) (Simonet et al. 2011; Kappei et al. 2013; Hammal et al. 2022), we compared the WE sequence to the main motifs enriched in selected datasets. We narrowed down our screen to curated ChIPSeq data for dsDNA binding proteins associated with telomeres, each with a described role in telomere homeostasis (Kappei et al. 2013) (Figure 2D). As predicted, the main motif bound by shelterin proteins (TRF1, TRF2) corresponds to the canonical T_2_AG_3_ telomeric sequences and HOT1 to a small 5′‐TGAGTCA‐3′ motif. Singularly, TZAP and RBPJ, two proteins associated with telomere trimming and repair, showed an enrichment for a motif highly similar to WE (Figure 2E). Due to its unique ds‐ssDNA binding junction role (Tesmer et al. 2023), POT1 was not included in our restrictive list of dsDNA proteins.

To further investigate the association of the WE motif and telomeres homeostasis, we detailed the repartition of WE to DEGs; DEGs ratio (normalized to the total number of genes present at telomeres); the presence of Internal Telomeric Sequences (ITS) and telomere replication timing (Arnoult et al. 2010; Piqueret‐Stephan et al. 2016), respective to chromosome ends (Figures 2F and S6b). Unsupervised clustering, at the chromosome end level, confirmed the correlation between WE, RBPJ and TZAP and revealed an unexpected inverse relationship with telomere replication timing.

Additionally, because telomeres are restricted to eukaryotes, we tested the WE binding specificity by EMSA using either nuclear (Figures 2G and S6c) or bacterial (Figure 2H) extracts. We observed that DNA‐protein complexes were specific and restricted to assays using nuclear extracts.

Altogether, our data suggest a common *cis* motif associated with TPE‐OLD genes enriched in TZAP and RBPJ. This motif is also strongly associated with early replicating telomere ends. This hints towards an organized repartition of TPE‐OLD gene, respective to specific telomere ends.

### 3.3 TPE‐OLD Motif Signature Displays an Enhancer/Insulator Activity

**FIGURE 3:**
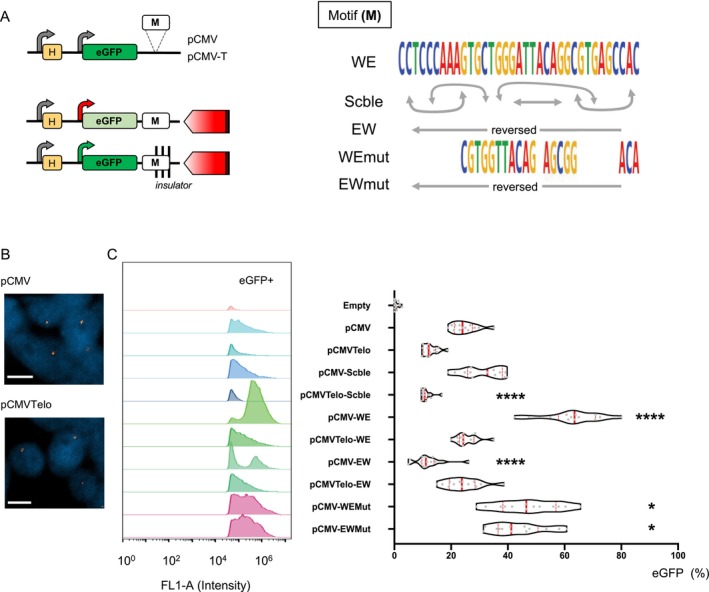
The Associated TPE‐OLD motif presents an enhancer/insulator activity. (A) Graphical representation of the construct (left) and DNA sequences used (right) for reporter assays. Briefly, sequences to test are inserted at a multiple restriction site (M) located directly after the *eGFP* reporter gene and upstream of a telomere seed for pCMV‐T. Each construct (WE; Scble; EW; WEMut; EWMut) is transfected, and stable populations of cells are selected in the presence of Hygromycin. Constructs allow one to test either position effects (pCMV) or telomere position effect (pCMV‐T) and the insulator‐related proprieties of the inserted motif. If the motif holds insulator/enhancer proprieties, transcription of *eGFP* is active (Green arrow, green eGFP); if not transcription is repressed (Red arrow) inducing a decreased eGFP expression (faded green). Conditions are optimized for a single insertion per cell. (B) Representative images showing single insertion of constructs (pCMV; pCMV‐T) in HEK 293 cells. (C) eGFP expression profile and corresponding quantifications determined by flow cytometry in HEK 293 transfected with various constructs. After transfection of constructs and selection, eGFP levels were measured by flow cytometry for 3 consecutive weeks. Assays were performed in quadruplicates for a total of *n* = 12 assays/construct. We report all data points in box and violins. Medians and quartiles are shown (red and dashed lines, respectively). Tamhane's T2 multiple comparison test; *α* = 0.05. p* < 0.05; p**** < 0.0001.

As opposed to TPE, TPE‐OLD is described as an epigenetic mechanism inducing either up‐ or downregulation of gene related to their most proximal telomere length. To gain insight into the direct properties of the WE motif in gene regulation (i.e., enhancer, repressor, insulator), we took advantage of well‐documented reporter constructs (Koering et al. 2002; Ottaviani et al. 2009). Briefly, our system includes a Hygromycin resistance gene and consists of an eGFP cassette driven by a pCMV promoter followed by a motif insertion site (Figure 3A). Accounting for the abundance and widespread localizations of the WE motif, we chose a strategy allowing for a random insertion of a unique copy of the motif (Koering et al. 2002). This approach is not in line with the current practices that favor a single insertion at a single specific site. Tested sequences are assessed in a singular and controlled chromatin context (Hilton et al. 2015; Li et al. 2020). Instead, our approach mimics the diversity of sites and the potential diversity in chromatin contexts surrounding the WE element (Figure 1B,C). Thus, by investigating cell populations carrying the motif‐containing reporter genes, we aim to test the genome‐wide proprieties of the inserted sequences with minimal bias. Noteworthy, due to this strategy, we are entitled to observations linked to a unique global effect (enhancer or repressor); thus, masking effects that are restricted to specific and complex chromatin contexts.

Different WE‐derived motifs were tested: WE, Scramble, reversed orientation, and mutations (Figure 3A). Further, we used an analog construct, pCMVTelo, carrying a telomere seed, forcing the insertion in a telomeric position after a telomere crisis and healing at any chromosome ends (Koering et al. 2002). The insertion of sequences in a telomeric context was performed to distinguish TPE from TPE‐OLD. After controlling single insertion in HEK 293 cells by DNA‐FISH (Figure 3B), we maintained all cell populations under Hygromycin selection and recorded their eGFP expression profiles (intensity, percentage of eGFP‐positive cells; Figures 3C and S7). We report a significant increase of eGFP‐positive cells restricted to cells carrying the WE sequence (Mean ± SEM = 61.31 ± 4.35) in comparison to all others (*p* < 0.0001, Tamhane's T2 multiple comparisons test). In cells carrying the reverse motif, dubbed EW, we observed a low proportion of eGPF‐positive cells to a level lower than the control condition (*p* < 0.0001, Tamhane's T2 multiple comparisons test). Interestingly, these effects were moderated in cells carrying mutated sequences (i.e., WEMut; EWMut), with a moderate increase of eGFP‐positive cells compared to controls (*p* = 0.0159 and *p* = 0.0447, Tamhane's T2 multiple comparisons test; respectively).

In a telomeric context (pCMV compared to pCMVTelo), we observed a decrease in eGFP‐positive cells, except for the EW motif where we observed an increase in eGFP‐positive cells (*p* = 0.0013, Tamhane's T2 multiple comparisons test, Figure S7). This shows that TPE is not affected by the WE sequence.

Our results suggest that WE holds properties similar to insulators or enhancers. Interestingly, in a reversed orientation, dubbed EW, the motif seems to act as a silencer.

### 3.4 WE Activity Is Modulated by RBPJ and SMCHD1

To challenge our hypothesis regarding WE‐associated proprieties and its role in TPE‐OLD, we tested a subset of WE‐associated gene motifs. To this aim, we picked WE‐related sequences found in the vicinity of DEGs from our transcriptomic analysis (Figure 1) such as *C16Orf74* (motif length: 201 bp; distance from gene promoter: 22 kb); *BET1L* (195 bp; 1.2 kb); *GIPC3* (372 bp; 2.5 kb); *GLIS2* (413 bp; 55 kb) and inserted these gene‐related motifs in both constructs (pCMV, pCMVTelo).

**FIGURE 4:**
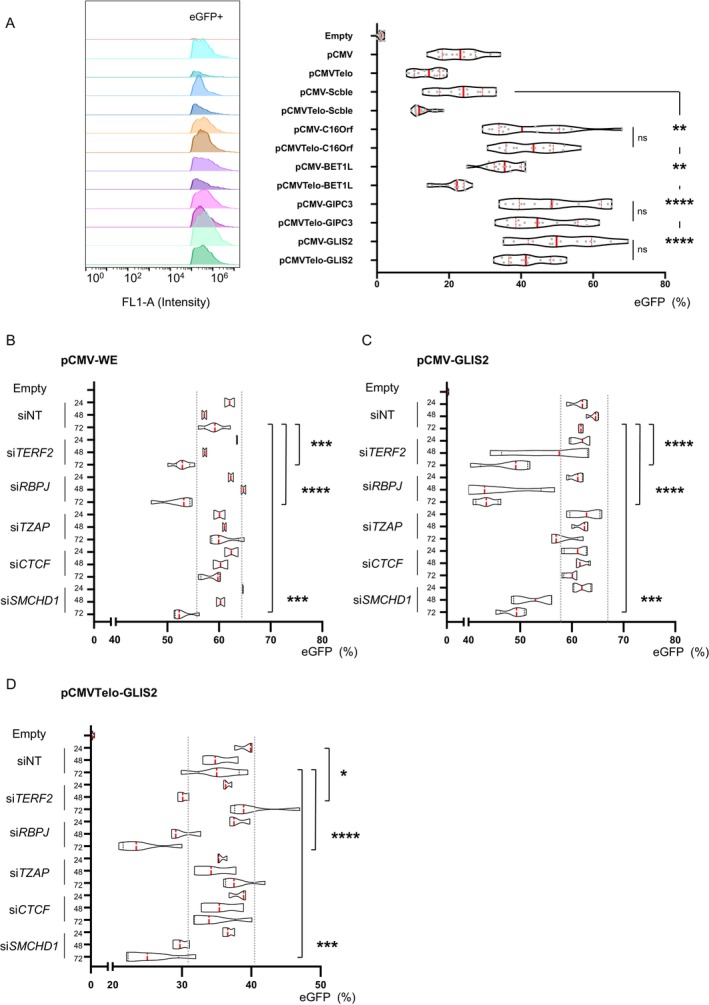
WE activity is modulated by RBPJ and SMCHD1 level. (A) eGFP expression profile determined by flow cytometry in HEK 293 transfected with reporter genes containing the motif found in proximity to DEGs (*C16orf74*, *BET1L*, *GIPC3*, *GLIS2*) and corresponding quantifications. After transfection of constructs and selection, eGFP levels were measured by flow cytometry for 3 consecutive weeks. Assays were performed in biological quadruplicate for a total of *n* = 12 assays/construct. We report all data points in box and violins. Medians and quartiles are shown (redline). (B–D). Quantification of reporter assays after transfection in HEK 293 of siRNA targeting epigenetic modulators and dsDNA telomeric binding proteins. Cells with either pCMV‐WE (B), pCMV‐Glis2 (C), or pCMVTelo‐Glis2 (D) constructs were transfected with siRNA. The proportion of eGFP‐positive cells was measured by flow cytometry at 24, 48, and 72 h post‐transfection. For each condition, we report biological quadruplicates (*n* = 4) in box and violins. Medians and quartiles are shown (red lines); dashed gray lines are shown to represent the variability associated with the NT condition (significant threshold). Tamhane's T2 multiple comparison test; *α* = 0.05. p* < 0.05; p** < 0.005; p*** < 0.001; p**** < 0.0001.

All motifs were associated with an increase in eGFP‐positive cells (Figures 4A and S8). The WE‐gene motif corresponding to *BET1L* behaved equally as WE with a decreased signal when inserted at telomeric positions (*p* < 0.0001, Tamhane's T2 multiple comparisons test) whereas, regardless of their chromosomal insertion, the three others (*GLIS2*, *C16Orf74*, *GIPC3*) did not exhibit significant changes. Our observations further advocate for an insulator/enhancer role of WE, with differences imputable to the variation respective to the WE motif found at different TPE‐OLD genes, suggesting divergences in their overall regulations.

Next, we took advantage of our set system (i.e., pCMV/pCMVTelo in HEK 293) to evaluate possible *trans* partners associated with TPE‐OLD using a siRNA approach. In agreement with earlier findings (Figure 2D), we focused on TRF2, RBPJ, and TZAP. Furthermore, we added CTCF due to its global role in chromatin organization and SMCHD1, an important chromatin‐associated factor found at telomeres in separate studies (Grolimund et al. 2013; Kan et al. 2017; Vančevska et al. 2020). Validation of our siRNAs showed specific downregulations across conditions (Figure S9). However, siRNAs targeting *TERF2* also significantly decreased *RBPJ* (*p =* 0.0306, Holm‐Sidak's multiple comparisons test). As expected, no changes were observed in our control condition (pCMVScble; Figure S10). Strikingly, independent of the WE‐related motif tested (WE, *GLIS2*), we observed a significant decrease in eGFP‐positive cells upon either *TERF2*, *SMCHD1*, or *RBPJ* knockdown (Figures 4B–D and S11–S13). Because si*TERF2* conditions also decreased *RBPJ*, one could hypothesize that effects observed in si*TERF2* are imputable to the off‐target effect inducing *RBPJ* downregulation, potentially caused by their cross‐talk (Bottoni et al. 2019). Concurring, changes were less pronounced in the si*TERF2* than in the si*RBPJ* conditions across constructs (Figure 4B–D). In agreement with a role of SMCHD1 in chromatin variegated effect at telomeres (Laberthonnière et al. 2023; Tardat and Déjardin 2018; Vančevska et al. 2020), we report a lower proportion of eGFP‐positive cells upon *SMCHD1* downregulation, independent of constructs, and motifs (WE, *GLIS2*).

Taken together, our observations confirmed the insulator/enhancer properties of WE (and associated sequences) and support RBPJ and SMCHD1 as putative *trans* effectors. On the other hand, the TPE‐OLD mechanism appears independent of TZAP or CTCF and only weakly associated with TRF2 for the different sequences tested.

### 3.5 WE‐Associated Motifs Correspond to Young Alu Elements

**FIGURE 5:**
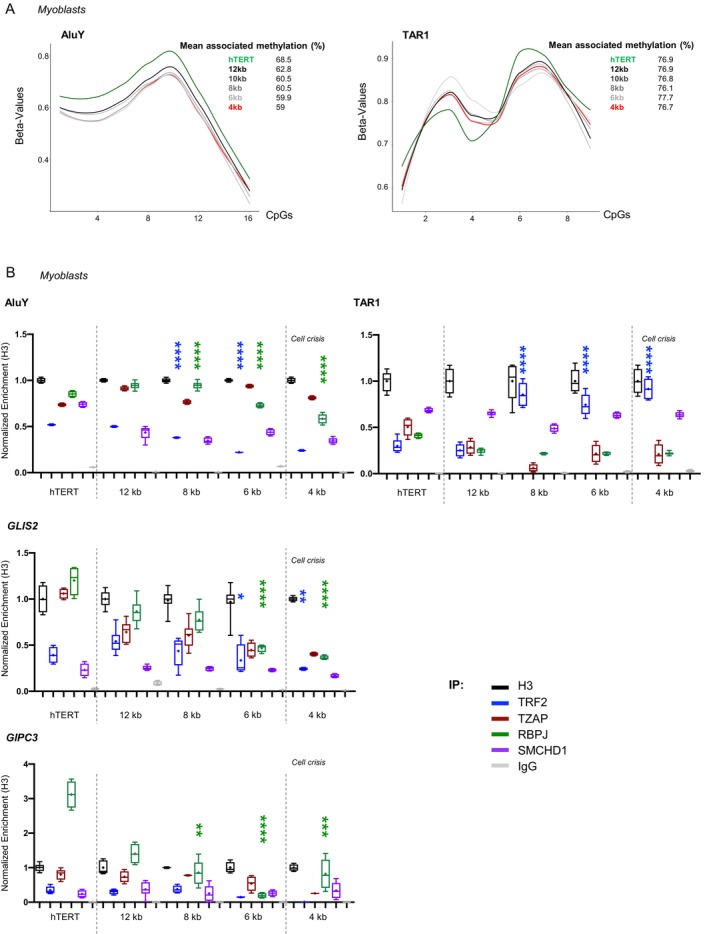
Associated TPE‐OLD motif is regulated by RBPJ enrichment at specific loci in vitro. (A) Smoothed mean methylation per CpG obtained in myoblasts with different telomere length after high‐throughput targeted bisulfite sequencing of either Alu*Y* or TAR1 repeats corresponding to amplicons of 219 bp with 16 CpGs and 150 bp with 9 CpGs, respectively. We report the methylation beta_value for all CpGs in the sequence of interest. *A* value of 1 corresponds to a fully methylated CpG; 0 to unmethylated. (B) Protein enrichment at Alu*Y*, TAR1, *GLIS2*, and *GIPC3* loci detected by ddPCR after ChIP in isogenic myoblasts with various telomere lengths. We report the enrichment of H3 (black), TRF2 (blue), TZAP (maroon), RBPJ (green), SMCHD1 (purple), and IgG (gray) normalized to H3. Dashed gray lines represent the external limit of telomere length (active telomerase, 4 kb; respectively). ChIP‐ddPCR was performed in biological quadruplicates. Holm‐Sidak's multiple comparison test; *α* = 0.05. p* < 0.05; p** < 0.005; p*** < 0.001; p**** < 0.0001.

We noticed that WE motifs found in the vicinity of TPE‐OLD genes shared homology with Alu repeats. Indeed, the WE element is present within Alu, as identified by RepeatMasker. Using our transcriptomic and methylome data (Figure 1B,C), we observed that Alu*Y* elements (Figure S14) are enriched in the proximity of DEGs (*χ* ^2^ test; Figure S15a). Alu*Y* belongs to the youngest subfamily of Alu repetitive elements. This subclass is highly methylated in somatic cells and is defined by a higher rate of genetic mobility when compared to others (Mei et al. 2011; Weisenberger et al. 2005). Hence, we asked if DNA methylation of this particular repeat was modulated in our isogenic clones. We recorded a constant trend towards hypomethylation throughout our panel of cells with decreasing telomere lengths (i.e., 12–4 kb). This trend is restricted to Alu*Y* elements, as it is not observed for the DNA methylation profile of TAR1, the main known subtelomeric repeat (Young et al. 2020) (Figures 5A and S15b).

### 3.6 TPE‐OLD Is Regulated by RBPJ but Not Dependent on TRF2 or TZAP Enrichment

We then hypothesized that robust TPE‐OLD *trans* partners should present a gradual depletion at the WE‐related motif in association with shorter telomere length. To further confront the results obtained using reporter assays to the respective in situ genetic and epigenetic context, we evaluated protein enrichments at the same selected loci (*GLIS2*, *GIPC3*, *BET1L*, *C16Orf74*) and different repeats (Alu*Y*, TAR1) in isogenic myoblasts with controlled telomere length, including in cells with active telomerase and cells reaching crisis (positive to DNA damages; average telomere length: 4 kb). We performed ChIP‐ddPCR experiments after immunoprecipitation using either anti‐H3, ‐TRF2, ‐TZAP, ‐RBPJ, or ‐SMCHD1 antibodies (Figures 2 and 4).

We found a significant decrease in both RBPJ and TRF2 enrichment at Alu‐associated loci (Figure 5B; *p* < 0.0001 and *p* < 0.0001; respectively, Tukey's multiple comparisons test). In the context of TAR1‐associated loci, we showed an enrichment of TRF2 in cells with shorter telomeres (8–4 kb, *p* < 0.0001; Tukey's multiple comparisons test). This suggests a relocation of TRF2 at subtelomeres upon telomere shortening, as reported by others (Mukherjee et al. 2018). At *GLIS2*, we noticed a constant trend towards a depletion of RBPJ (*p* < 0.0001; Tukey's multiple comparisons test) whereas enrichment in other proteins remained unchanged. Moreover, we note a global enrichment in RBPJ and, to a lesser extent, TZAP, in cells with active telomerase (labeled hTERT). Similar observations were made at *GIPC3*, *BET1L*, and *C16orf74* loci (Figures 5B and S16). These results highly suggest that RBPJ acts as a *trans* partner of TPE‐OLD and confirm a role for TRF2 depending on the chromatin context.

### 3.7 TPE‐OLD Chromatin Loop Is Modulated by RBPJ

Next, we analyzed the 3D conformation of *GLIS2* at the 16p subtelomeric loci (Figure S17a,b). As expected, the TPE‐OLD gene was found in close proximity to telomeres in cells with long telomeres (12 kb), but further apart in cells with shorter telomeres (8 kb). Importantly, the 3D conformation changes were restricted to the *GLIS2* locus and were not observed at the *PARN* locus (Figure S17).

**FIGURE 6:**
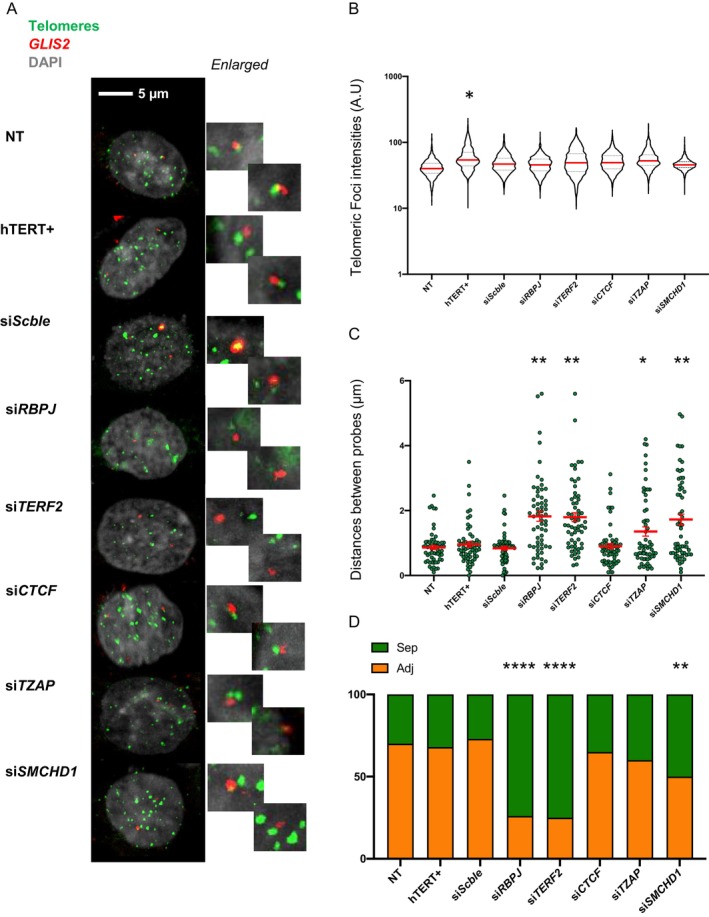
RBPJ and TERF2 modulate the topological organization of TPE‐OLD genes. (A) Confocal images of 3D DNA‐FISH targeting *GLIS2* (red) and telomeres (green) in myoblasts with long telomeres under various conditions. Briefly, 30 pictures of nuclei were taken per condition either 72 h after siRNA treatments or after complete selection (hTERT+). The hTERT+ sample corresponds to the overexpression of telomerase in cells with formerly shorter telomeres (6 kb). Each *Z*‐stack picture was then reconstructed using the IMARIS software and analyzed. (B) Quantification of telomeric intensities (*n* > 1700) along with (C) distances between the closest telomeric signal and probe (*n* = 60 alleles). Dunnett's T3 multiple comparison test; α = 0.05. p* < 0.05; p** < 0.005; p**** < 0.0001. (D) Summary of events and associated comparison test to control (NT). The association between *GLIS2* and its closest telomere is lost upon downregulation of either *RBPJ* or *TERF2* and modestly upon *SMCHD1* downregulation. *χ* ^2^ test; *α* = 0.05. p** < 0.005; p**** < 0.0001.

Exploiting the direct visualization of this TPE‐OLD loop in myoblasts, we then asked if the loop was reversible upon telomerase reactivation in cells with shorter telomeres (labeled hTERT+; Figure 6). Second, using candidates tested previously either in HEK 293 (siRNA, Figure 4) or myoblasts with controlled telomere length (ChIP‐ddPCR, Figure 5), we investigated if the TPE‐OLD loop was dynamic upon downregulation of its *trans*‐partners candidates: RBPJ, TRF2, CTCF, TZAP, and SMCHD1 (Figure S18).

Upon telomere re‐elongation, the loop was restored, supporting previous findings showing that TPE‐OLD loops are reversible and dependent on telomere length (Robin et al. 2014). Besides, downregulation of *RBPJ* or *TERF2* modulated the loop by increasing the distances between *GLIS2* and its closest telomere. The same observation, but at a more moderate level, was made upon *SMCHD1* downregulation (Figure 6). No differences between telomere lengths were observed across tested conditions, with the exception of hTERT+ conditions. Coincidentally, *GLIS2* was upregulated (*p* = 0.0481; Holm Sidak's multiple comparisons test) upon *RBPJ* downregulation, as other confirmed TPE‐OLD genes (Figure S18c). Of note, unlike others (*C1S*, *ISG15*), *GLIS2* upregulation was restricted to *RBPJ* downregulation, suggesting a hierarchical sensitivity of TPE‐OLD genes. These findings showing an impact of RBPJ and SMCHD1 on TPE‐OLD are in line with our previous results on reporter gene expression in HEK 293 (Figure 4C).

Overall, our results described a dynamic enrichment at TPE‐OLD loci of RBPJ proportional to telomere length, whereas other factors (TZAP, SMCHD1) remained stable, with the exception of TRF2 that, in specific contexts (TAR1, *BET1L*), are enriched in cells with short telomeres. This observation might be imputed to the close proximity of the loci to telomeres (2–4 kb, 200 kb; respectively) (Mukherjee et al. 2018). Interestingly, RBPJ appears as the unique robust *trans* partner among candidates investigated across cell types and multiple molecular strategy (OMICs, siRNA, and 3D FISH).

Taking together our results detailed for the first time the molecular basis of TPE‐OLD through a common *cis* motif that can be directly impacted by RBPJ. Briefly, we propose that TPE‐OLD is multifactorial and relies on the association of long telomeres, a common motif (affected by DNA methylation) and associated proteins allowing the creation of the chromatin loop. Our work identifies the motif and pinpoint proteins involved in this newly described chromatin structure.

## 4 Discussion

In this study, we explored the mechanisms related to TPE‐OLD (Jäger et al. 2022; Dong et al. 2021; Robin et al. 2014), a progressive phenomenon linked to telomere shortening and likely associated with aging (López‐Otín et al. 2013). Aging‐related changes in gene expression include modifications of epigenetics marks and chromatin structure (Horvath 2013; Bell et al. 2019), both processes associated with TPE‐OLD lacking further investigations (Kim et al. 2016; Robin et al. 2014, 2020). Here, we delineate novel TPE‐OLD key molecular features. According to our findings, TPE‐OLD involves (i) a *cis* element, (ii) telomere‐associated proteins, and (iii) DNA methylation. This suggests a coordinated mechanism associated with several aging hallmarks (e.g., telomere shortening, DNA methylation).

Interestingly, we observed that the *cis* element we identified is highly similar to Alu elements and more precisely Alu*Y*, the most recent subfamily of Alu repeats (Wildschutte et al. 2015). The repartition of Alu in the genome is highly correlated with the CG content, gene density, and enriched in intragenic regions (Grover et al. 2004). Consistent with our findings on the 3D conformation of TPE‐OLD loops, Alu elements can form distant genomic interactions (Ferrari et al. 2020) and modulate transcription by acting as a *cis* element for transcription factors (Su et al. 2014). If recent studies report their enhancer activities (Gorbunova et al. 2021; Su et al. 2014), Alu elements are also able to induce either up‐ or downregulation of associated genes (Payer et al. 2021); a consideration reminiscent of TPE‐OLD (Robin et al. 2014; Jäger et al. 2022; Dong et al. 2021). In agreement with changes in DNA methylation in isogenic clones with long and short telomeres, Alu methylation level decreases with age (Jintaridth and Mutirangura 2010) and in age‐associated pathological contexts such as Alzheimer (Bollati et al. 2011) (where telomere shortening has been observed). This further supports our hypothesis of a functional link between telomere length, TPE‐OLD, DNA methylation, and Alu elements. Enhancer‐like characteristics of Alu are shown to follow an evolutionary continuum (Su et al. 2014), with “finalized” proto‐enhancer corresponding to the most recently evolved Alu subclass: Alu*Y*. Our findings on the biological function of the WE motif are in line with these hypotheses.

Beyond, we uncovered proteins that are associated with TPE‐OLD. As shown by our siRNA approach, TPE‐OLD *trans* partners can modulate its effect (e.g., TRF2, RBPJ and SMCHD1). However, their involvement is restrained to their associated genomic context. TRF2 can act as a potential modifier of the *cis* element in vitro but is not observed in vivo in the genomic context tested (*GLIS2*, Alu, TAR1) as we do not report any depletion of TRF2 at these loci. On the contrary, we showed an enrichment of TRF2 at TAR1, concomitant to telomere shortening. TRF2 is relocated towards the most distal telomeric sequences as the telomere shortens, as suggested by others (Mukherjee et al. 2018) without modification of its global protein level (Figure S19). Likewise, regulation by SMCHD1 is possible but appears to be context‐dependent. Indeed, from our findings SMCHD1 act as a modifier of the *cis* element (Figure 4B–D), is not depleted either at the protein level (Figure S19) or at tested loci (Figure 5) but can act as a chromatin modulator (Figure 6). Hence, this suggests a key role for SMCHD1 as a chromatin organizer at the telomere and echoes previous work deciphering its role in human pathologies (Dion et al. 2019; Vančevska et al. 2020; Laberthonnière et al. 2021).

Strikingly, the most consistent *trans* partner of TPE‐OLD (i.e., acting on WE element and depleted at TPE‐OLD loci upon telomere shortening), RBPJ, is a transcriptional factor that triggers the recruitment of chromatin remodeling complexes, including histone modifiers (Castel et al. 2013); with a possible dynamic interaction with enhancers (Wang et al. 2014). In our hands, where RBPJ protein level remained unchanged upon telomere shortening (Figure S19), the WE motif could be this potential common enhancer element involved in genome‐wide long‐distance interactions upon RBPJ repositioning. Our work showed a direct interaction of RBPJ with WE. However, future studies will determine if RBPJ unambiguously interacts with telomeres or through other intermediates (e.g., TERRA, proteins).

Of note, previous studies did not consider individual telomere lengths but only overall average telomere lengths (Laberthonnière et al. 2019). This simplification does not integrate the well‐accepted notion that human telomere ends (e.g., 92 chromosome ends) are highly variable from one chromosome to another and between respective arms for each individual (Kappei and Londoño‐Vallejo 2008; Laberthonnière et al. 2019; Karimian et al. 2024). Using our system, we minimized telomere variations by performing experiments in a homogenous genetic background (isogenic clones). Therefore, our work is not without limitations. Our cellular models are similar to the one used to illustrate TPE‐OLD (Robin et al. 2014) and the cells used to investigate the insulator/enhancer properties of the motifs are HEK (widely used for transfection); these models remain somehow artificial. To strengthen our findings on TPE‐OLD associated proteins from the HEK assays (Figure 4), we challenged our observations using myoblasts and complementary techniques (3D DNA‐FISH). We reached identical conclusions by analyzing the TPE‐OLD associated DNA loops (Figure 6). Nevertheless, future directions to illustrate the impact of TPE‐OLD in physiological (aging) and pathological contexts will require a different approach, based on primary cells from relevant tissues and/or the extensive use of large cohorts (> 40 individuals to overcome heterogeneity) to encounter for the multifactorial aspect of TPE‐OLD raised by our study. The singular telomere length, exact motif sequence, protein levels, and potential mutations (e.g., RBPJ, TRF2, SMCHD1) should be assessed. This technical challenge will benefit from the advances provided by extra‐long read sequencing technologies (e.g., Oxford nanopore technology).

In line with this remark, a recent study focusing on TPE‐OLD used positional conservations across selected species that relied on replicative senescence as an aging mechanism to discriminate roughly 2000 potential TPE‐OLD genes. Our complementary work highlighted the same ratio of TPE‐OLD genes with an overlap of 10% between data sets (Figure S20, Table S2) including *PPP2R2C* and *GLIS2*. This restricted list could represent the “core” TPE‐OLD genes, whereas remaining candidates might represent tissue‐specific TPE‐OLD candidates (Jäger et al. 2022; Laberthonnière et al. 2019).

Furthermore, profiting from the extensive dataset from the Gene Tissue Expression (GTEx) project (Demanelis et al. 2020) associating gene expression, telomere length, and tissue specificity, we confronted our results from myoblasts and myotubes (DEGs) along with the latest TPE‐OLD gene list available (Jäger et al. 2022) (Figure S20; Table S2). In this cross‐study comparison, we report a 14.5% overlap between our datasets and the genes shown to be modulated in aged skeletal muscle (14.54% with myoblasts, 14.55% with myotubes; respectively) as opposed to a 4.5% overlap when comparing the list generated by Jäger and colleagues with the corresponding dataset (i.e., unexposed skin). If promising, these observations need to be confirmed in future studies using a larger cohort where one can assess lifestyles, among other factors.

All things considered, one could hypothesize that the combination of telomere length heterogeneity coupled with WE‐polymorphisms (Alu*Y*‐like repeats) offers an endless combination of systems where TPE‐OLD associated genes are affected upon stimuli either by telomere shortening (progressive) or WE‐polymorphisms (static). Our current work mostly described upregulation. However, TPE‐OLD has been shown to modulate gene expression and splicing in restricted contexts (Robin et al. 2015). Whether proximal genes are up or downregulated depends on the complete chromatin context that includes chromatin structures (DNA looping) but also protein accessibility such as transcription factors and/or DNA methyltransferases, among others. This multifactorial context could explain the enrichment of deregulation reported by others at subtelomeres (mostly up regulation) (Dong et al. 2021; Jäger et al. 2022). In large panels, the statistical chances of combining telomere shortening and WE‐polymorphisms, allowing genes to be freed from TPE‐OLD, are more frequent at these specific loci.

Taken together, our work indicates that telomeres and TPE‐OLD impact have been largely overlooked in genome regulation as well as in diseases such as age‐related pathologies where telomere might act as a susceptibility factor. This could also hold true for a number of diseases linked to subtelomeric imbalance where the identification of TPE‐OLD sensitive genes might help in solving the missing heritability or explain incomplete penetrance and phenotypical variability. In agreement with recent studies (Jäger et al. 2022; Projahn et al. 2024), our findings advocate for a *quasi*‐programmed aging concept. The TPE‐OLD mechanism suggests a highly regulated and adaptative process, mitigating the consequence of aging hallmarks including pitfalls of replicative stress that are driven by growth and development. If provocative, this argument hints toward developmental and aging determinism dictated by individual subtelomeric contexts. Our descriptive work combining in vitro and in silico approaches opens innovative perspectives of research by stressing the importance of TPE‐OLD and its implications in human patho‐physiology to evaluate disease mechanisms, risk factors, and comorbidities associated with aging.

## Author Contributions

Conceptualization: J.D.R. Methodology and material support: R.C., V.M.P., N.J., L.C., P.V.P., F.M., and J.D.R. Visualization: R.C., V.M.P., L.C., P.V.P., and J.D.R. Supervision: J.D.R. Writing original draft: J.D.R., R.C., and V.M.P. Writing review and editing: R.C., V.M.P., N.J., L.C., P.V.P., F.M., and J.D.R.

## Conflicts of Interest

The authors declare no conflicts of interest.

## Supporting information


Figures S1–S20.

**Table S1.** Conserved methylation DMRs across cell types.

**Table S2.** Comparison list across datasets.

Data S1. Associated statistical analysis

Data S2. HiC pipeline and Primer list

## Data Availability

The data underlying this article are available in the article and in its online Supporting Information. RNASeq and EpicArray from this work are accessible through the GEO portal (https://www.ncbi.nlm.nih.gov/gds), under the access codes GSE213281 and GSE213427, respectively. The datasets derived from sources in the public domain are accessible from ReMap (https://remap2022.univ‐amu.fr/) or GEO (https://www.ncbi.nlm.nih.gov/gds), under the accession numbers: TRF1 and TRF2, GSE26005; TZAP, GSE96778; HOT1, GSE46237; RBPJ, GSE29498; HiC data, GSE163666.
