# Supplementary material for: Telomere Position Effect‐Over Long Distances Acts as a Genome‐Wide Epigenetic Regulator Through a Common Alu Element
Source: Aging Cell. 2025 Mar 10;24(6):e70027. doi: 10.1111/acel.70027 (PMC12151916; doi:10.1111/acel.70027)
Supplement: Supplementary file 1 — Figures S1–S20. [file ACEL-24-e70027-s003.pdf]

Supplemental Figure 1.

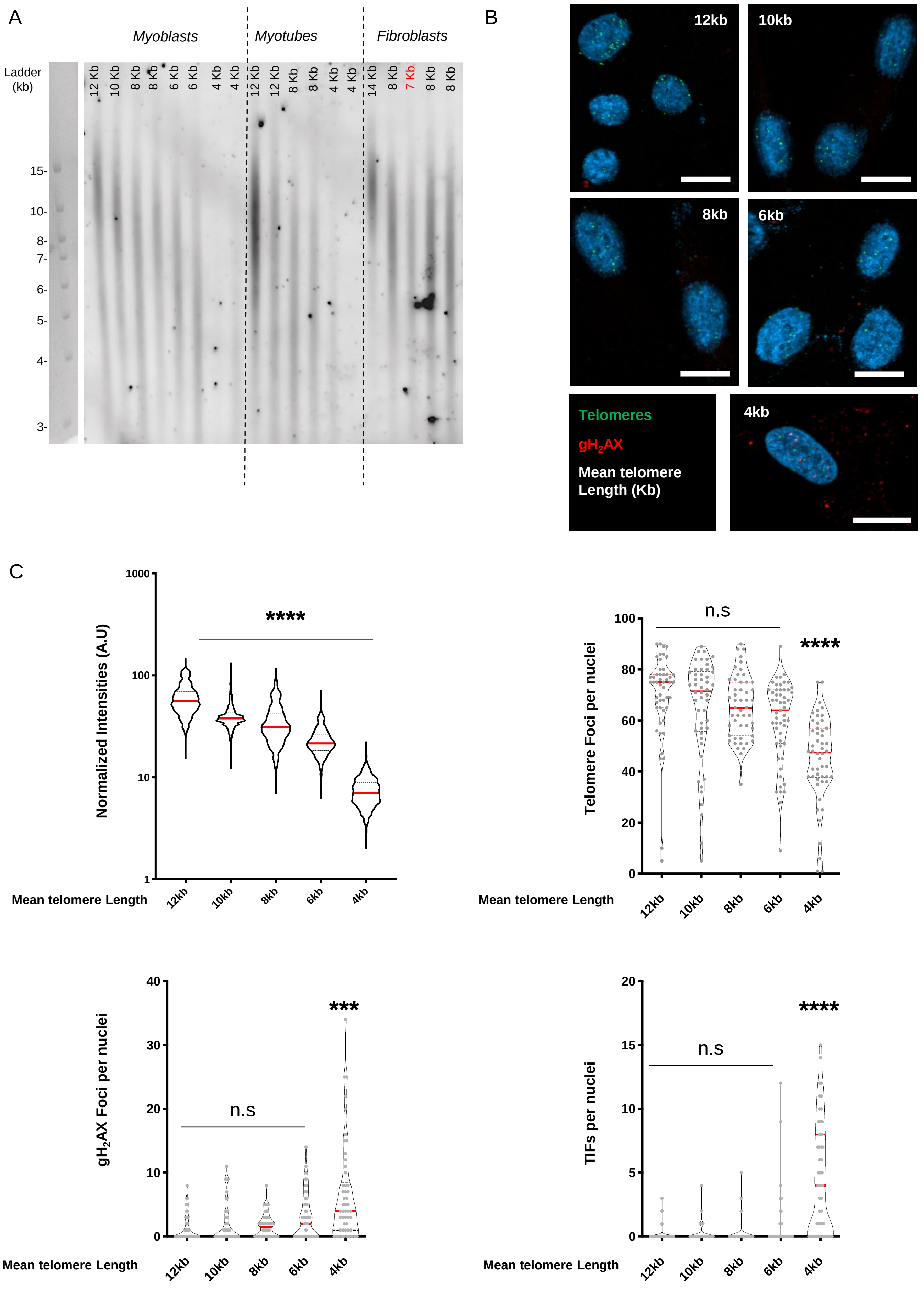

Supplemental Figure 1.

A. Representative Telomere Restriction Fragment analysis (TRF) of the cellular models used for the study as described in Stadler *et al.* 2013 and Robin *et al.* 2014 (*1*, *2*). Myoblasts and their corresponding myotubes (post-differentiation) and fibroblasts were analyzed before detection of DNA damage signals **B.** Representative images of gH2AX and telomeric staining. Briefly Isogenic myoblast clones (described in Figure1) with various telomere lengths were fixed, hybridized with a telomeric probe (C-Rich) and further stained using a gH2AX antibody. A scale of 10 μm is reported by a white bar in each frame **C.** Associated quantifications. We separately report the normalized mean intensities of telomeres; the number of telomeric and gH2AX foci along with the colocalized events (Telomeric Induced Foci, TIFs) per nuclei in each isogenic clone (n=50 per quantification). Medians, quartiles and all data points are shown in violin plots. Holm-Sidak's multiple comparison test; $\alpha = 0.05$. *p* *** <0,001; *p* **** <0,0001.

Supplemental Figure 2.

A

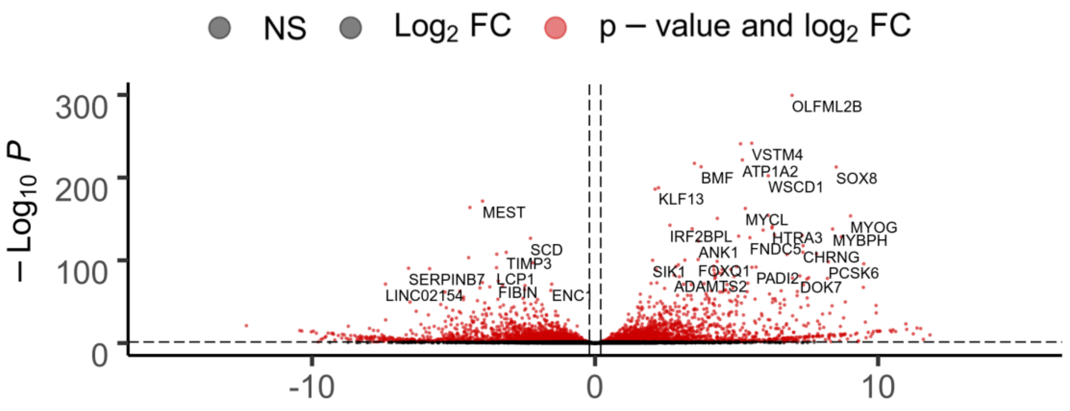

Log2FC < 0 = Myoblast Tel 8kb downregulated
Log2FC > 0 = Myoblast Tel 8kb upregulated

B Upregulated in Myoblast 8kb (*vs* 12kb)

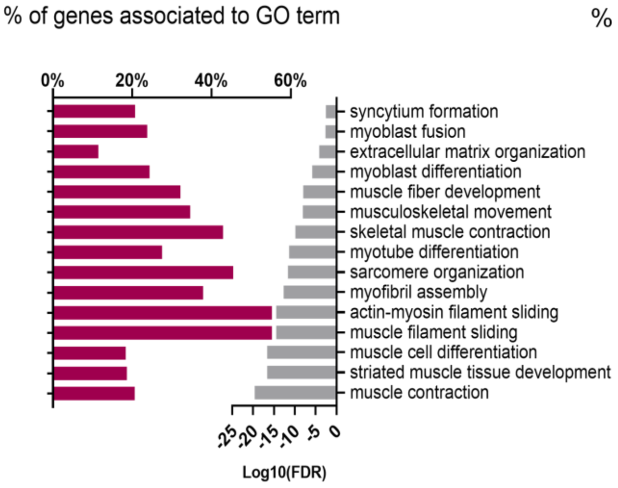

C Downregulated in Myoblast 8kb (*vs* 12kb)

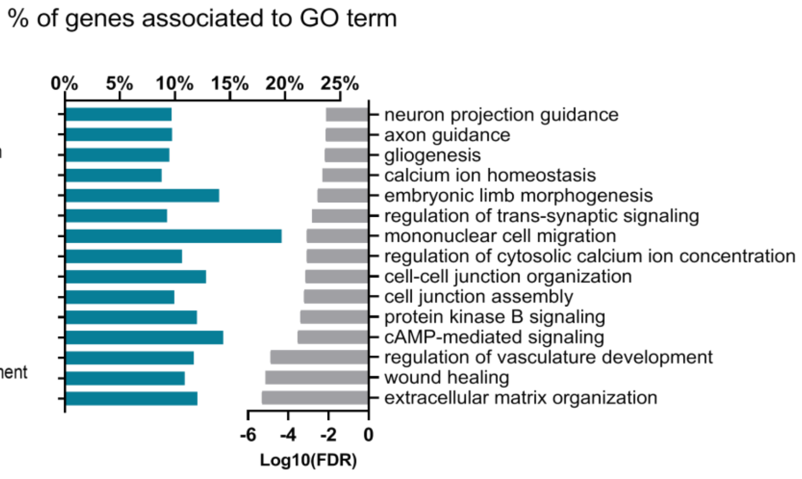

D

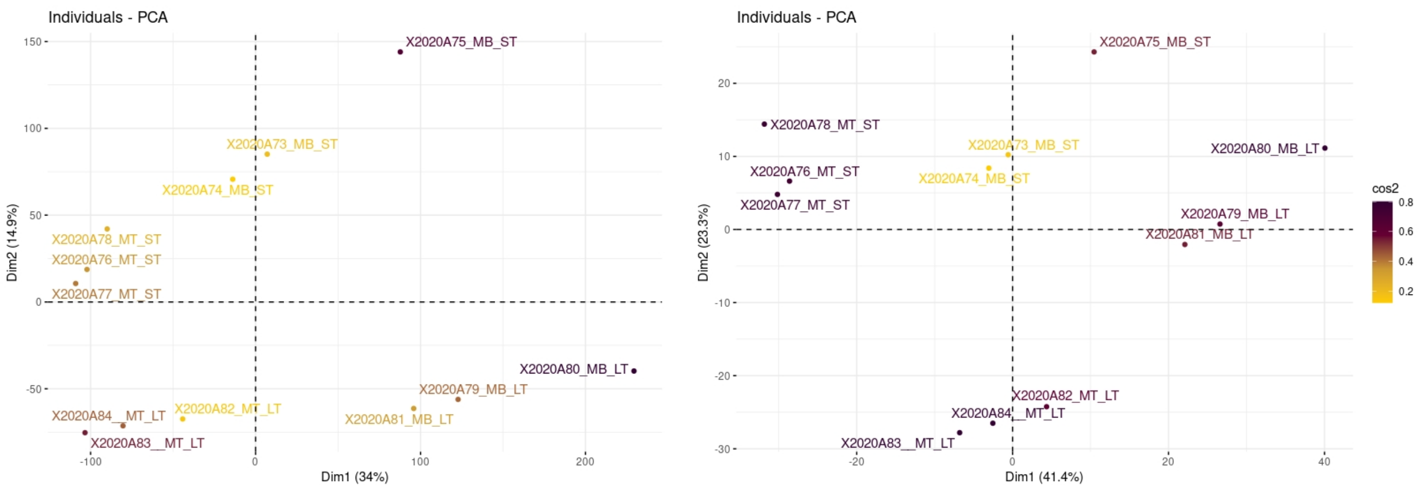

Supplemental Figure 2.
A. Volcano plots associated with transcriptomic analysis (RNASeq) for differential expression comparing isogenic clones of myoblasts with long (12 Kb) and shorter (8 Kb) telomeres. Log2(FC) and -log10 (FDR) are plotted on the *x*- and *y*-axis, respectively. Black dots represent genes that did not reach the significance thresholds whereas differentially expressed genes are shown in red. **B-C.** Gene Ontology (GO) for Biological pathways (BP) corresponding to enrichment analysis of upregulated (**B**) of downregulated (**C**) DEGs in myoblasts with short telomeres (8 Kb) *vs.* myoblasts with long telomeres (12 Kb) filtered on -2>FC>2 and FDR <0.05. Bar plots in the left (dark red/blue; respectively) represent the percentage DEG out of the total genes of associated with a GO-term shown in the right column. Grey bars in the right represent (Log10 of False Discovery Rate) for each BP. **D**. Principal Component Analysis (PCA) of Myoblasts and Myotubes with either long or short telomeres. We report the PCA using the complete transcriptomic data associated to myoblasts and myotubes (left) along with a PCA using a subset of gene corresponding to all genes tagged with a skeletal muscle component. In both PCA diagram we observed that the myoblasts with short telomeres (MB-ST) are closer to their myotubes counterpart (MT) than the myoblasts with long telomeres (MB-LT). We also note one outlier in our myoblasts with short telomeres triplicate (Fig. 1B) of our manuscript. To obtain the myogenic genes, we filtered out 1371 genes with a descriptor 'muscle' from gene ontology database (http://amigo.geneontology.org/amigo/search/bioentity?q=muscles). The selected genes were cross referenced with the raw read counts for all the sequenced samples (RNAseq). The PCA plot was generated in R using libraries "factoextra" and "FactoMineR".

Supplemental Figure 3.

A

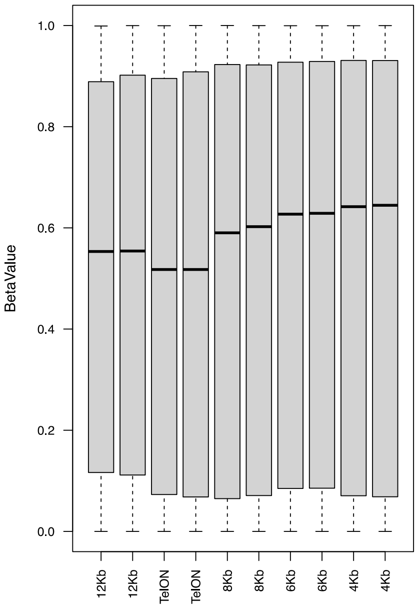

B

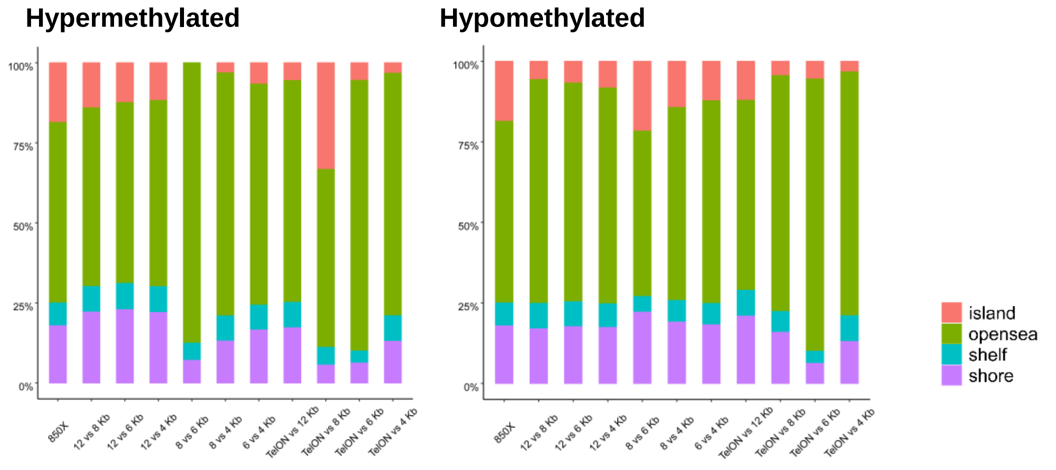

C

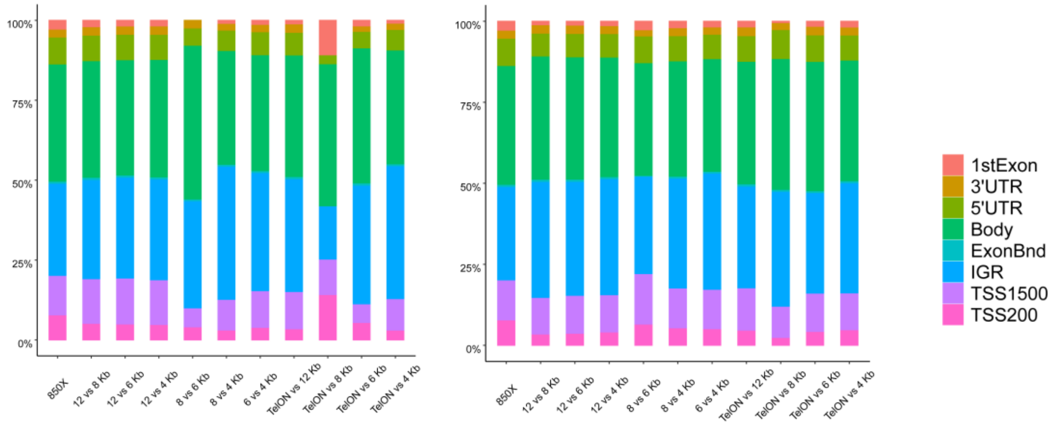

Supplemental Figure 3.

A. Boxplot reporting the median methylation from methylome array (EpicArray 850K) in myoblasts with different telomere lengths. TelON corresponds to myoblasts where the telomerase was not removed. **B.** Stacked barplots representing the distribution of Hypermethylated (left) and Hypomethylated (right) probes relative to CpG islands, shores (2 Kb flanking CpG islands), shelves (2 Kb extending from shores) or openseas (isolated CpG in the rest of the genome) in myoblasts with shorter telomere (8 Kb) compared to isogenic clones with long telomeres (12 Kb; padj < 0.05 and abs (deltaBeta) > 0.2). **C.** Stacked barplots representing the distribution of hypermethylated (left) and hypomethylated (right) probes corresponding to genes first exon; 3' UTR; 5'UTR; gene bodies; exon boundaries; internal genomic regions (IGR); probes located 1500 bp from transcription start sites (TSS1500) or 200 bp from transcription start sites (TSS200) in myoblasts with shorter telomeres (8 Kb) compared to isogenic clones with long telomeres (12 Kb; padj < 0.05 and abs (deltaBeta) > 0.2).

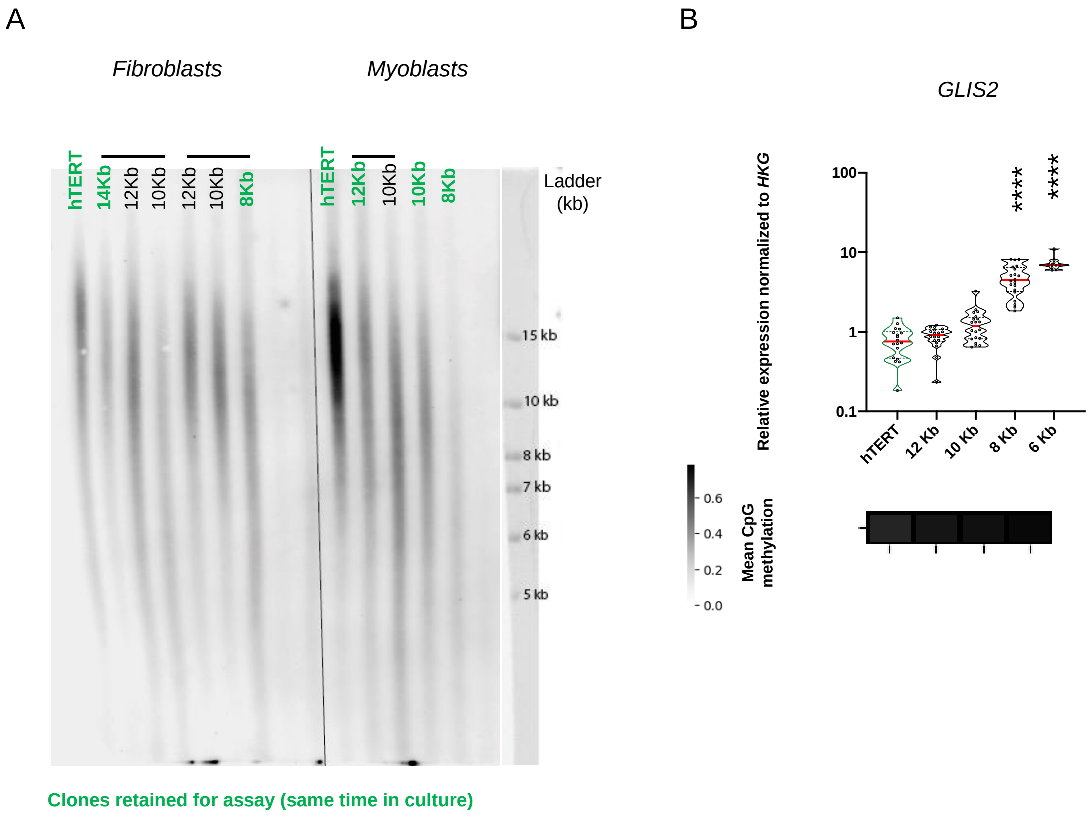

Supplemental Figure 4.

A. Representative Telomere Restriction Fragment analysis (TRF) of additional isogenic clones used for validation of the transcriptomic analysis (as presented in Fig. 1E). We report association of clones by a black bar (i.e., telomere length obtained by cell culture). Assay were performed using the clones whose telomere length are labeled in green. This limitation is set to respect the same time in culture parameter (*e.g.*, only telomere length differs). **B.** Associated expression of selected gene (RT-qPCR, *GLIS2*) in myoblasts and their mean CpG methylation. Results are normalized to house keeping genes (*HPRT*, *PPIA* and *GAPDH*) and respective expression in isogenic clones with long telomeres (12 Kb). For each condition, we report the average of three independent isogenic clones with technical duplicates. Medians, quartiles and all data points are shown in violin plots. Holm-Sidak's multiple comparison test; $\alpha = 0.05$. p **** <0,0001.

Supplemental Figure 5.

A

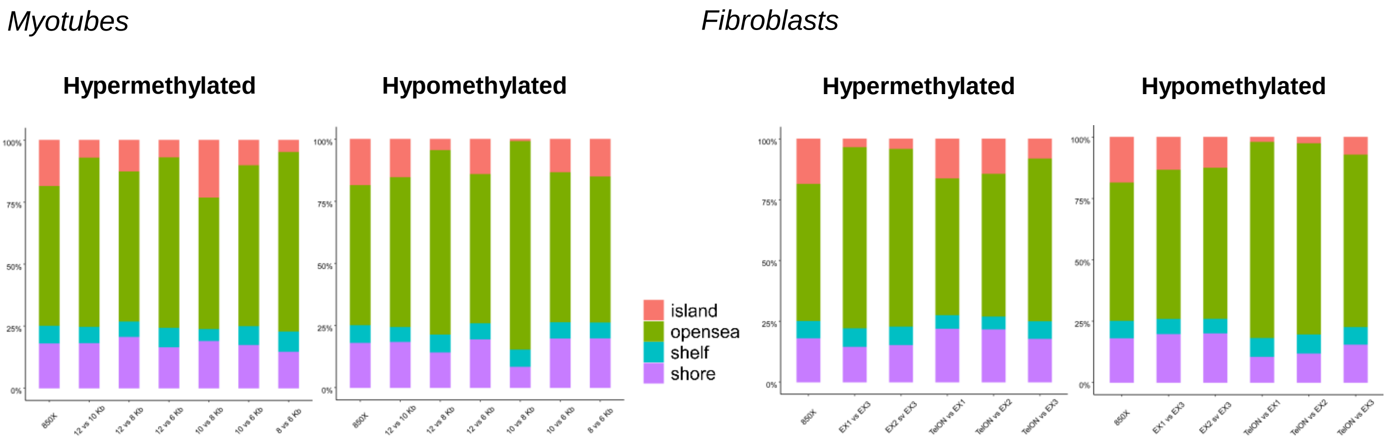

B

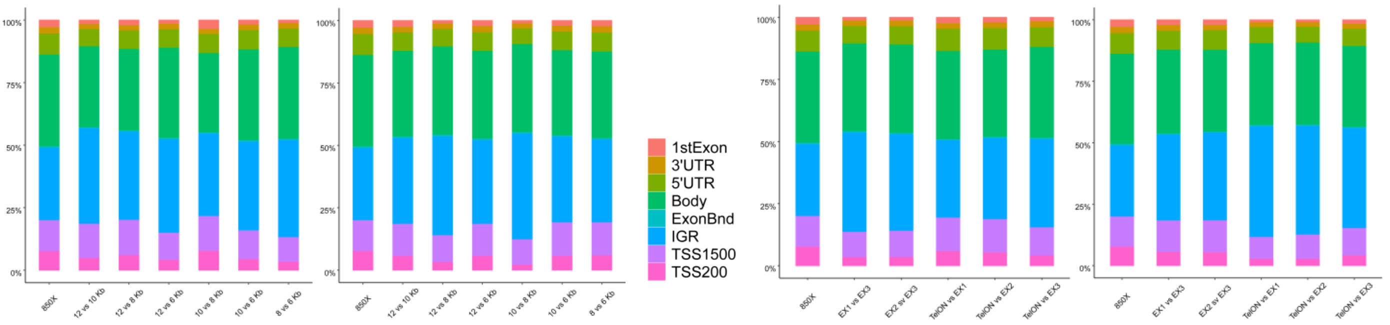

C

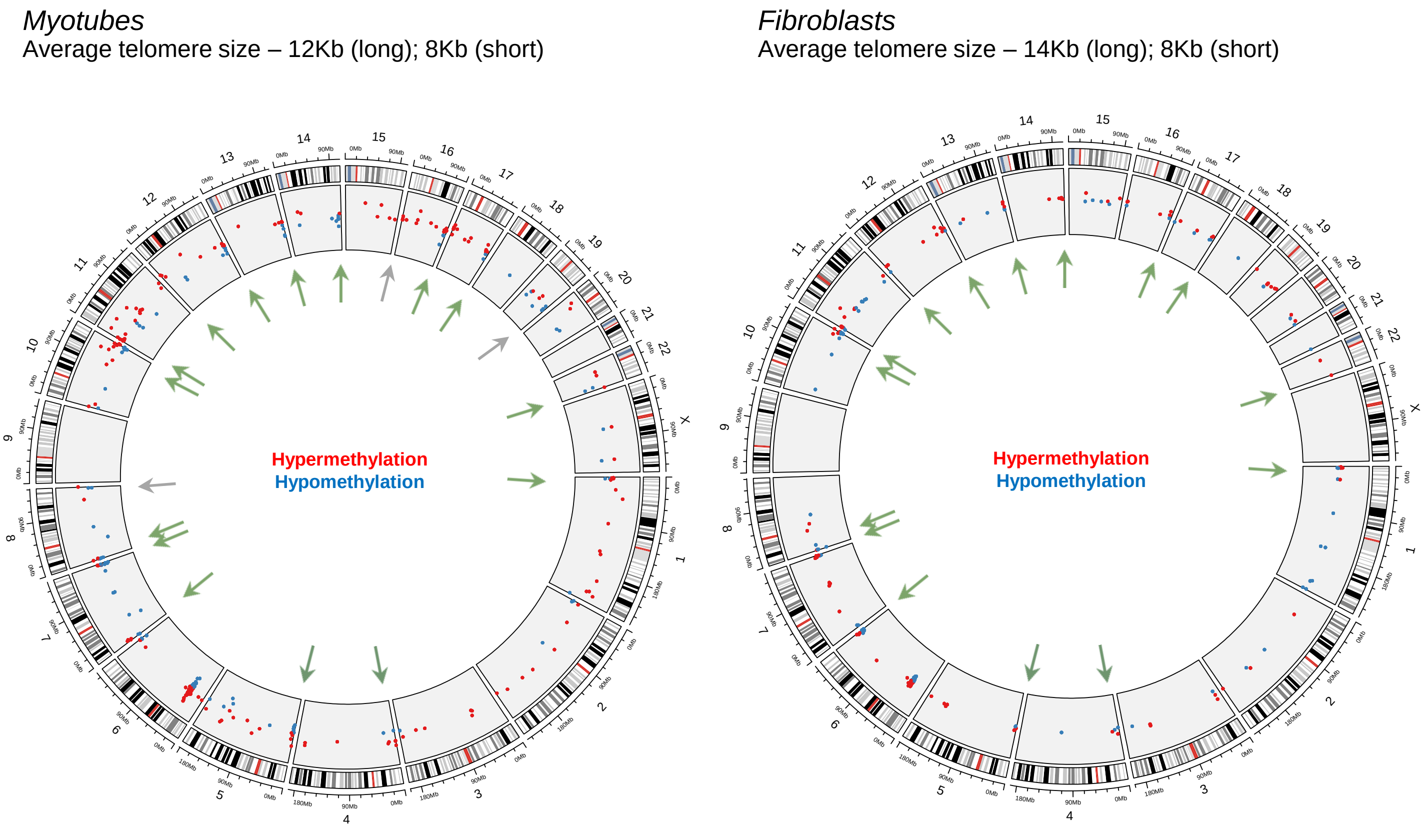

Supplemental Figure 5.

A. Stacked barplots representing the distribution of Hypermethylated and Hypomethylated probes relative to CpG islands, shores (2 Kb flanking CpG islands), shelves (2 Kb extending from shores) or openseas (isolated CpG in the rest of the genome) in either myotubes with shorter telomeres (8 Kb) compared to isogenic clones with long telomeres (left) or fibroblasts with shorter telomeres compared to isogenic clones with long telomeres (14 Kb, right). padj < 0.05 and abs (deltaBeta) > 0.2). **B.** Stacked barplots representing the distribution of hypermethylated and hypomethylated probes corresponding to genes first exon; 3' UTR; 5'UTR; gene bodies; exon boundaries; internal genomic regions (IGR); probes located 1500 bp from transcription start sites (TSS1500) or 200 bp from transcription start sites (TSS200) in either myotubes with shorter telomeres (8 Kb) compared to isogenic clones with long telomeres (left) or fibroblasts with shorter telomeres (8 Kb) compared to isogenic clones with long telomeres (11 Kb, right). padj < 0.05 and abs (deltaBeta) > 0.2). **C.** Distribution of differentially methylated regions (DMRs) across the genome in either isogenic myotubes clones with long (12 Kb) and shorter telomeres (8 Kb) or isogenic fibroblasts clones with long and shorter telomeres (left, right; respectively). Arrows (grey) point to DMRs located at subtelomeres, Arrows (green) point to common DMRs found in Myoblasts (Fig.1C), Myotubes and Fibroblasts.

Supplemental Figure 6.

A

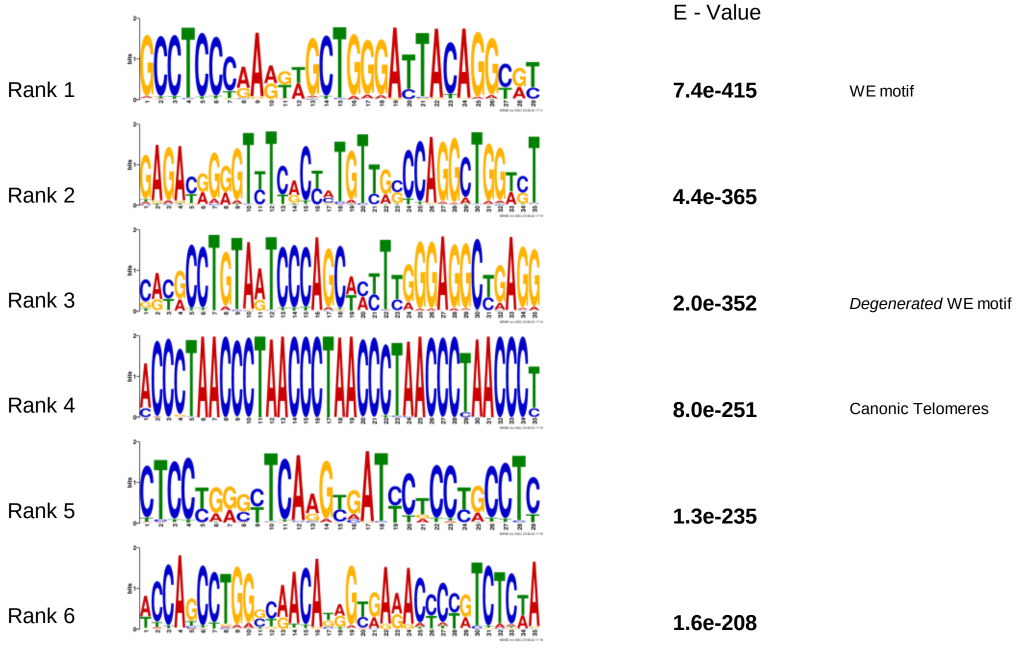

B

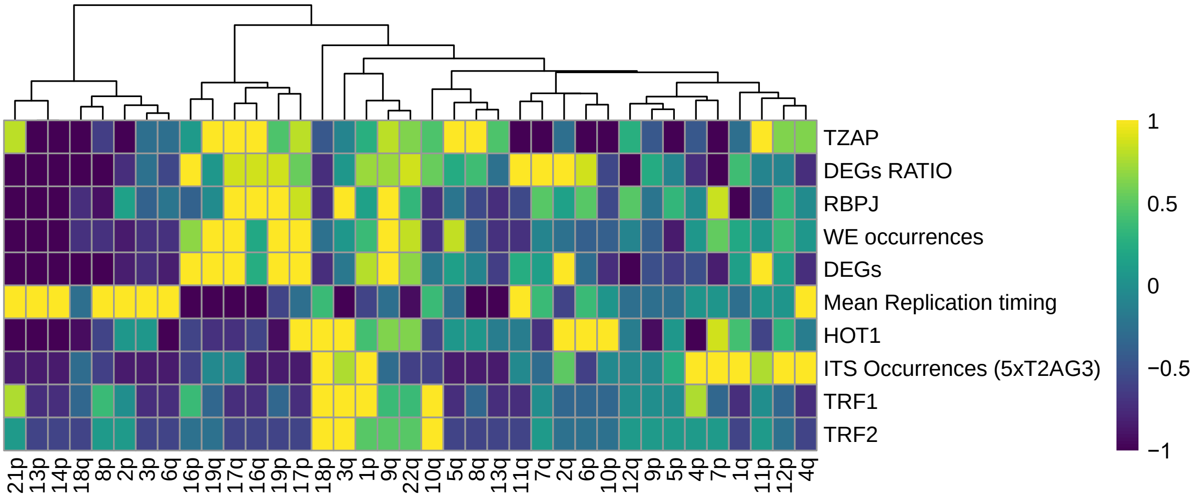

C

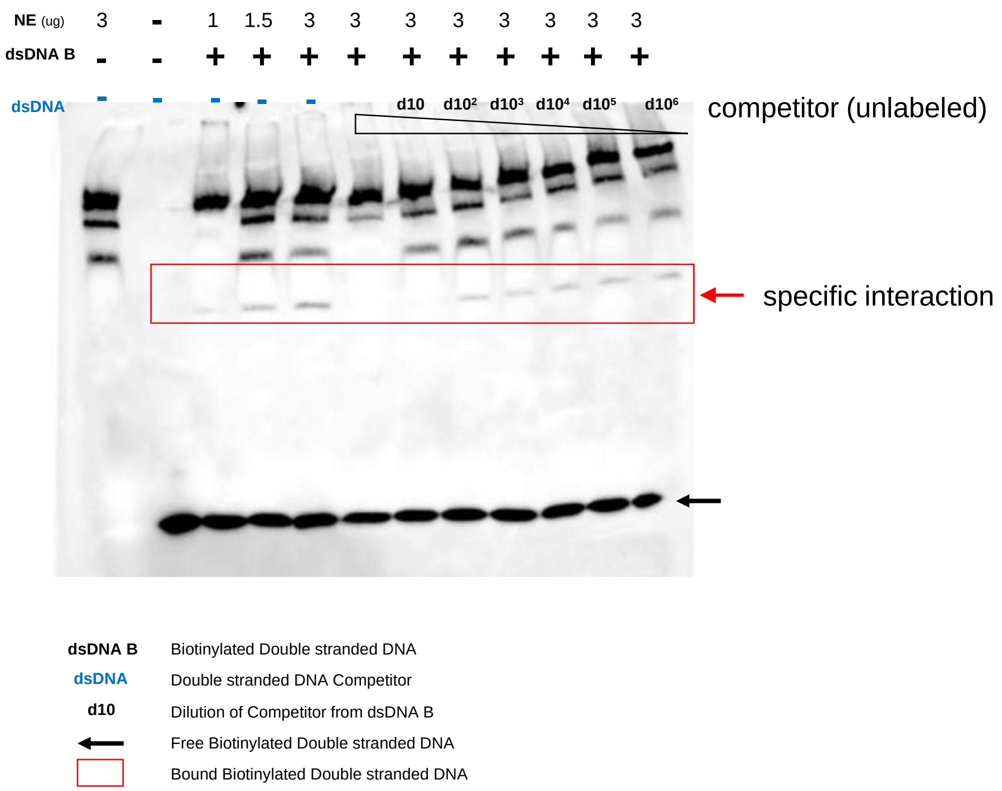

Supplemental Figure 6.

A. Motif logos of the top sequence present in direct proximity of telomere associated interactions identified from data generated in HiC using cells with long telomeres (i.e., immortalized fibroblasts). For each motif, we report the associated E-value. **B.** Unsupervised hierarchical clustering (WardD2; Manhattan distance) of subtelomeres (10Mb) with motif occurrences (WE), DEGs and telomeres associated factors (replication timing, proteins, Internal Telomeric Sequences ITS). Each parameter was extracted from available data set and normalized to a mean of 1. We report DEGs as total DEGs localized at each subtelomere and DEGs ratio when corrected for the gene density. **C.** Electrophoretic mobility shift assay (EMSA) results for WE 5’-CCTCCCAAAGTGCTGGGATTACAGGCGTGAGCCAC-3’ binding (dsDNA B). 5’ biotin labeled WE dsDNA was titrated with increasing amount of Nuclear Extract (NE) and unlabeled dsDNA competitor (blue) using serial dilutions. Biotinylated labeled dsDNA associated to a protein complex is shown in the red square; free unbound biotinylated dsDNA by a black arrow.

Supplemental Figure 7.

HEK- pCMV /pCMVTelo with Motifs

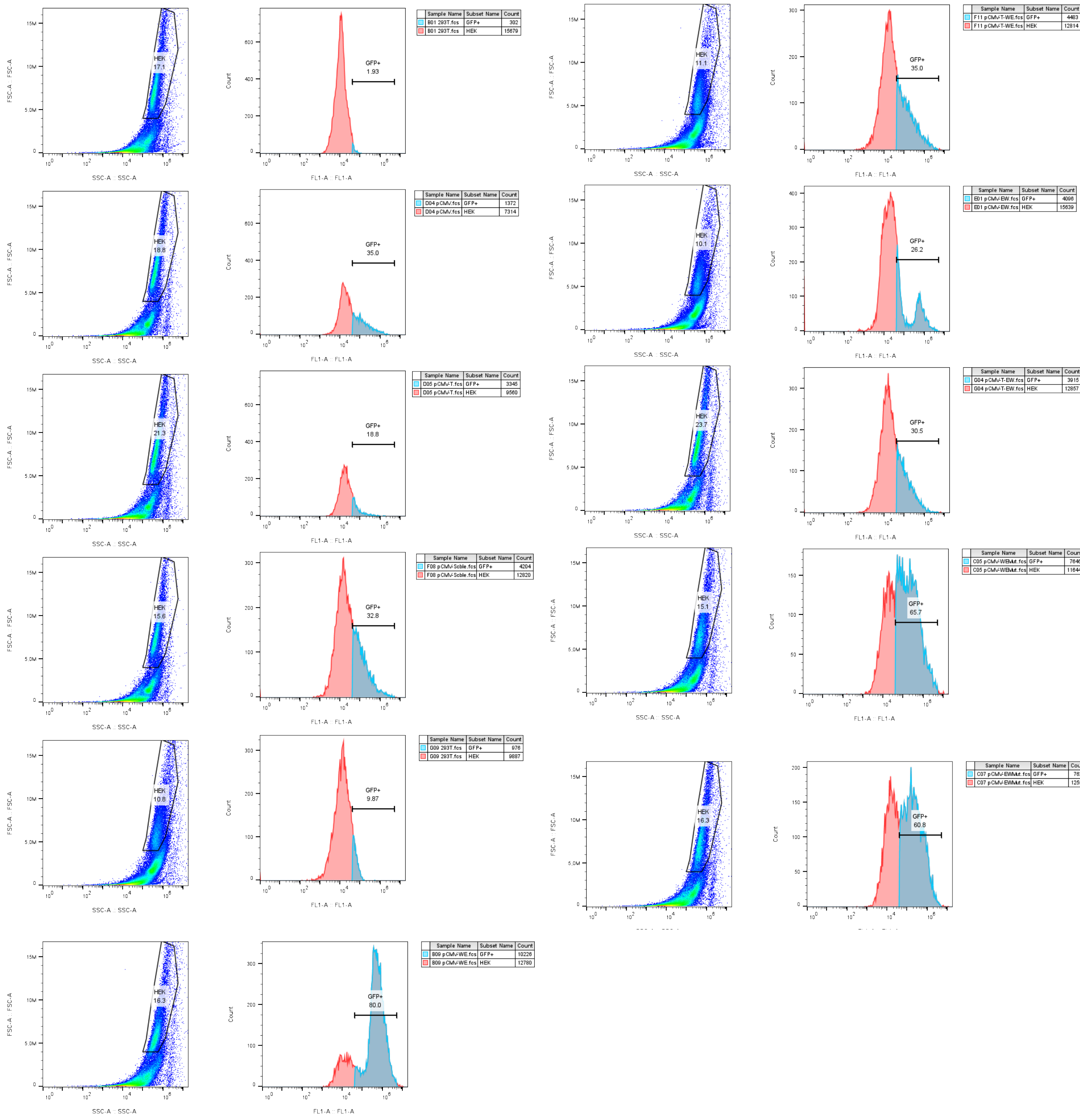

Supplemental Figure 7.

Representative flow cytometry plots of cells (HEK 293) stably transfected with respective constructs of dsDNA motifs (WE, EW, Scble, WEMut, EWMut) cloned in a reporter vector without (pCMV) or with (pCMVTelo) a telomere seed used for testing position and telomeric position effect, respectively. We report the complete flow chart of live cells sorted using FSC-A and SSC-A parameters (left) along with histograms (right) reporting the proportion of eGFP positive cells (FL1-A; in blue) within the selected population. The threshold of eGFP detection was determined using untransfected HEK 293 (eGFP-negative cells). The same gates (population, eGFP+ cells) were kept for all analyses. Associated quantifications are reported in Figure 3.

Supplemental Figure 8.

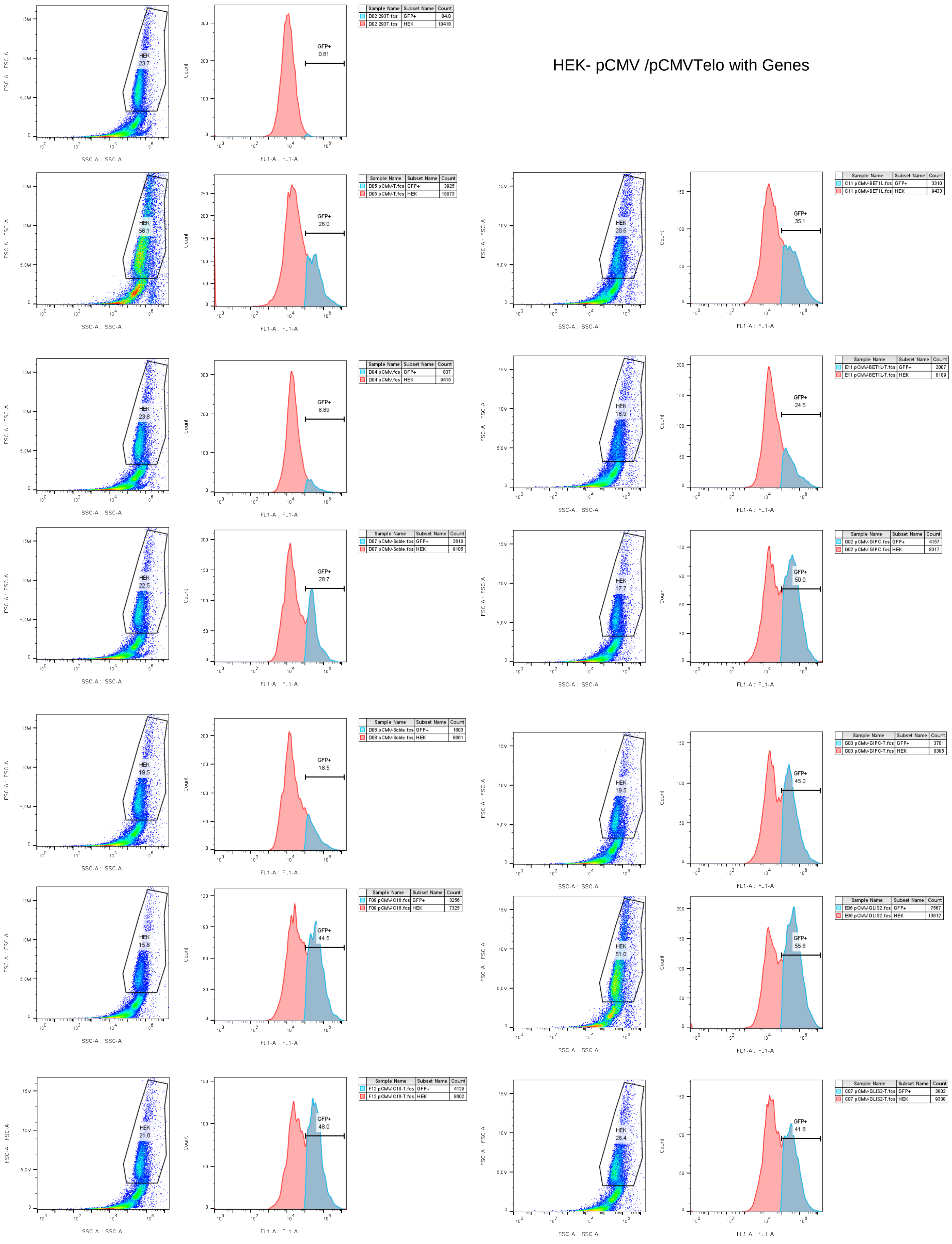

Supplemental Figure 8.

Representative flow cytometry plots of cells (HEK 293) stably transfected with respective constructs containing dsDNA motifs found in direct proximity of DEGs from our transcriptomic analysis (*C16Orf*; *BET1L*; *GIPC3*; *GLIS2*) cloned in a reporter vector without (pCMV) or with (pCMVTelo) a telomere seed for testing position and telomeric position effect, respectively. We report the complete flow chart of cells sorted using FSC-A and SSC-A parameters (left) along with histograms (right) reporting the proportion of eGFP positive cells (FL1-A; in blue) with the complete selected population (live cells). Negative eGFP cells were determined by using untransfected HEK 293 cells. The same gates (population, eGFP+) were kept for all analyses. Associated quantifications are reported in Figure 4.

Supplemental Figure 9.

HEK- pCMV – WE

siRNA Treatment

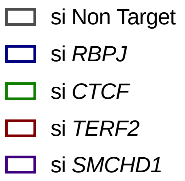

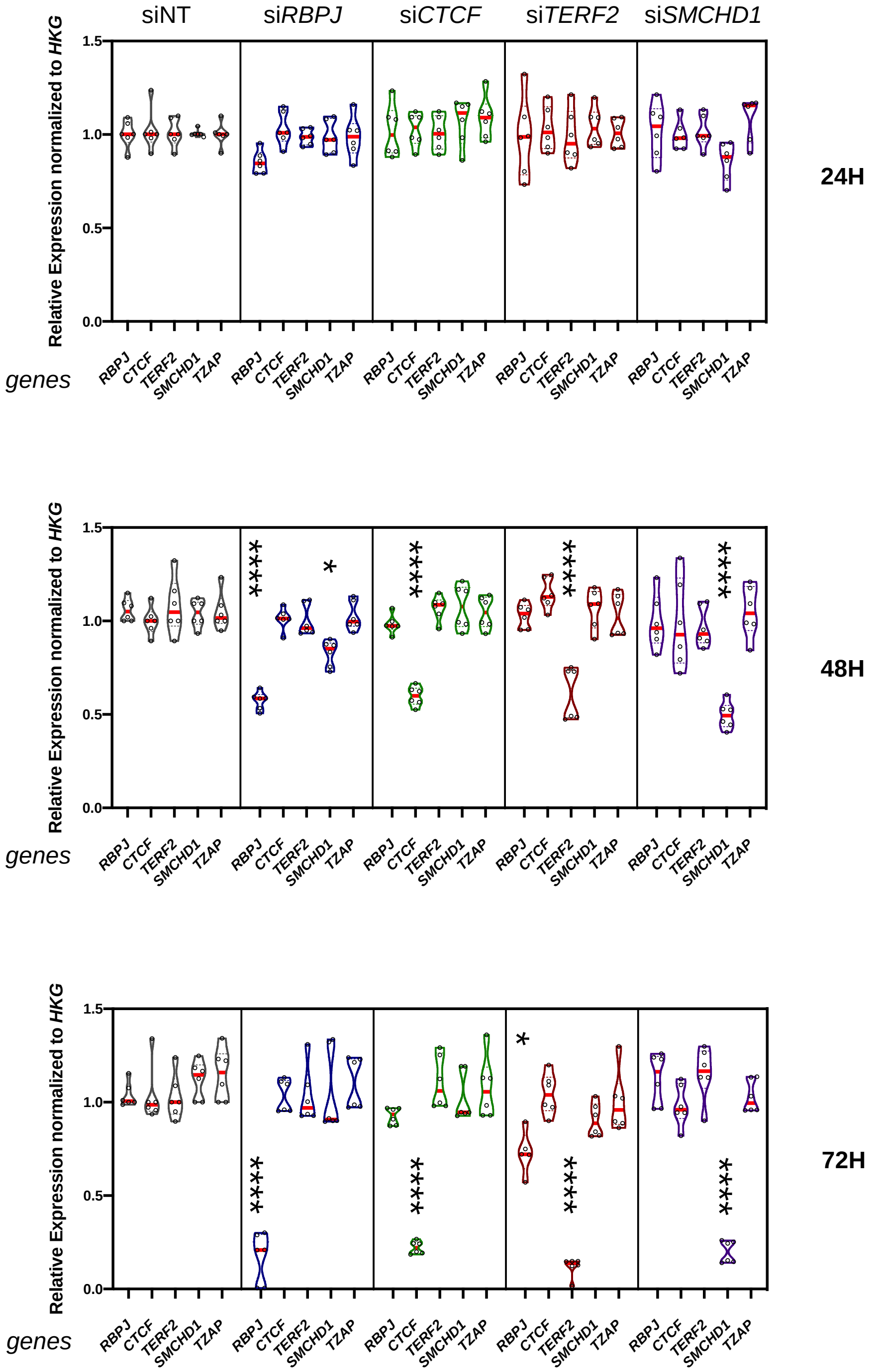

Supplemental Figure 9.

Relative gene expression (RT-qPCR) in HEK 293 cells transfected with siRNAs targeting *SMCHD1*, *RBPJ* and *TERF2*; respectively. Expression of selected genes (*RBPJ*, *TERF2*, *SMCHD1, TZAP*) is normalized to housekeeping genes (HKG; *PPIA*, *HPRT*, *GAPDH*) and respective expression at 24, 48 and 72 hours in the Non-targeted siRNA condition. For each condition, we report the average of three independent assays with technical duplicates. Mean ± SEM are shown. Holm-Sidak's multiple comparison test; α = 0.05. *p* * <0,05; *p* ** <0,005; *p* *** <0,001; *p* **** <0,0001.

Supplemental Figure 10.

HEK- pCMV - Scble
siRNA Treatment

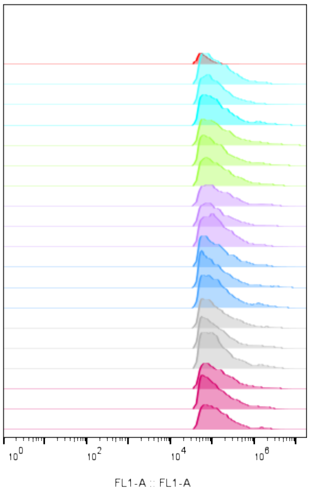

	Sample Name	Subset Name	Count
	B02 293T.fcs	GFP	375
	B05 pCMV_Sclbe NT 24.fcs	GFP	2542
	C06 pCMV_Sclbe NT 48.fcs	GFP	2279
	F02 pCMV_Sclbe NT 72.fcs	GFP	2485
	B11 pCMV_Sclbe CTCF 24.fcs	GFP	2353
	C08 pCMV_Sclbe CTCF 48.fcs	GFP	2404
	E10 pCMV_Sclbe CTCF 72.fcs	GFP	2417
	C02 pCMV_Sclbe TZAP 24.fcs	GFP	1917
	C10 pCMV_Sclbe TZAP 48.fcs	GFP	1878
	E04 pCMV_Sclbe TZAP 72.fcs	GFP	2752
	B09 pCMV_Sclbe TRF2 24.fcs	GFP	2477
	D02 pCMV_Sclbe TERF2 48.fcs	GFP	2474
	F07 pCMV_Sclbe TERF2 72.fcs	GFP	2779
	B04 pCMV_Sclbe RBPJ 24.fcs	GFP	2300
	C03 pCMV_Sclbe RBPJ 48.fcs	GFP	2375
	F11 pCMV_Sclbe RBPJ 72.fcs	GFP	3070
	B07 pCMV_Sclbe SMCHD1 24.fcs	GFP	2125
	C11 pCMV_Sclbe SMCHD1 48.fcs	GFP	2560
	E03 pCMV_Sclbe TZAP 72.fcs	GFP	2030

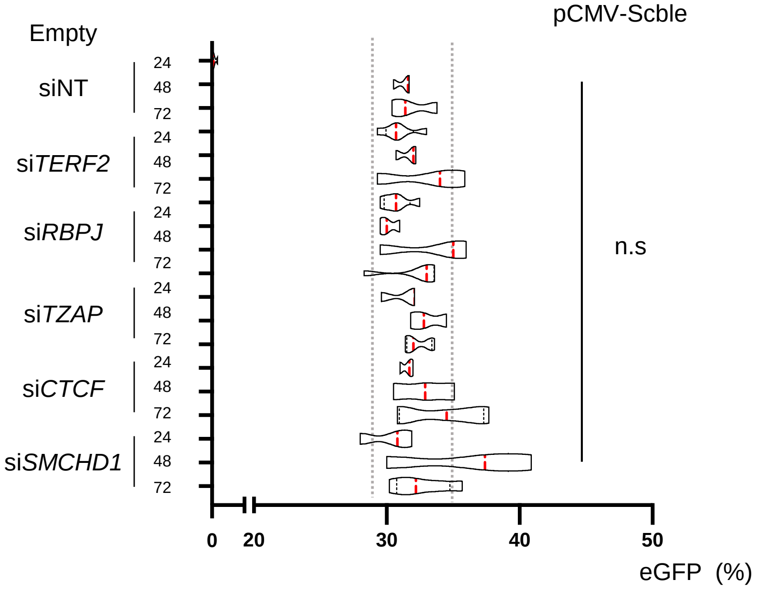

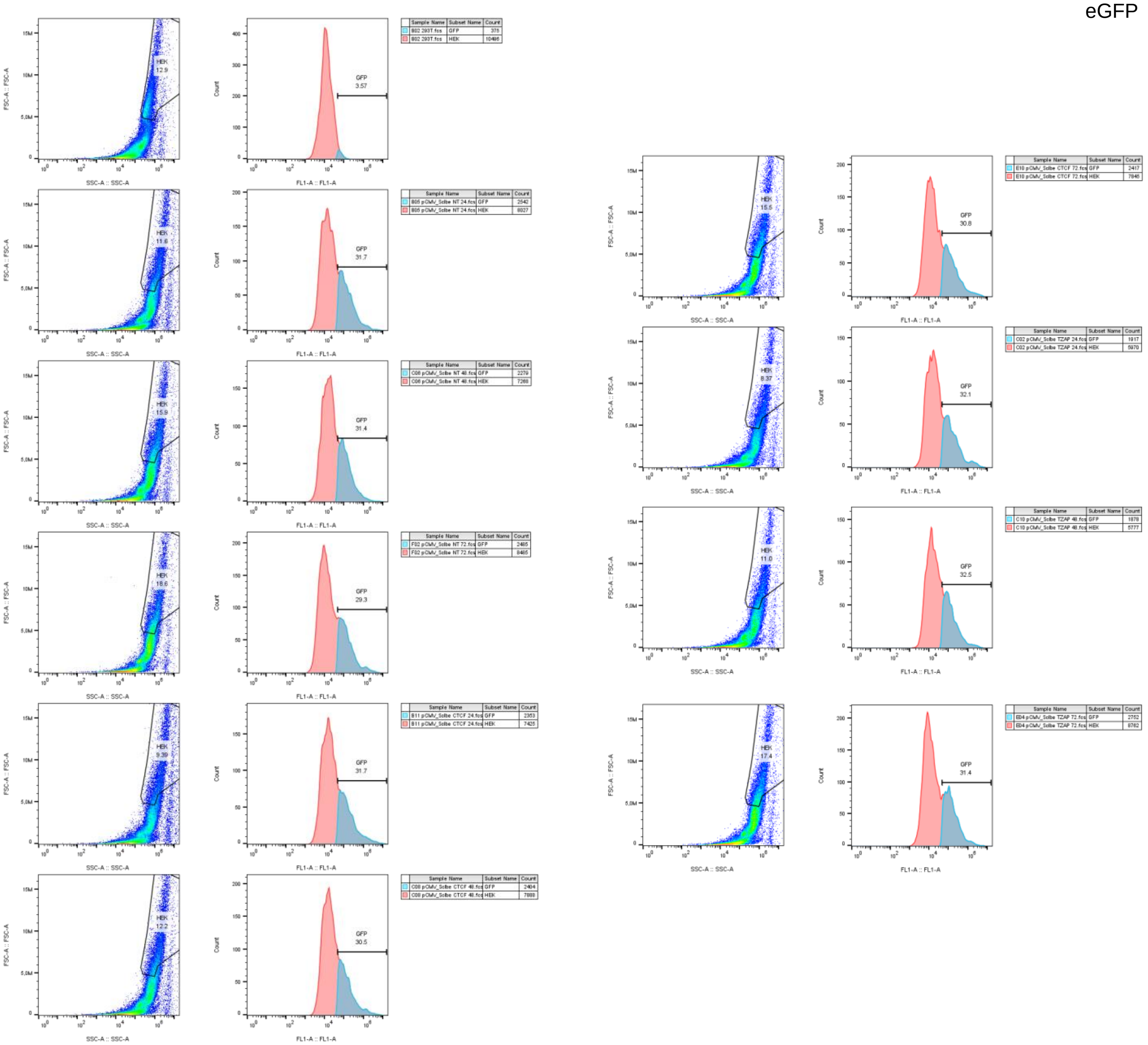

Supplemental Figure 10.

HEK- pCMV - Scble
siRNA Treatment

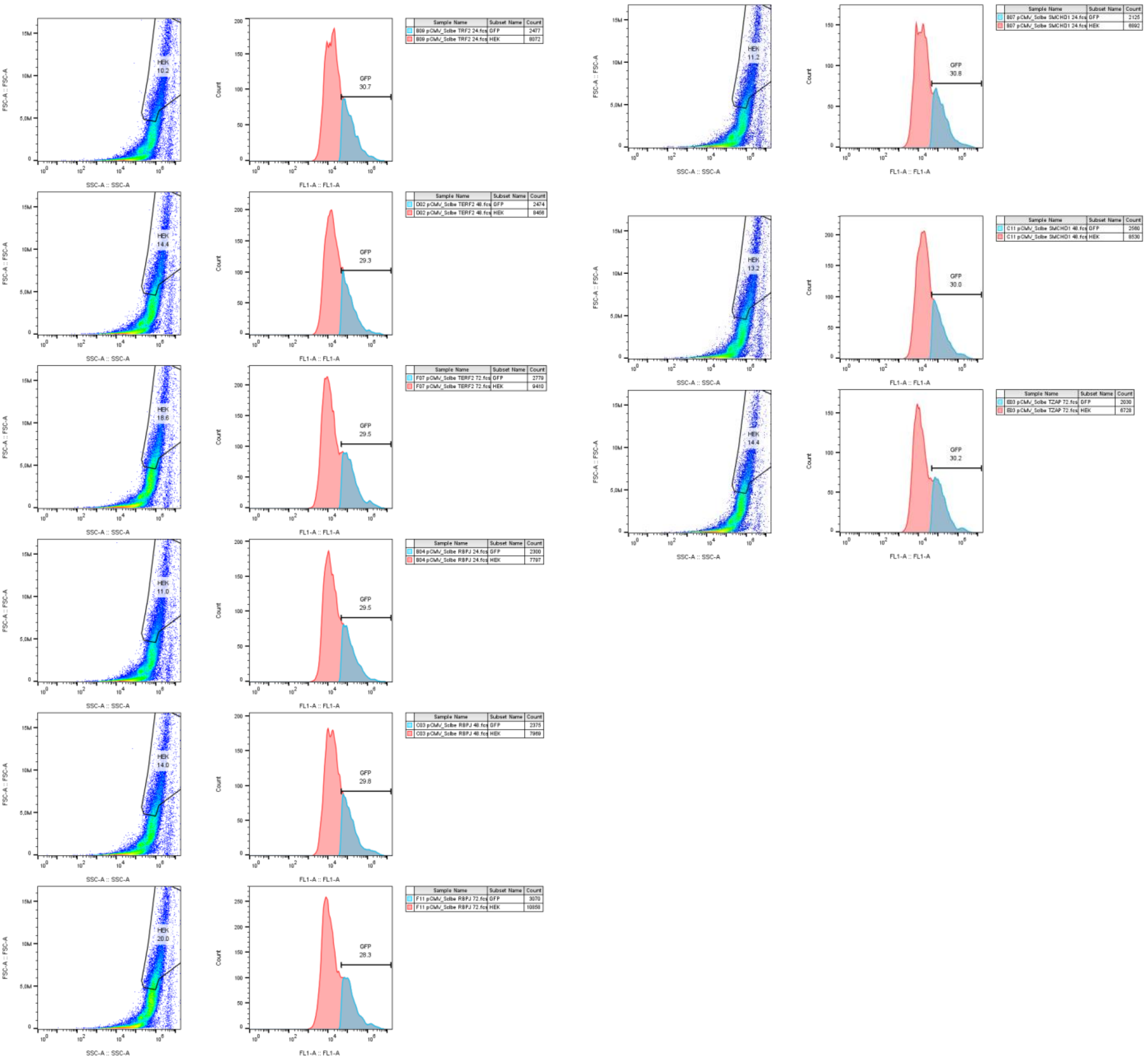

Supplemental Figure 10.
Representative flow cytometry plots of cells (HEK 293) stably transfected with a dsDNA motif (Scble condition, as described in Figure 2.) inserted in a pCMV reporter plasmid and transfected with siRNAs along with their associated quantifications. We report the complete flow charts of cells sorted using FSC-A and SSC-A parameters (left) along with histograms (right) reporting the proportion of eGFP-positive cells (FL1-A; in blue) at 24, 48 and 72 hours post treatments. Negative eGFP cells were determined by using untransfected HEK 293 cells. The same gates (population, eGFP+) were kept for all analyses. For each condition, we report biological quadruplicates (n=4) in box and violins. Medians and quartiles are shown (red and dashed lines, respectively); dashed grey lines are shown to represent the variability associated with the NT condition. Tamhane’s T2 multiple comparisons test; α = 0.05.

Supplemental Figure 11.

HEK- pCMV - WE
siRNA Treatment

	Sample Name	Subset Name	Count
	B01 HEK.fcs	GFP+	127
	B03 pCMV.fcs	GFP+	2609
	B09 pCMV-WE.fcs	GFP+	9722
	C01 pCMV-WE-NT 24H.fcs	GFP+	9291
	C03 pCMV-WE-NT 48H.fcs	GFP+	8747
	C07 pCMV-WE-NT 72H.fcs	GFP+	9435
	D01 pCMV-WE-CTCF 24H.fcs	GFP+	9595
	D04 pCMV-WE-CTCF 48H.fcs	GFP+	9353
	D07 pCMV-WE-CTCF 72H.fcs	GFP+	9047
	H02 pCMV-WE-TZAP 24H.fcs	GFP+	9284
	H03 pCMV-WE-TZAP 48H.fcs	GFP+	9375
	H07 pCMV-WE-TZAP 72H.fcs	GFP+	9888
	E01 pCMV-WE-TERF2 24H.fcs	GFP+	9677
	E03 pCMV-WE-TERF2 48H.fcs	GFP+	8641
	E08 pCMV-WE-TERF2 72H.fcs	GFP+	7822
	G01 pCMV-WE-RBPJ 24H.fcs	GFP+	9402
	G03 pCMV-WE-RBPJ 48H.fcs	GFP+	9785
	G09 pCMV-WE-RBPJ 72H.fcs	GFP+	7987
	F01 pCMV-WE-SMCHD1 24H.fcs	GFP+	9788
	F03 pCMV-WE-SMCHD1 48H.fcs	GFP+	9041
	F08 pCMV-WE-SMCHD1 72H.fcs	GFP+	7990

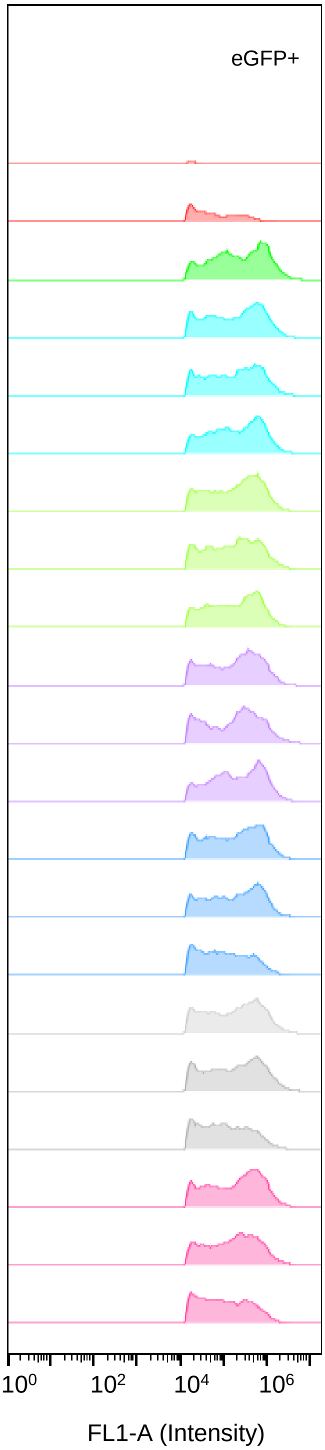

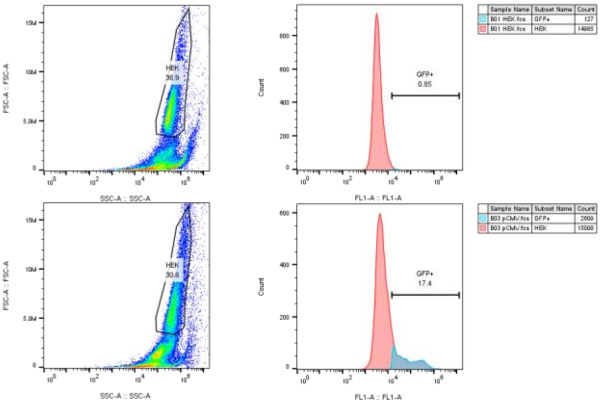

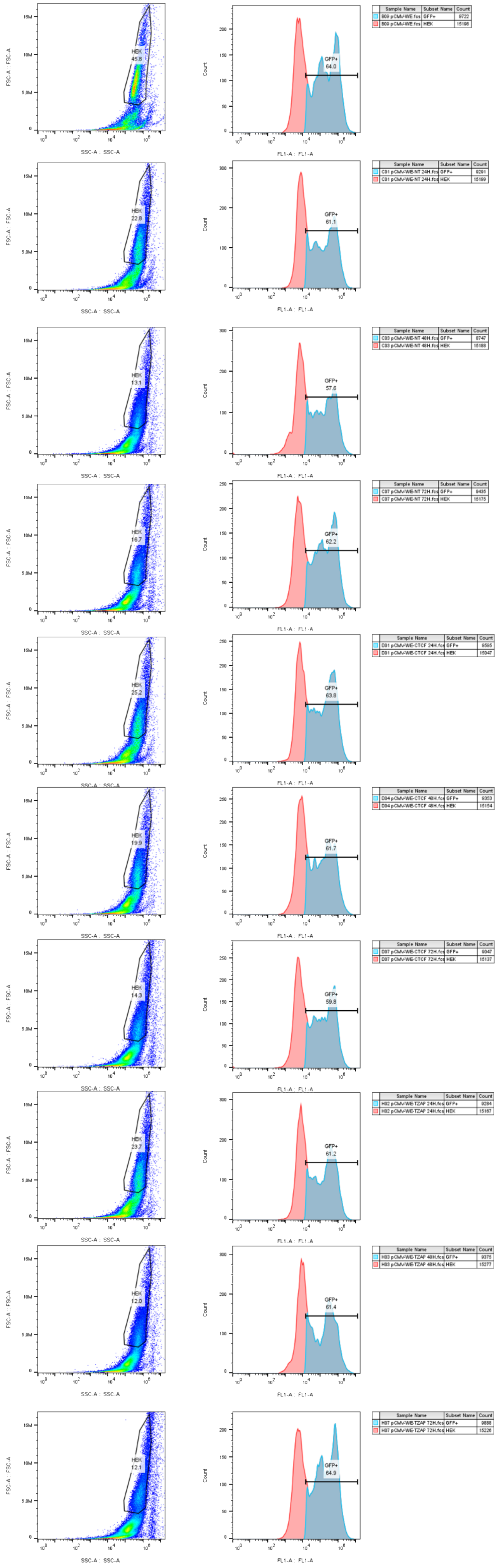

Supplemental Figure 11.

HEK- pCMV - WE
siRNA Treatment

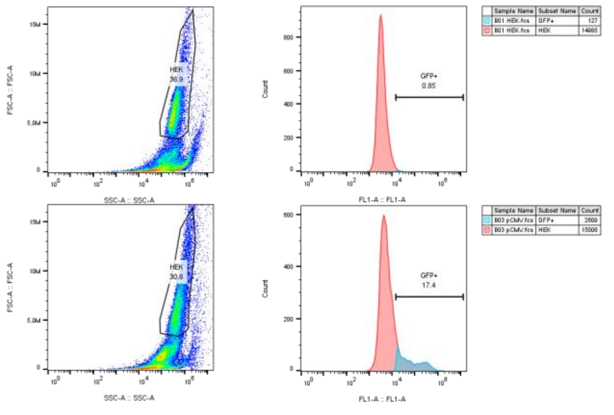

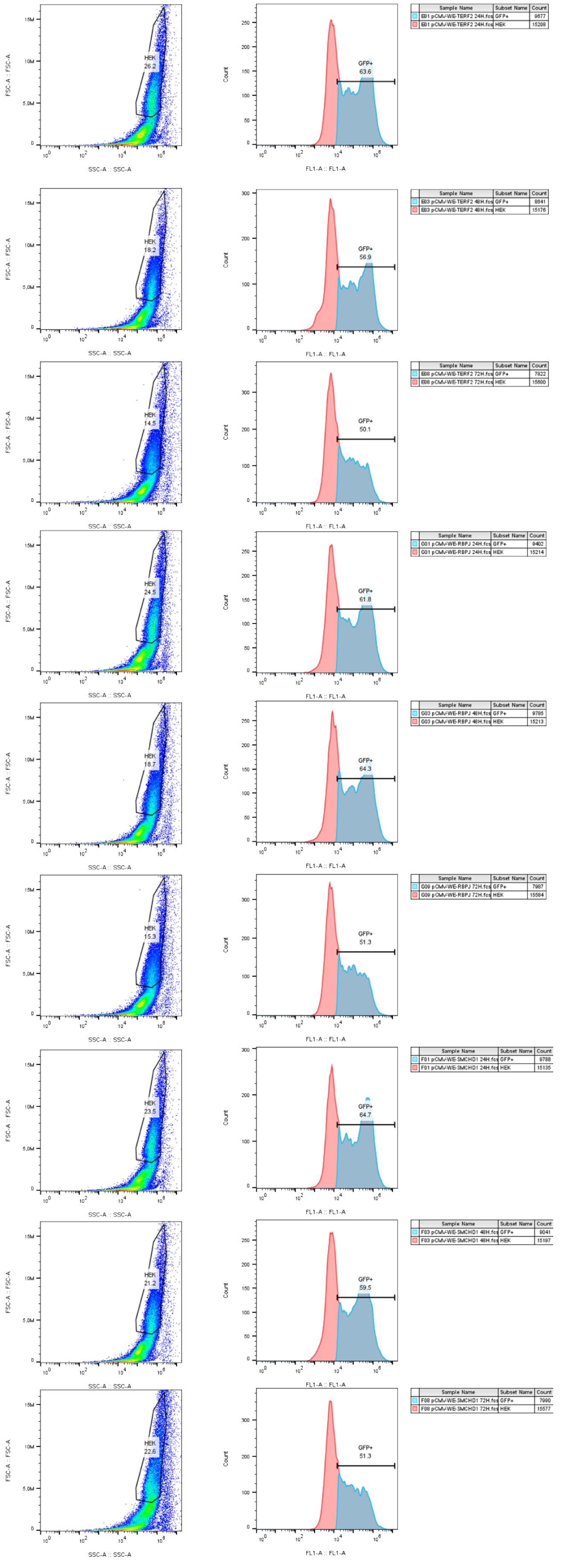

Supplemental Figure 11.
Representative flow cytometry plots of cells (HEK 293) stably transfected with a dsDNA motif (WE) inserted in a pCMV reporter plasmid and treated with siRNAs. We report the complete flow charts of cells sorted using FSC-A and SSC-A parameters (left) along with histograms (right) reporting the proportion of eGFP positive cells (FL1-A; in blue) for the selected population at 24, 48 and 72 hours post treatments. Negative eGFP cells were determined by using untransfected HEK 293 cells without transfection. The same gates (population, eGFP+) were kept for all of analyses. Associated quantifications are reported in Figure 4.

Supplemental Figure 12.

HEK- pCMV– Glis2
siRNA Treatment

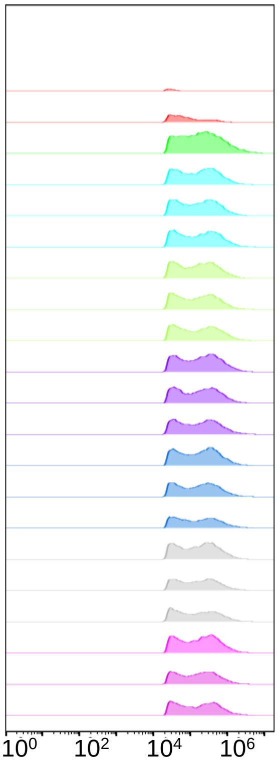

	Sample Name	Subset Name	Count
	B01 293T.fcs	GFP	248
	B03 pCMV.fcs	GFP	2427
	B04 pCMV_GLIS2.fcs	GFP	12680
	C01 pCMV_GLIS2_siNT-24H.fcs	GFP	8749
	C03 pCMV_GLIS2_siNT-48H.fcs	GFP	8815
	C07 pCMV_GLIS2_siNT-72H.fcs	GFP	8864
	D02 pCMV_GLIS2_siCTCF-24H.fcs	GFP	8672
	D03 pCMV_GLIS2_siCTCF-48H.fcs	GFP	8239
	D07 pCMV_GLIS2_siCTCF-72H.fcs	GFP	8160
	E02 pCMV_GLIS2_siTZAP-24H.fcs	GFP	9539
	E03 pCMV_GLIS2_siTZAP-48H.fcs	GFP	8314
	E09 pCMV_GLIS2_siTZAP-72H.fcs	GFP	7939
	F01 pCMV_GLIS2_siTERF2-24H.fcs	GFP	9058
	F06 pCMV_GLIS2_siTERF2-48H.fcs	GFP	7358
	F10 pCMV_GLIS2_siTERF2-72H.fcs	GFP	5807
	G01 pCMV_GLIS2_siRBPJ-24H.fcs	GFP	8614
	G05 pCMV_GLIS2_siRBPJ-48H.fcs	GFP	6246
	G09 pCMV_GLIS2_siRBPJ-72H.fcs	GFP	6221
	H01 pCMV_GLIS2_siSMCHD1-24H.fcs	GFP	9155
	H05 pCMV_GLIS2_siSMCHD1-48H.fcs	GFP	6576
	H10 pCMV_GLIS2_siSMCHD1-72H.fcs	GFP	7025

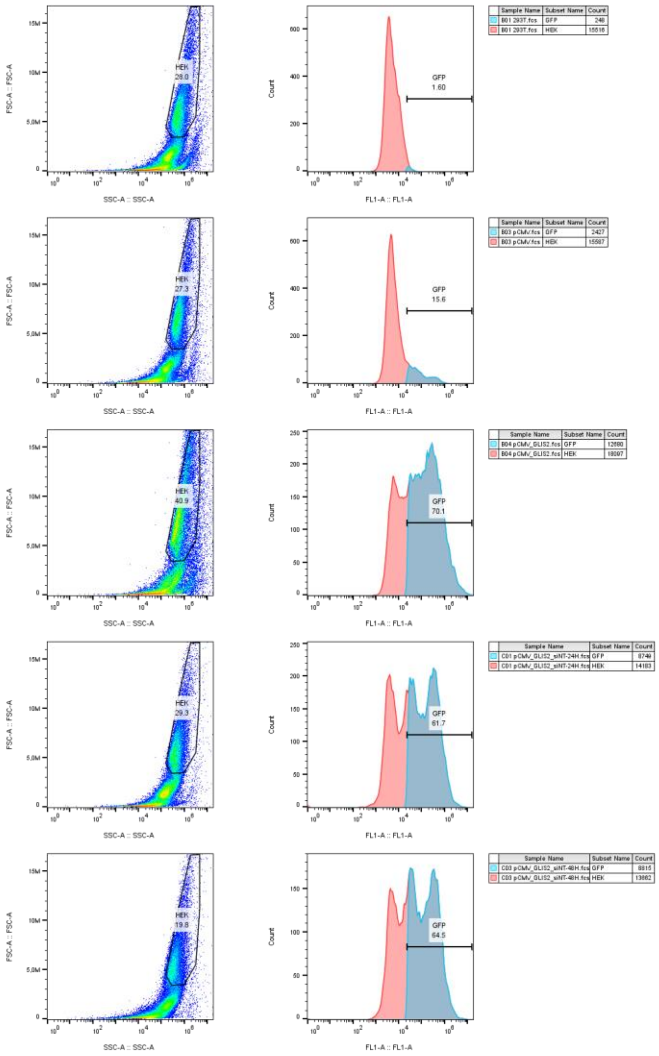

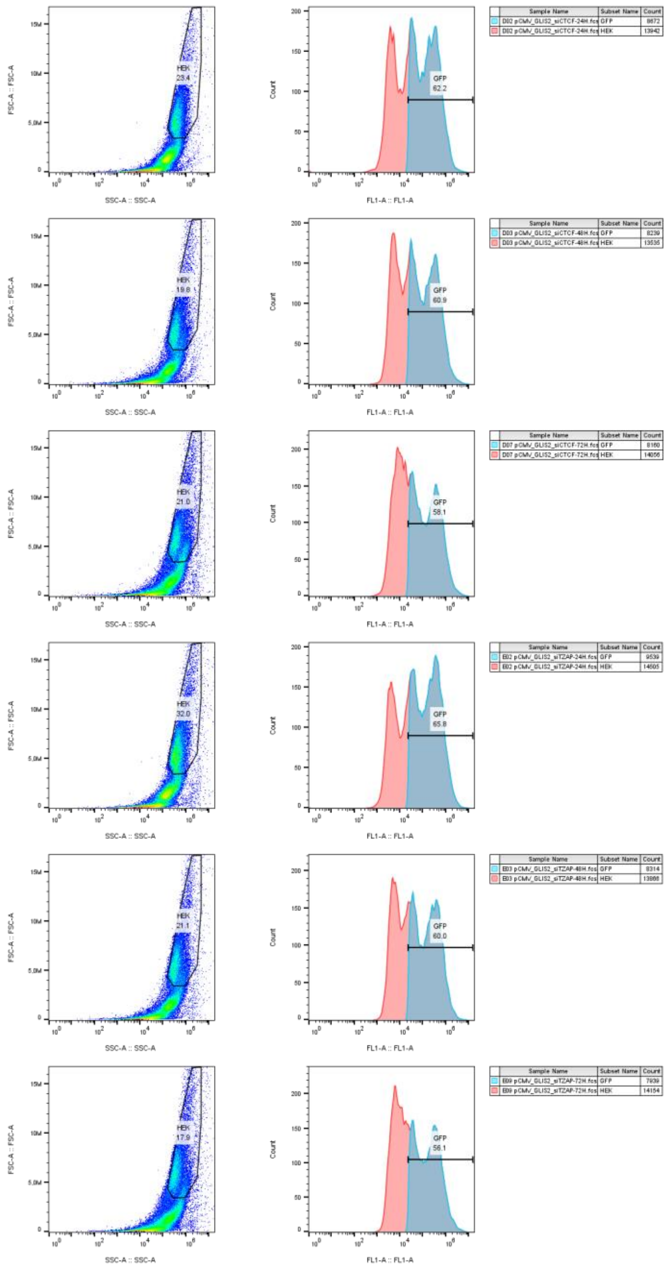

Supplemental Figure 12.

HEK- pCMV– Glis2
siRNA Treatment

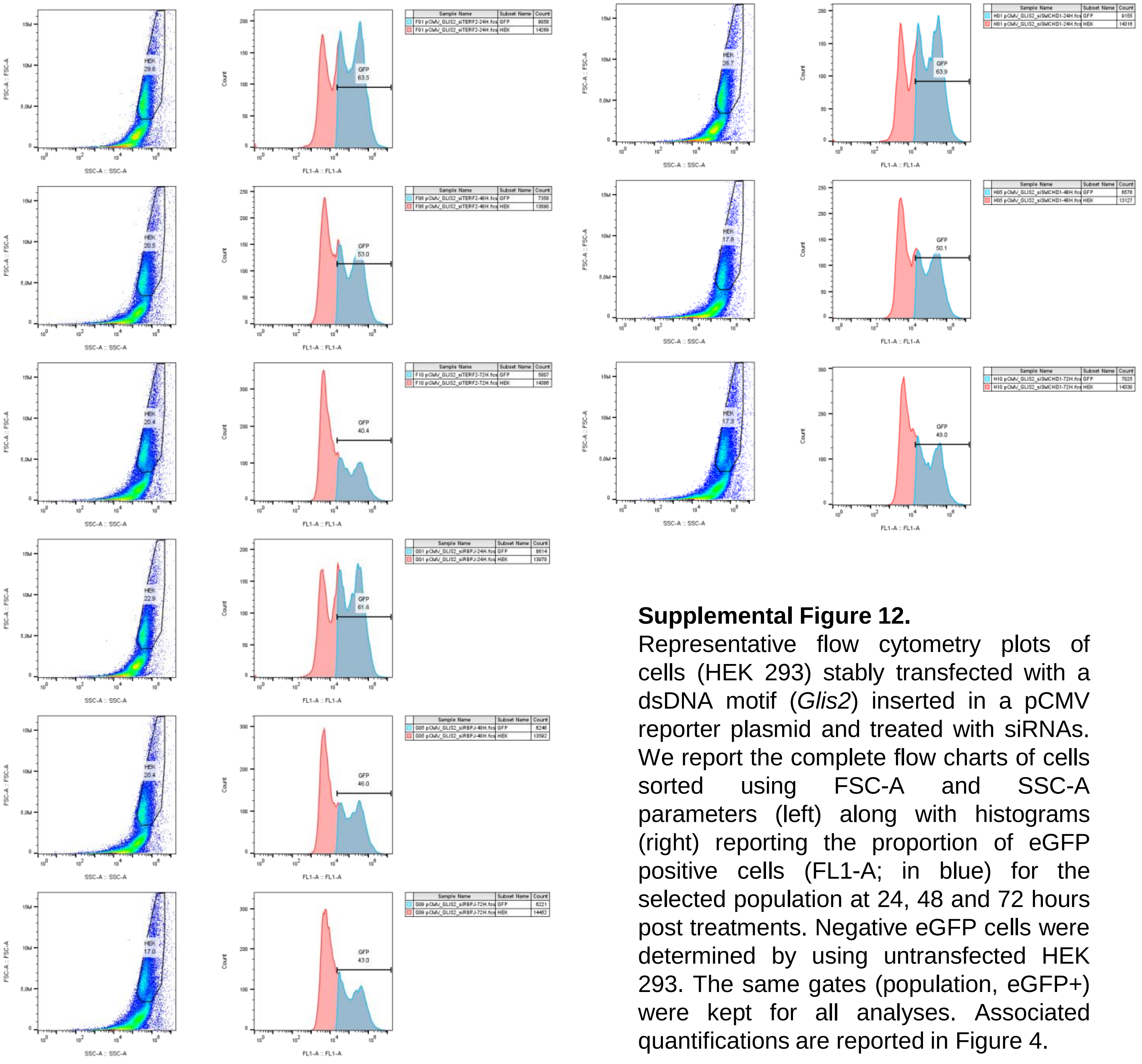

Supplemental Figure 12.
Representative flow cytometry plots of cells (HEK 293) stably transfected with a dsDNA motif (*Glis2*) inserted in a pCMV reporter plasmid and treated with siRNAs. We report the complete flow charts of cells sorted using FSC-A and SSC-A parameters (left) along with histograms (right) reporting the proportion of eGFP positive cells (FL1-A; in blue) for the selected population at 24, 48 and 72 hours post treatments. Negative eGFP cells were determined by using untransfected HEK 293. The same gates (population, eGFP+) were kept for all analyses. Associated quantifications are reported in Figure 4.

Supplemental Figure 13.

HEK- pCMVTelo – Glis2
siRNA Treatment

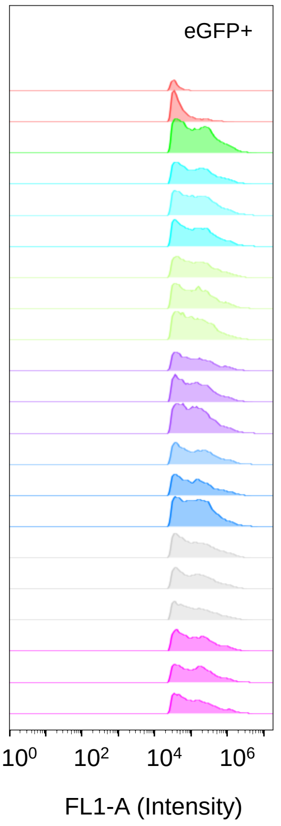

	Sample Name	Subset Name	Count
	A01 293T.fcs	GFP	695
	A03 pCMVT.fcs	GFP	3267
	A04 pCMVT_GLIS2.fcs	GFP	10439
	B01 siNT_24H.fcs	GFP	6314
	D04 siNT_72H.fcs	GFP	7744
	C02 siNT_48H.fcs	GFP	7459
	B06 siCTCF_24H.fcs	GFP	6277
	C03 siCTCF_48H.fcs	GFP	7520
	D08 siCTCF_72H.fcs	GFP	8439
	B03 siTZAP_24H.fcs	GFP	5080
	C06 siTZAP_48H.fcs	GFP	7357
	D09 siTZAP_72H.fcs	GFP	9187
	B02 siTERF2_24H.fcs	GFP	5819
	C07 siTERF2_48H.fcs	GFP	5809
	E02 siTERF2_72H.fcs	GFP	9864
	B04 siRBPJ_24H.fcs	GFP	6532
	C09 siRBPJ_48H.fcs	GFP	5734
	E09 siRBPJ_72H.fcs	GFP	4982
	B05 siSMCHD1_24H.fcs	GFP	5858
	C11 siSMCHD1_48H.fcs	GFP	5400
	E07 siSMCHD1_72H.fcs	GFP	5406

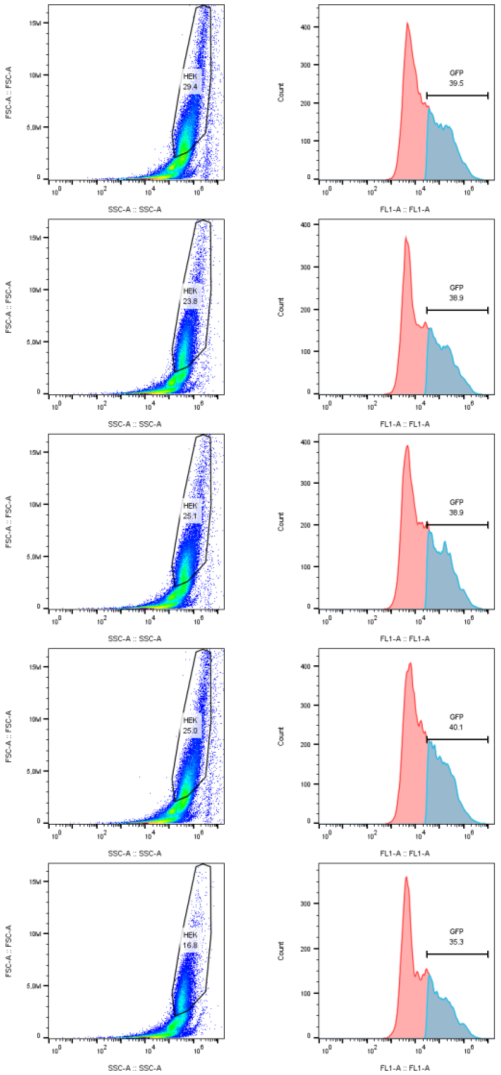

	Sample Name	Subset Name	Count
	D04 siNT_72H.fcs	GFP	7744
	D04 siNT_72H.fcs	HEK	19588

	Sample Name	Subset Name	Count
	B06 siCTCF_24H.fcs	GFP	6277
	B06 siCTCF_24H.fcs	HEK	16146

	Sample Name	Subset Name	Count
	C03 siCTCF_48H.fcs	GFP	7520
	C03 siCTCF_48H.fcs	HEK	19321

	Sample Name	Subset Name	Count
	D08 siCTCF_72H.fcs	GFP	8439
	D08 siCTCF_72H.fcs	HEK	21060

	Sample Name	Subset Name	Count
	B03 siTZAP_24H.fcs	GFP	5080
	B03 siTZAP_24H.fcs	HEK	14410

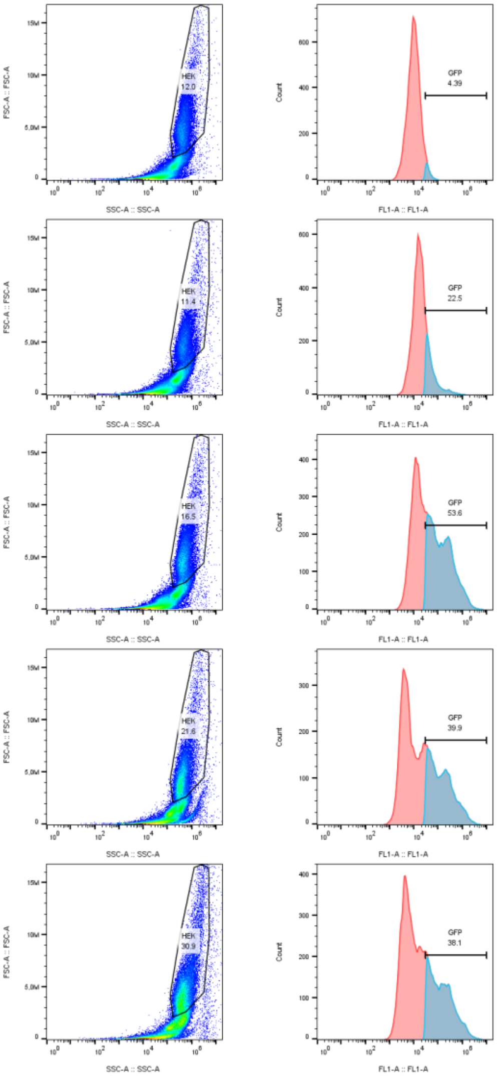

	Sample Name	Subset Name	Count
	A01 293T.fcs	GFP	695
	A01 293T.fcs	HEK	15823

	Sample Name	Subset Name	Count
	A03 pCMVT.fcs	GFP	3267
	A03 pCMVT.fcs	HEK	14514

	Sample Name	Subset Name	Count
	A04 pCMVT_GLIS2.fcs	GFP	10439
	A04 pCMVT_GLIS2.fcs	HEK	19479

	Sample Name	Subset Name	Count
	B01 siNT_24H.fcs	GFP	6314
	B01 siNT_24H.fcs	HEK	15831

	Sample Name	Subset Name	Count
	C02 siNT_48H.fcs	GFP	7459
	C02 siNT_48H.fcs	HEK	19575

Supplemental Figure 13.

HEK- pCMVTelo – Glis2

siRNA Treatment

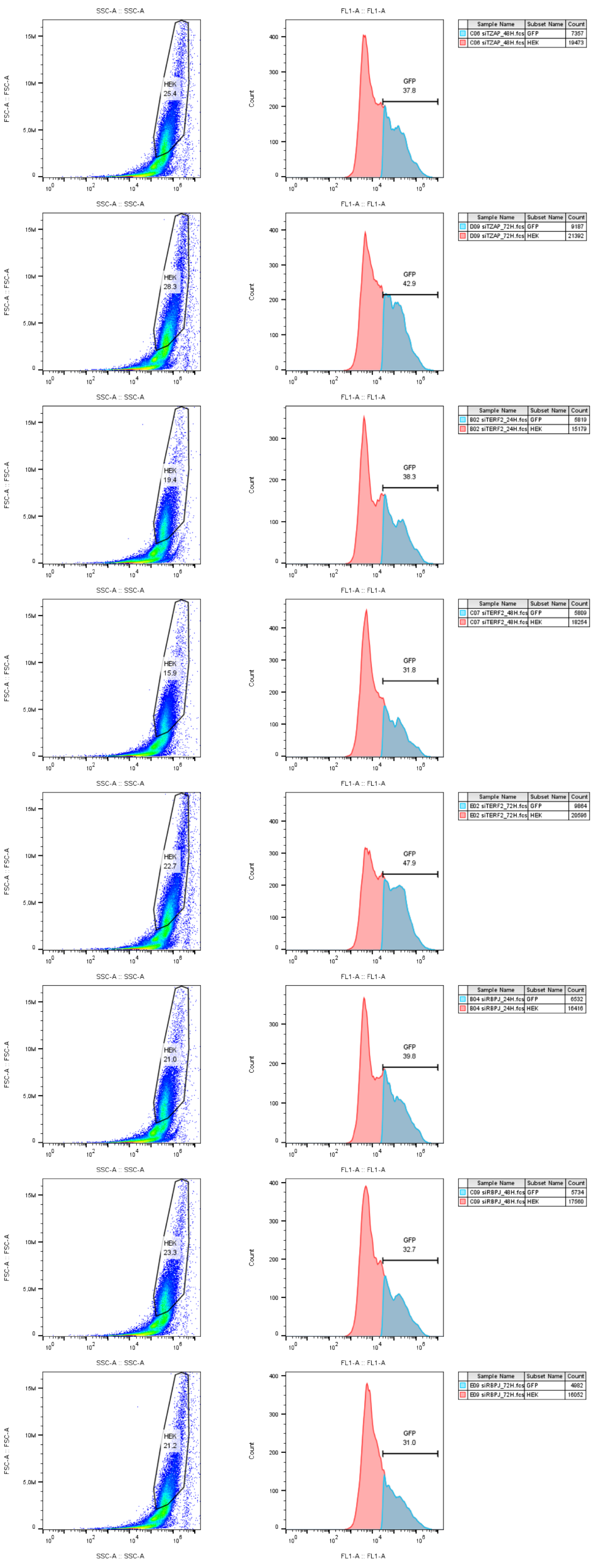

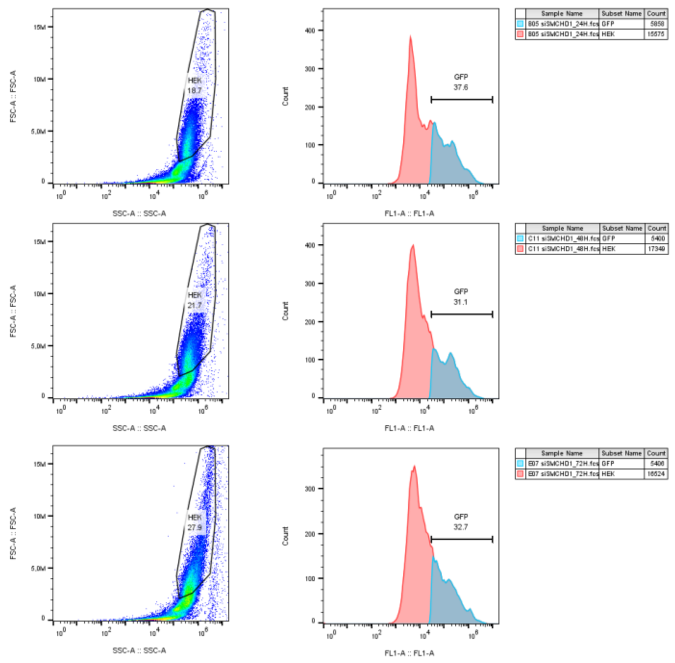

Supplemental Figure 13.
Representative flow cytometry plots of cells (HEK 293) stably transfected with a dsDNA motif (Glis2) inserted in a pCMVTelo (with a telomere seed) reporter plasmid and treated with siRNAs. We report the complete flow charts of cells sorted using FSC-A and SSC-A parameters (left) along with histograms (right) reporting the proportion of eGFP positive cells (FL1-A; in blue) for the selected population at 24, 48 and 72 hours post treatments. Negative eGFP cells were determined by using untransfected HEK 293. The same gates (population, eGFP+) were kept all analyses. Associated quantifications are reported in Figure 4.

Supplemental Figure 14.

A

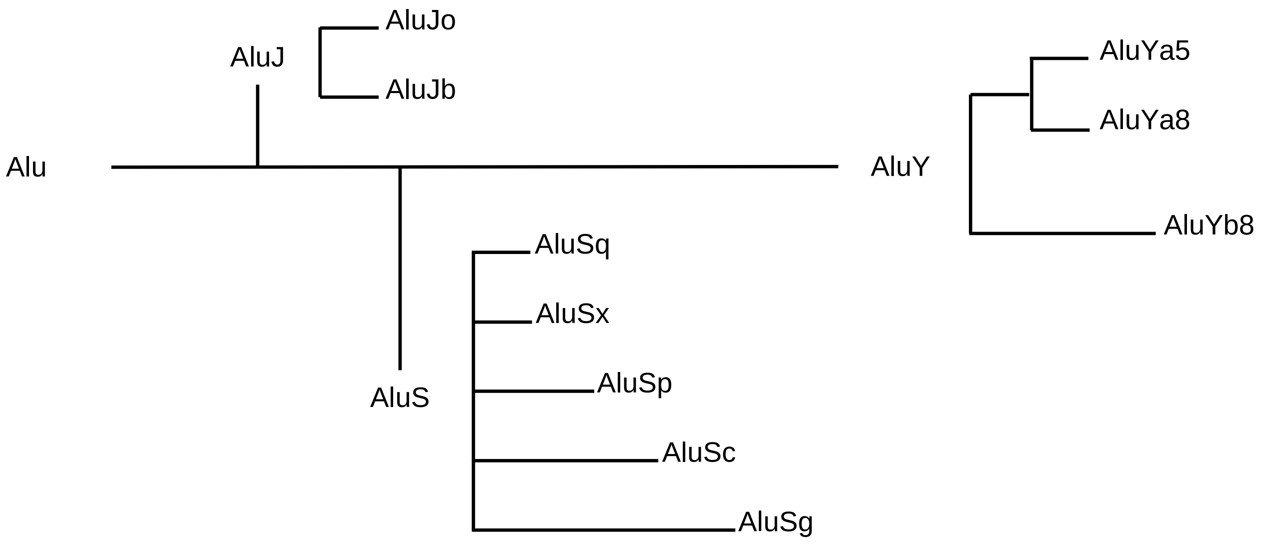

Alu Subclass	Copies/ genome
AluJ	160.000
AluS	650.000
AluY	155.000

B

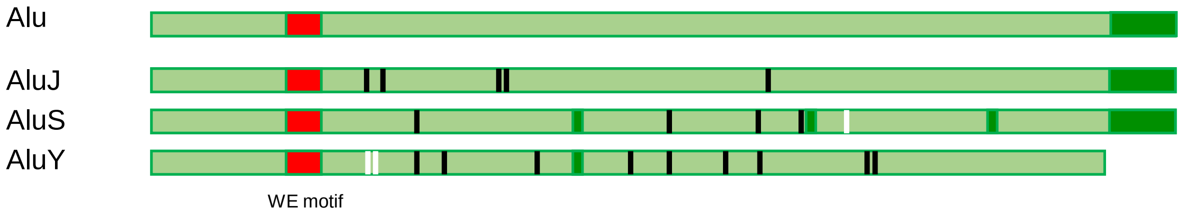

Supplemental Figure 14.
A. Approximate evolutionary tree of *Alu* elements an subclasses as reported in Mighell AJ et al., 1997 and Deininger P et al., 2011. We report the approximate number of copies per human genome of each subclasses. **B.** Alu and subclasses consensus sequences as reported in Weisenberger DJ et al., 2005. We summarized the differences along the 300bp sequence from a theoretical Alu reference to their subclasses (highly similar to oldest *Alu; AluJ).* Nucleotides changes are reported by a black line; deletions by withe lines; insertion by dark green blocks and the WE motif by a red box. The WE element is present in all Alu. However our data report a significant enrichment of DEGs and DMPs at WE elements associated to AluY, suggesting variations induced by the surrounding sequences, beyond the WE elements. For the sake of comprehension we present only the major subclass (*i.e.*, J, S and Y).

Supplemental Figure 15.

A

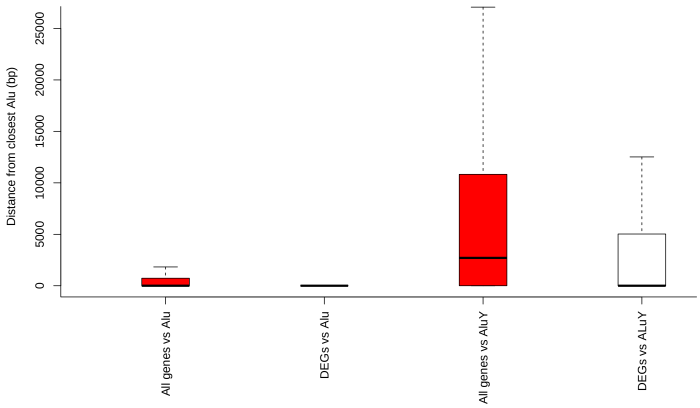

Total Genes	n= 60600
DEGs	n= 1811
Alu	n= 1 262 425
AluY	n= 149 129

B

ALU

**hTERT**	68.5
**12kb**	62.8
**10kb**	60.5
**8kb**	60.5
**6kb**	59.9
**4kb**	59

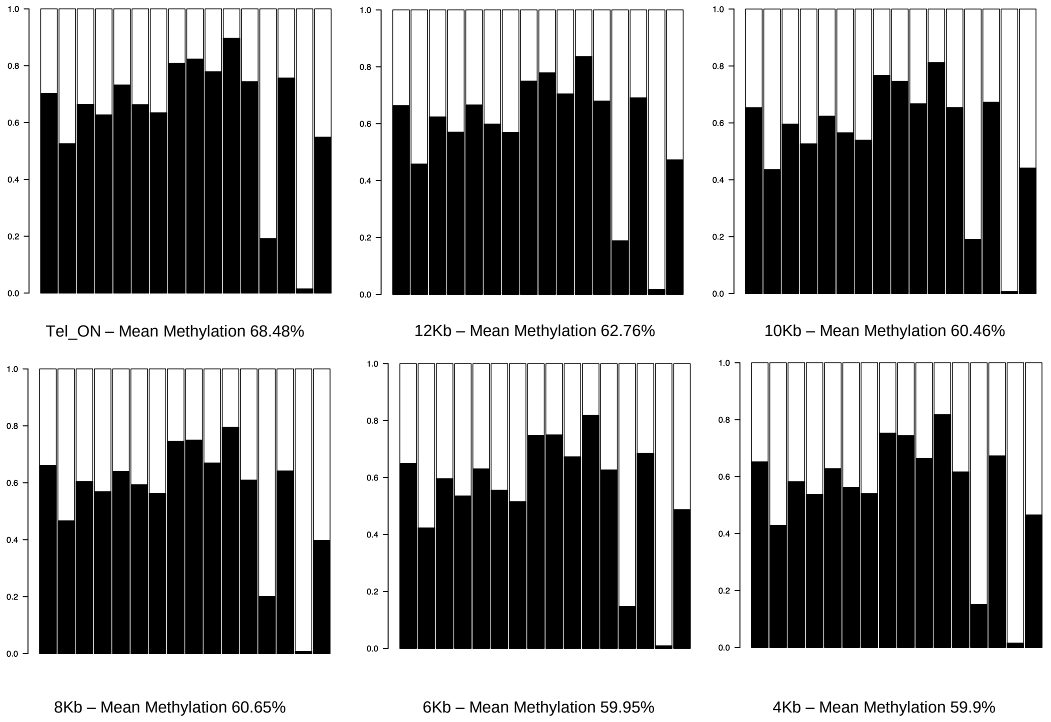

TAR1

**hTERT**	76.9
**12kb**	76.9
**10kb**	76.8
**8kb**	76.1
**6kb**	77.7
**4kb**	76.7

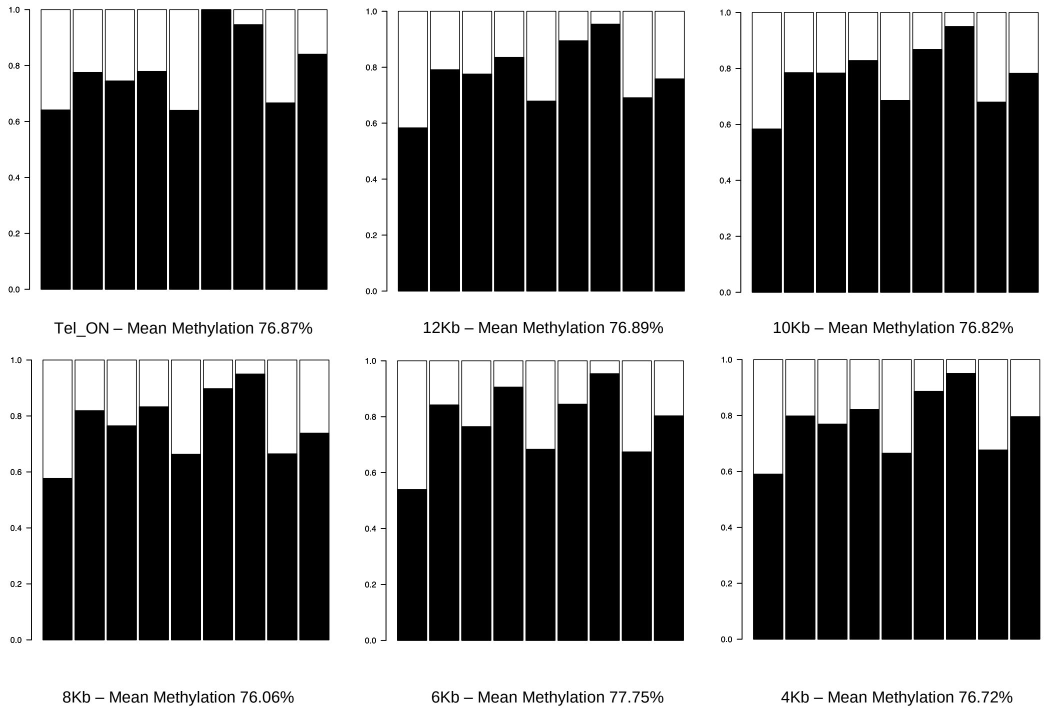

Supplemental Figure 15.

A. Distance of genes and DEGs to the closest Alu or AluY elements. Distances were calculated using the total number of genes (n=60,600) or DEGs from our experiments (n=1,811) and reference location of either Alu (n= 1,262,425) or AluY (n= 149,129). For Alu comparison; Welch Two Sample t-test, *p-value* < 2.2e-16; for AluY, Welch Two Sample t-test, p-value < 2.2e-16. **B.** Stacked barplots representing the distribution of methylation at CpGs in either Alu repeats (top panel) or TAR1 repeats (bottom panel) in isogenic clones of myoblasts with long and shorter telomeres. Methylation was assessed using sodium bisulfited-converted DNA and Bisulfite sequencing primers. A minimum of 50,000 sequences were aligned per condition.

Supplemental Figure 16.

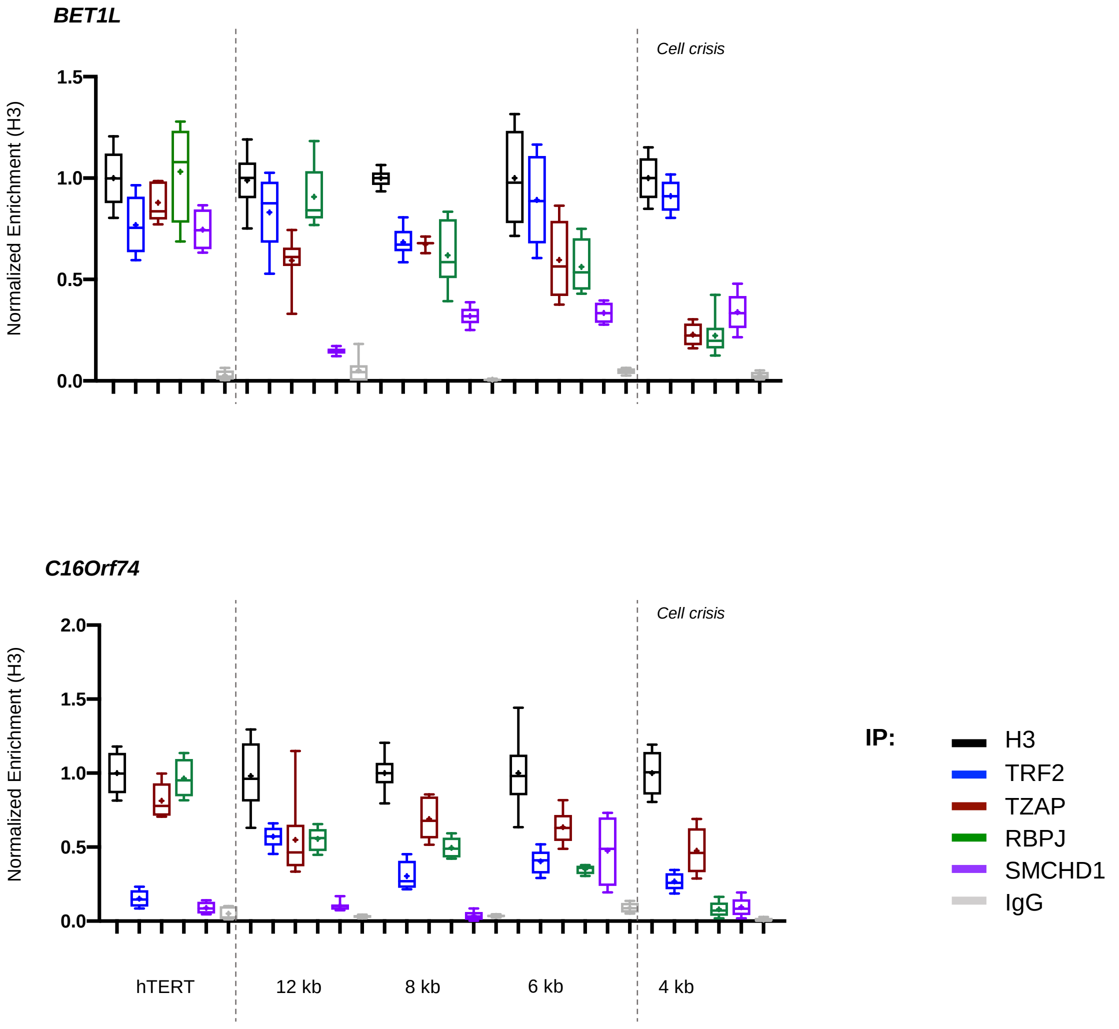

Supplemental Figure 16.
Protein enrichment at *BET1L* and *C16Orf74* loci detected by ddPCR after ChIP in isogenic clones with various telomere lengths. We report the enrichment of H3 (black), TRF2 (blue), TZAP (maroon), RBPJ (green), SMCHD1(purple) and IgG (grey) normalized to H3. Dashed grey lines are shown to represent the critical telomere length, before cell crisis (active telomerase, 4kb; respectively). ChIP-ddPCR were performed in biological quadruplicates. Holm-Sidak's multiple comparison test; α = 0.05. *p* * <0,05; *p* ** <0,005; *p* *** <0,001; *p* **** <0,0001.

Supplemental Figure 17.

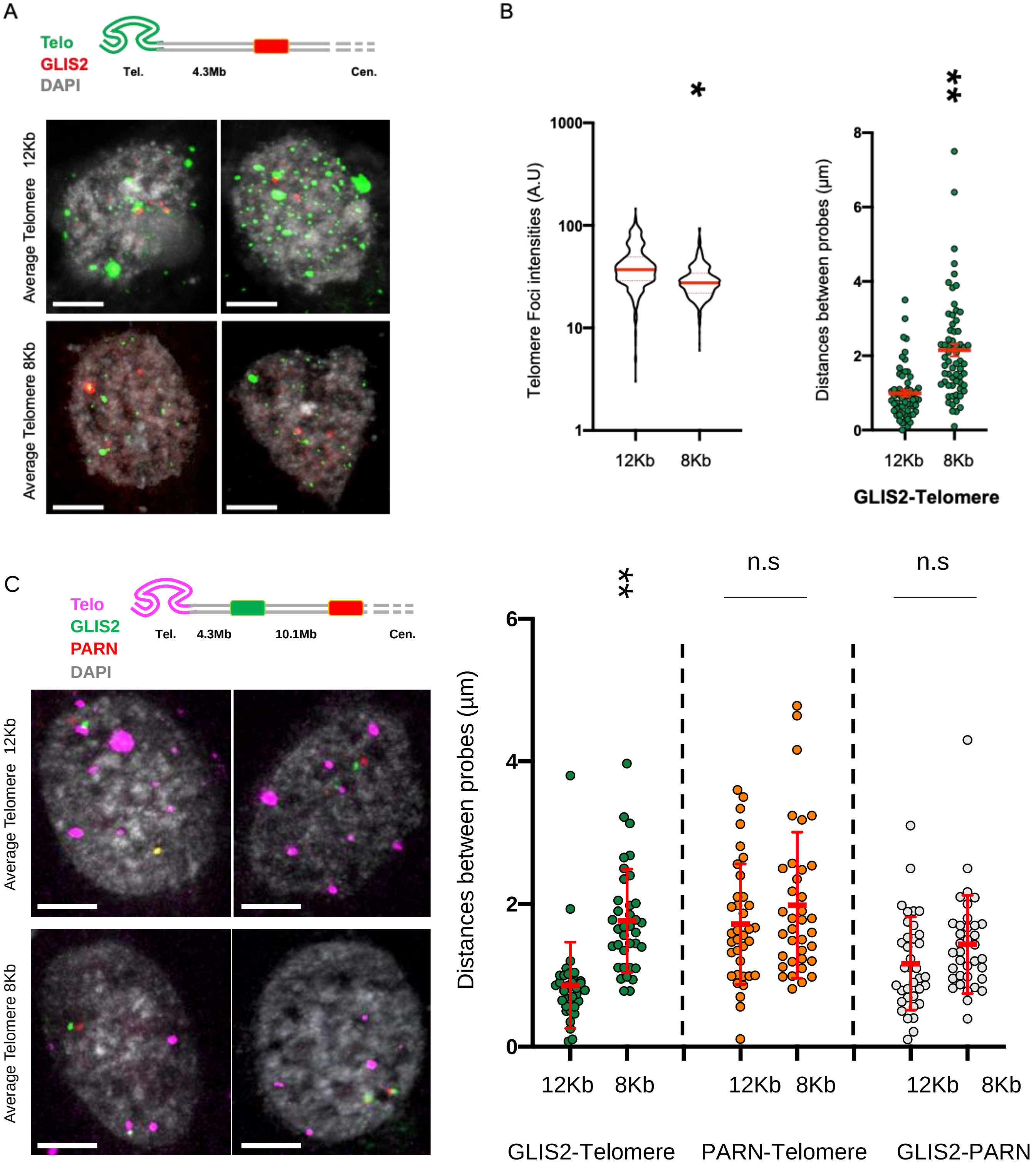

Supplemental Figure 17.

A. Graphical illustration and associated confocal images of the 16p loci color-coded with the respective probes used in myoblasts with either long (12kb) or shorter (8kb) telomeres. Briefly, 40 pictures of nuclei were taken using a telomeric (green) and GLIS2 (red) PNA probes. Each Z-stack picture was then reconstructed using the IMARIS software and analyzed. B. Quantification of telomeric intensities along with distances between the closest telomeric signal and probe are reported. The distance between GLIS2 and closest telomere is increased in cells with shorter telomeres. C. Graphical illustration (top) and associated confocal images of the 16p loci color coded with the respective probes used in myoblasts with either long (12 Kb) or shorter (8 Kb) telomeres (bottom). Briefly, 40 pictures of nuclei were taken using telomeric (purple); *GLIS2* (green) and *PARN* (red) probes. Quantifications are reported by measuring the distances between the closest telomeric signal to probe for either *GLIS2* or *PARN* along with the distance between *PARN* and *GLIS2*. Student T-test; $\alpha = 0.05$. *p* ** <0,005.

Supplemental Figure 18. siRNA *Myoblasts*

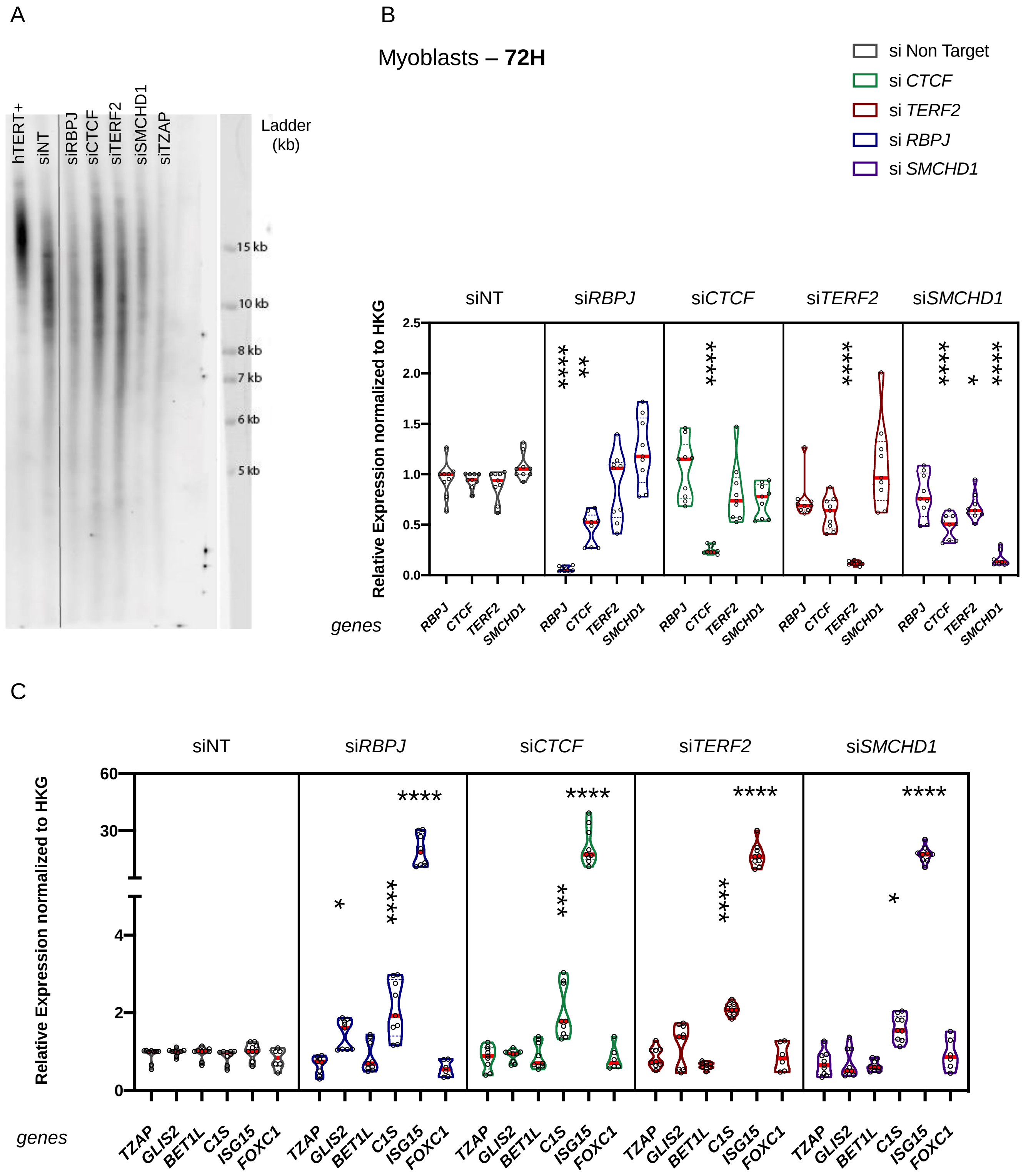

Supplemental Figure 18.

A. Representative Telomere Restriction Fragment analysis (TRF) of isogenic clones used for the siRNA experiment presented (Figure. 7). For each assay, myoblasts with long telomeres were used (12 Kb). For TRF analysis respective siRNA condition were pooled to reach minimal DNA amount **B.** Associated **r**elative gene expression (RT-qPCR) in myoblasts transfected with siRNAs targeting *SMCHD1*, *RBPJ, CTCF* and *TERF2*; respectively at 72H post transfection. Expression of selected genes is normalized to housekeeping genes (HKG; *PPIA*, *HPRT*, *GAPDH*) and respective expression in the non-targeted siRNA condition. **C.** Associated relative gene expression of selected genes. For each condition, we report the average of three independent siRNA assays with technical duplicates. Mean ± SEM are shown. Holm-Sidak's multiple comparison test; α = 0.05. *p* * <0,05; *p* ** <0,005; *p* *** <0,001; *p* **** <0,0001.

Supplemental Figure 19.

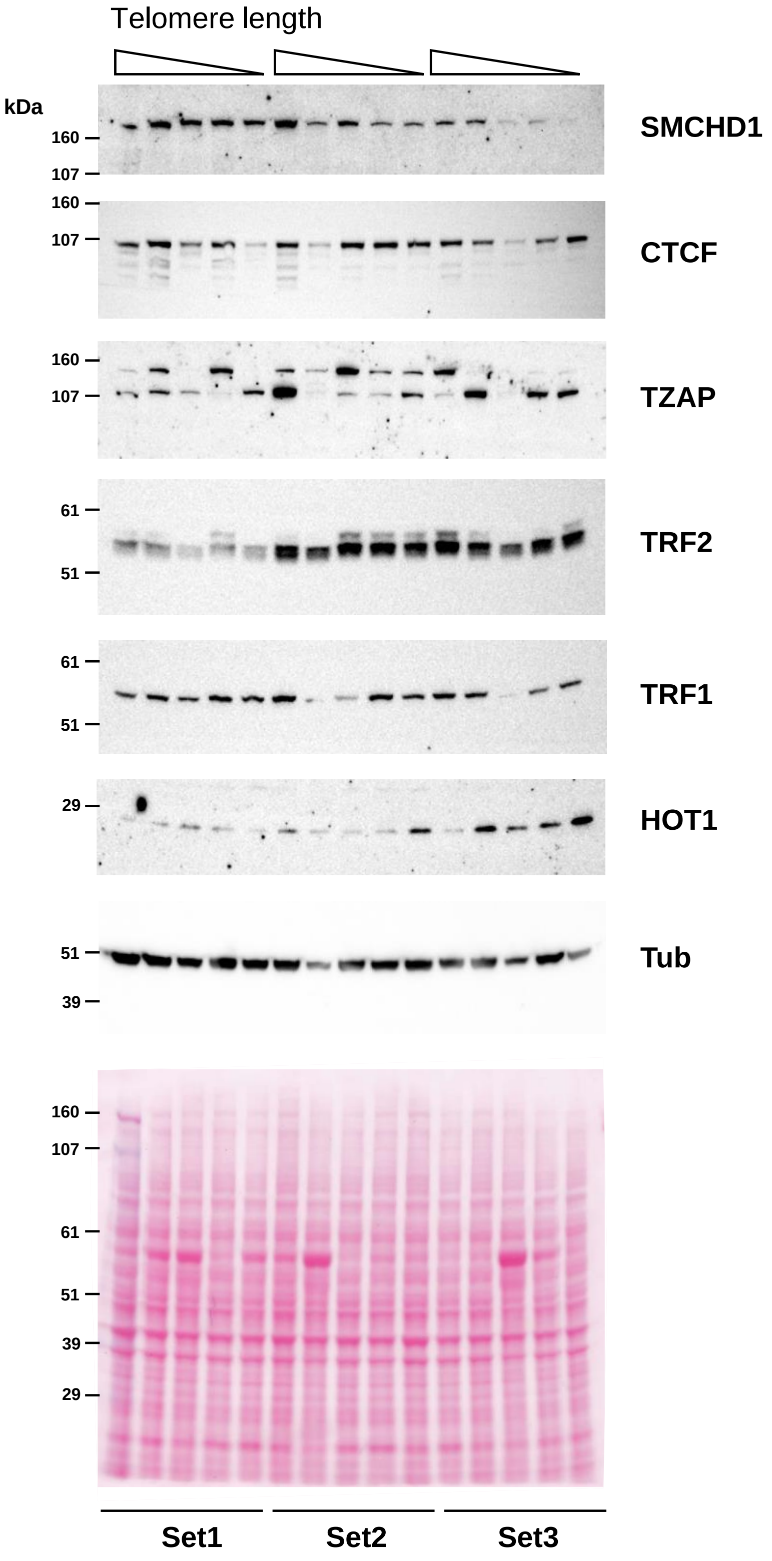

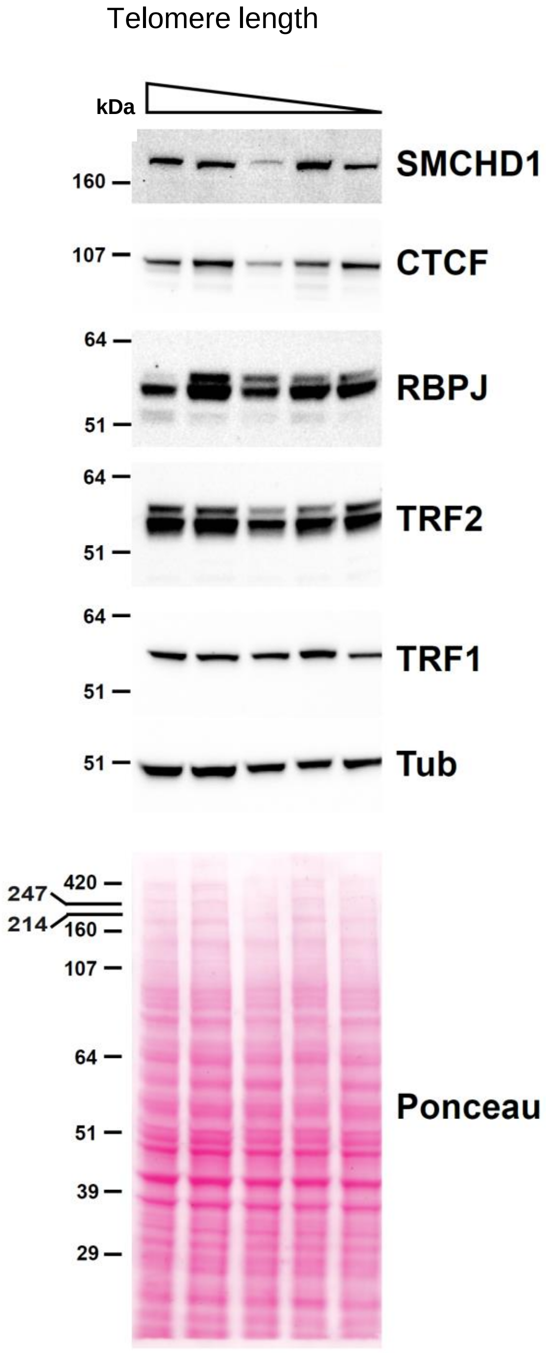

Supplemental Figure 19.
Western blots of proteins associated to telomeres (TRF1, TRF2, HOT1, TZAP, SMCHD1), chromatin remodeling (CTCF) or TPE-OLD (RBPJ) and Tubulin as loading control along with the associated ponceau whole membranes. We present 4 sets (biological quadruplicates) of serial Isogenic myoblasts clones with long and shorter average telomere lengths (i.e., from left to right : 12 – hTERT, 12, 10, 8, 6 kb; respectively). While the WB presented on the left were extracted with a NP40 lysis buffer, proteins on the right panel were extracted using a SDS based lysis buffer. We did not observed a change in the protein levels at either telomere length.

Supplemental Figure 20.

A

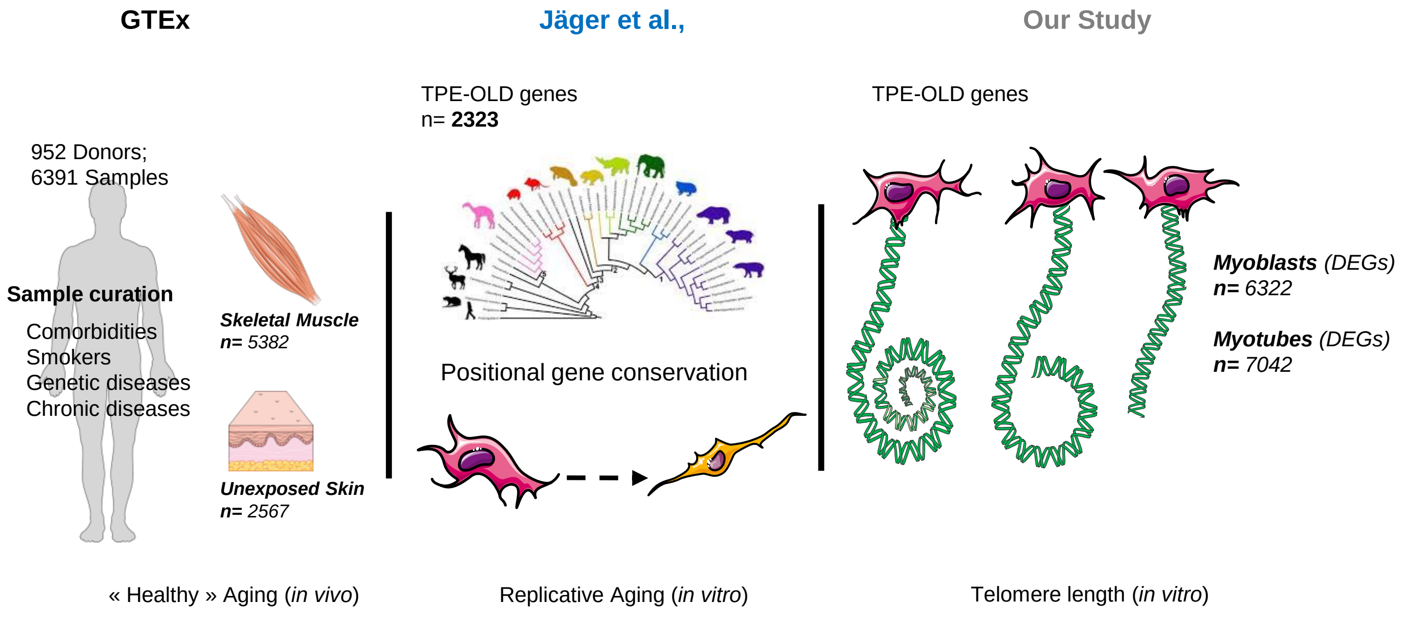

B

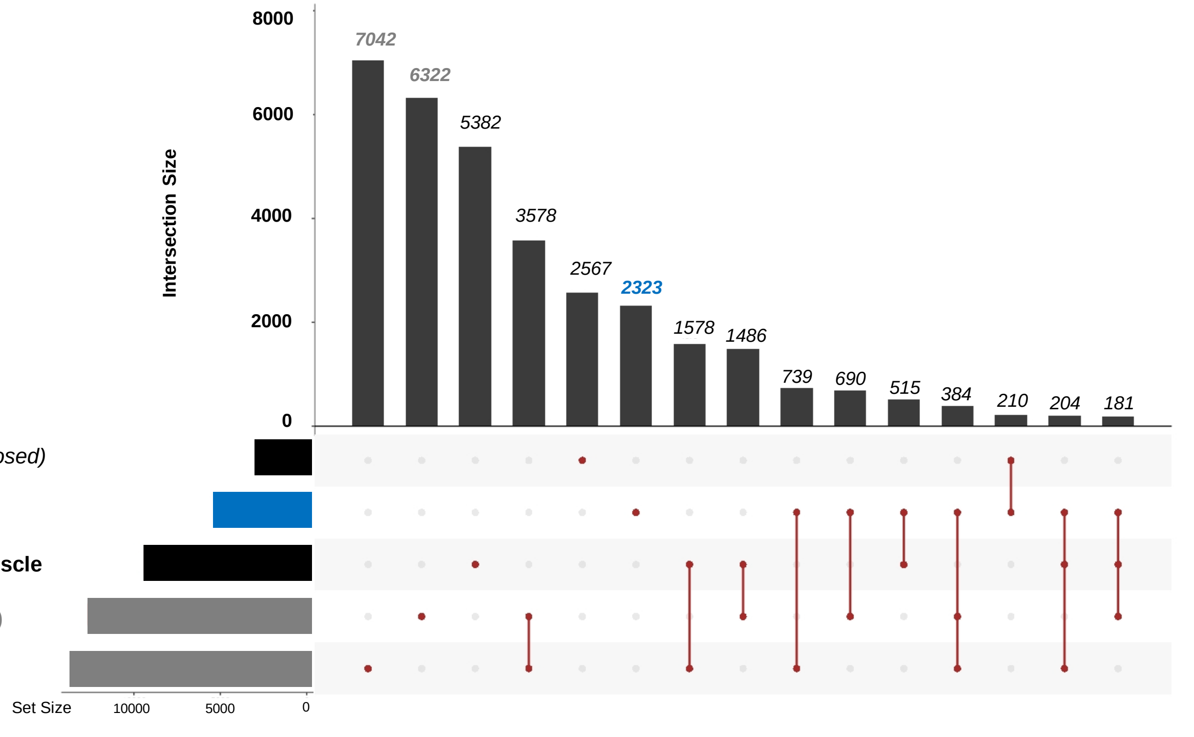

Supplemental Figure 20.
A. Graphical abstract briefly presenting available curated transcriptomic data from the GTEx project (left panel), Jäger and colleagues (middle panel) and our list of potential TPE-OLD genes (right panel). These lists (Supplemental Table S2) are used for comparisons across datasets, as presented in **B**. We report a Rplot combining results of various transcriptomic datasets. GTEx data were filtered for each tissue type (skeletal muscle and skin unexposed to sun) in two groups: short telomere (ST) group with 70-79 years old individuals and long telomere (LT) group with 20-29 years old individuals. Individuals did not die suddenly and/or had no chronic diseases that could impact their expression profiles. Overall we analyzed 82LT and 17ST skeletal muscle samples; 44LT and 15ST unexposed skin samples. Differential expression was established using DESeq2 package and the raw reads counts from the above-mentioned samples.
