# Supplementary material for: Telomere Position Effect‐Over Long Distances Acts as a Genome‐Wide Epigenetic Regulator Through a Common Alu Element
Source: Aging Cell. 2025 Mar 10;24(6):e70027. doi: 10.1111/acel.70027 (PMC12151916; doi:10.1111/acel.70027)
Supplement: Supplementary file 5 — Data S2. HiC pipeline and Primer list [file ACEL-24-e70027-s005.docx]

**HiC data-Mining Pipeline**

1. **#!/bin/bash**
2. set -eux
3. All_files=${1} #localization of the folder with data
4. cd ${All_files}
5. **for** folder **in** *
6. **do**
7. cd $folder
8. PREFIX=$folder
9. mv *_R1* ${PREFIX}_R1.fastq.gz
10. mv *_R2* ${PREFIX}_R2.fastq.gz
11. #Fastq to fasta
12. seqtk seq -a ${PREFIX}_R1.fastq.gz > ${PREFIX}_R1.fa
13. seqtk seq -a ${PREFIX}_R2.fastq.gz > ${PREFIX}_R2.fa
14. #Select reads with (TTAGGG)n
15. grep -B 1 -E "TTAGGGTTAGGGTTAGGGTTAGGGTTAGGG|TAACCCTAACCCTAACCCTAACCCTAACCC" ${PREFIX}_R1.fa > ${PREFIX}_R1.fa.forgrep || true
16. grep -B 1 -E "TTAGGGTTAGGGTTAGGGTTAGGGTTAGGG|TAACCCTAACCCTAACCCTAACCCTAACCC" ${PREFIX}_R2.fa > ${PREFIX}_R2.fa.forgrep || true
17. grep "^>" ${PREFIX}_R1.fa.forgrep > ${PREFIX}_R1.telorepeat || true
18. grep "^>" ${PREFIX}_R2.fa.forgrep > ${PREFIX}_R2.telorepeat || true
19. rm *.forgrep
20. #Put the same header between R1 and R2
21. sed -i -e 's/1:N/2:N/g' ${PREFIX}_R1.telorepeat || true
22. sed -i -e 's/2:N/1:N/g' ${PREFIX}_R2.telorepeat || true
23. #Get matching R1 in R2 and R2 in R1 using telomeric reads
24. grep -A 1 -f ${PREFIX}_R1.telorepeat ${PREFIX}_R2.fa > ${PREFIX}_grep_R1_in_R2 || true
25. grep -A 1 -f ${PREFIX}_R2.telorepeat ${PREFIX}_R1.fa > ${PREFIX}_grep_R2_in_R1 || true
26. #Remove telomeric reads obtained through the matching
27. **for** f **in** ./*grep*
28. **do**
29. grep -v -E "TTAGGGTTAGGGTTAGGGTTAGGGTTAGGG|TAACCCTAACCCTAACCCTAACCCTAACCC" $f > $f.notelo || true
30. **done**
31. cat ${PREFIX}_grep_R1_in_R2.notelo ${PREFIX}_grep_R2_in_R1.notelo > ${PREFIX}_total_read.txt
32. #Align reads interacting with telomeres
33. bowtie --un unmapped.txt --sam -p 10 -q -f /home/raphael/Desktop/reference_genome/hg38 ${PREFIX}_total_read.txt | samtools view -b > ${PREFIX}_total_read.fa.bam
34. samtools sort ${PREFIX}_total_read.fa.bam > ${PREFIX}_total_read_sort.bam
35. samtools index ${PREFIX}_total_read_sort.bam
36. bedtools bamtobed -i ${PREFIX}_total_read_sort.bam > ${PREFIX}total_read_sort.bed
37. rm *.fa
38. cd ..

**Primers**

***qPCR***

| **Target** | **Foward** | **Reverse** |
| --- | --- | --- |
| TRF2 | GTGGAAAAGCCACCCAGAGAAC | TGCAAAGGCTGCCTCAGAATCC |
| TZAP | CTTTCGGAAGGAGAACCTCCTG | TCCATCCTTCGGCGGAATGTCT |
| SMCHD1 | CAGCCAGTTCTTGAAGCAAGTGG | GCCTTGACAAGAGTTTACAGGGC |
| CTCF | GACCACACAAGTGCCATCTCTG | ATGTCGCAGTCTGGGCACTTGT |
| RBPJ | TCATGCCAGTTCACAGCAGTGG | TGGATGTAGCCATCTCGGACTG |

***Targeted Bisulfite sequencing***

| **Target** | **Foward** | **Reverse** |
| --- | --- | --- |
| Alu | GGATTATTTGAGGTTAGGAGAT | TCCCRAATAACTAAAACTACAA |
| TAR1 | GGAGTAGAGTTTTTTTTAGGTTAGATT | AAACAAACAATACCCCCAAC |

***Construction and ChIP-ddPCR***

| **Target** | **Foward** | **Reverse** |
| --- | --- | --- |
| GLIS2 | ATGAGGGCAAGGAACACCTG | AGGCACAGATCACTCATGCC |
| C16orf74 | CACCTCAGCCCAACAGAACT | AGCCAGGATGGTCTCGATCT |
| BET1L | AAAAGGCGCGCCGAAGCGATGGGCATATCTGTG | AAAAGGCGCGCCCCATCGTCTAGGCACTGAGC |
| GIPC3 | TCGCCTAGCAGAGGGTGAG | GGACCACAAATAAGGACAGGGAC |
| AluY | CGAGAACCATCCTGGCTAACA | ATCTCGGCTCACTGCAAGC |
| TAR1 | GGAGCAGAGTTCTTCTCAGGTCAGACC | AGGCAGGCAGTACCCCCAAC |
